# Functional diversification of *Paramecium* Ku80 paralogs safeguards genome integrity during precise programmed DNA elimination

**DOI:** 10.1371/journal.pgen.1008723

**Published:** 2020-04-16

**Authors:** Arthur Abello, Vinciane Régnier, Olivier Arnaiz, Romain Le Bars, Mireille Bétermier, Julien Bischerour

**Affiliations:** 1 Université Paris-Saclay, CEA, CNRS, Institute for Integrative Biology of the Cell (I2BC), Gif-sur-Yvette, France; 2 Université de Paris, Paris, France; National Cancer Institute, UNITED STATES

## Abstract

Gene duplication and diversification drive the emergence of novel functions during evolution. Because of whole genome duplications, ciliates from the *Paramecium aurelia* group constitute a remarkable system to study the evolutionary fate of duplicated genes. *Paramecium* species harbor two types of nuclei: a germline micronucleus (MIC) and a somatic macronucleus (MAC) that forms from the MIC at each sexual cycle. During MAC development, ~45,000 germline Internal Eliminated Sequences (IES) are excised precisely from the genome through a ‘cut-and-close’ mechanism. Here, we have studied the *P*. *tetraurelia* paralogs of *KU80*, which encode a key DNA double-strand break repair factor involved in non-homologous end joining. The three *KU80* genes have different transcription patterns, *KU80a* and *KU80b* being constitutively expressed, while *KU80c* is specifically induced during MAC development. Immunofluorescence microscopy and high-throughput DNA sequencing revealed that Ku80c stably anchors the PiggyMac (Pgm) endonuclease in the developing MAC and is essential for IES excision genome-wide, providing a molecular explanation for the previously reported Ku-dependent licensing of DNA cleavage at IES ends. Expressing Ku80a under *KU80c* transcription signals failed to complement a depletion of endogenous Ku80c, indicating that the two paralogous proteins have distinct properties. Domain-swap experiments identified the α/β domain of Ku80c as the major determinant for its specialized function, while its C-terminal part is required for excision of only a small subset of IESs located in IES-dense regions. We conclude that Ku80c has acquired the ability to license Pgm-dependent DNA cleavage, securing precise DNA elimination during programmed rearrangements. The present study thus provides novel evidence for functional diversification of genes issued from a whole-genome duplication.

## Author summary

DNA double-strand breaks (DSBs) are highly hazardous lesions, which, if incorrectly repaired, may cause cancer or trigger cell death. In spite of their toxicity, programmed DSBs are part of important physiological processes, such as the mixing of paternal and maternal alleles during meiosis or the generation of antibody diversity during the immune response that follows an infection. Here, we have studied how DNA breakage by a specific endonuclease and DSB repair by the non-homologous end joining pathway are coordinated during the programmed elimination of several thousands of short DNA fragments, which takes place at each sexual cycle in the ciliate *Paramecium tetraurelia*. We show that the product of one specific copy among three duplicates of the DSB repair *KU80* gene has the unique ability to tightly anchor the endonuclease in the nucleus and license programmed DNA cleavage at both ends of eliminated sequences. We identify the N-terminal domain of this Ku80 variant as the major determinant of its specific function. We propose that functional diversification of duplicated genes during evolution has allowed *P*. *tetraurelia* to secure precise programmed DNA elimination through the coupling of DSB introduction and repair.

## Introduction

Gene duplication and diversification have been considered a driving force for the evolution of organisms throughout the tree of life (reviewed in [1]) and different gene duplication mechanisms have been described. Segmental duplication is frequent in many organisms and affects a region of a chromosome harboring one or more genes, or part of a gene (domain duplication). Much more dramatic whole genome duplications (WGD) are rare events that lead to duplication of all the genes in the genome simultaneously and therefore have great potential for the emergence of novel functions (neo-functionalization, see [2]) or the subdivision of ancestral functions between the two duplicated gene copies (sub-functionalization, see [3,4]). The *Paramecium aurelia* group of ciliate species constitutes a remarkable system to study the evolutionary fate of duplicated genes. In these unicellular eukaryotes, at least three successive WGDs have occurred during evolution [5] and numerous gene duplicates from the intermediate and recent WGDs have been retained in extant diploid genomes (~25% and ~50% of pre-duplication genes, respectively) [5,6]. Very few examples of functional diversification of WGD duplicates, however, have been reported in these species [7]. Instead, extensive analyses of *Paramecium* genomes and gene expression levels have indicated that the vast majority of post-WGD gene pairs (also called ohnologs) have likely preserved their original function owing to gene dosage constraints [8] and tend to return to their initial single-copy state over time [5]. Within a pair of ohnologs, progressive dosage unbalance between duplicates is thought to allow pseudogenization and eventual loss of the copy with the lower expression level [9].

Like other ciliates, *Paramecium* species all exhibit a characteristic nuclear dimorphism (reviewed in [10]). The polyploid somatic macronucleus (MAC, 800n) is essential for gene expression but fragmented and eventually destroyed during sexual processes (conjugation between partners of compatible mating types or autogamy, a self-fertilization process occurring in the absence of a partner). Two identical germline micronuclei (MIC, 2n) undergo meiosis and transmit the parental genetic information to the next generation. In each offspring cell, a new MAC develops from a copy of the MIC through a process involving large-scale programmed genome rearrangements (PGR). In *P*. *tetraurelia*, PGR eliminate 25 to 30% of germline DNA, including numerous repeated sequences, such as transposable elements (TEs) or minisatellites, and ~45,000 short and noncoding Internal Eliminated Sequences (IESs), each found in the germline genome as a single-copy element—although most IESs have evolved from ancestral TEs [11,12]. IESs, flanked by one conserved TA dinucleotide at each end, are precisely excised from the somatic genome, leaving one TA at their excision site. Because IESs interrupt 47% of all genes in the germline, their efficient and precise excision is required for correct genome assembly and survival of sexual progeny. IES excision is mediated through the concerted action of six distinct domesticated PiggyBac transposases: one catalytic subunit (PiggyMac or Pgm) and five architectural partners (PgmL1 to PgmL5) that act together to cleave both DNA strands around each flanking TA [13–15]. Once IESs are removed, hundreds of thousands of DNA double strand breaks (DSBs) introduced in somatic chromosomes are massively repaired by the Ligase IV- and Xrcc4-dependent classical non-homologous end joining pathway (C-NHEJ). Direct ligation of IES-flanking broken DNA ends allows the precise closure of excision sites ([16], reviewed in [17,18]). Linear excised IES molecules are either directly circularized, also in a C-NHEJ-dependent manner, or form concatemers before circle formation and loss [19–21].

The C-NHEJ pathway has been conserved in all kingdoms of life and strictly depends upon the Ku protein, a key DSB repair factor that binds broken DNA ends, protects them against extensive resection and recruits downstream C-NHEJ proteins (reviewed in [22]). Most bacteria and archaea harbor a single *KU* gene encoding a protein homodimer, while the related *KU70* and *KU80* genes encode the two subunits of the eukaryotic Ku heterodimer. *KU70* and *KU80* were duplicated from a single ancestral *KU* gene and their products are not interchangeable within the heterodimer, providing an example of ancient functional diversification of duplicated genes [23]. Interestingly, some bacteria harbor multiple *KU* genes, which suggests that gene duplication and diversification have also occurred during bacterial evolution [24]. Eukaryotic Ku70 and Ku80 are each organized in three domains: a globular N-terminal α/β domain–absent from bacterial Ku proteins—exposed to the outer surface of the dimer, a core dimerization domain containing a β-barrel and the DNA binding ring, and a flexible C-terminal domain [25]. As a consequence of WGDs, *P*. *tetraurelia* harbors two *KU70* and three *KU80* genes [26]. *KU70a* and *KU70b* are recent WGD ohnologs and encode 98% identical proteins likely with redundant functions, but *KU70a* is consistently expressed at higher levels than *KU70b*. *KU80a* and *KU80b* are ohnologs from the most recent WGD, with constitutively low expression levels throughout the life cycle, while *KU80c*, a more distant duplicate from the intermediate WGD, is strongly and specifically induced during MAC development. Remarkably, *KU80c* expression is absolutely required for the licensing of Pgm-mediated DNA cleavage at IES ends, indicating that DNA cleavage and DSB repair are tightly coupled during PGR in *P*. *tetraurelia* [26].

In the present study, we have examined whether the essential need for *KU80c* during MAC development is simply due to its differential expression pattern relative to *KU80a* and *KU80b*, or whether the Ku80c protein itself, which shares only 74 and 73% identity with Ku80a and Ku80b, respectively, is specialized for IES excision. We first show that total intracellular Pgm levels remain unchanged upon RNAi-mediated *KU80c* knockdown (KD) and that Pgm still enters the developing new MAC. However, Pgm is no longer stably retained in the developing MAC in a *KU80c* KD, which correlates with genome-wide IES retention. We also report that developmentally induced overexpression of Ku80a - under the control of *KU80c* transcription signals—restores neither the development of a functional MAC nor IES excision in a *KU80c* KD. Our data indicate that, in addition to being specifically produced during MAC development, the Ku80c protein fulfills a specific function during PGR, allowing tight interaction of Pgm with chromatin. Using chimeric Ku80a/Ku80c fusions, we provide evidence that the N-terminal α/β domain of Ku80c (Ku80c_1-240_) is the major determinant conferring its functional specificity to the protein. We discuss how nuclear dimorphism and the pressure for efficient and precise IES excision in *Paramecium* may have contributed to the functional diversification of WGD paralogs involved in C-NHEJ-mediated DSB repair.

## Results

### Ku is essential for Pgm anchoring in the developing nucleus

**Fig 1:**
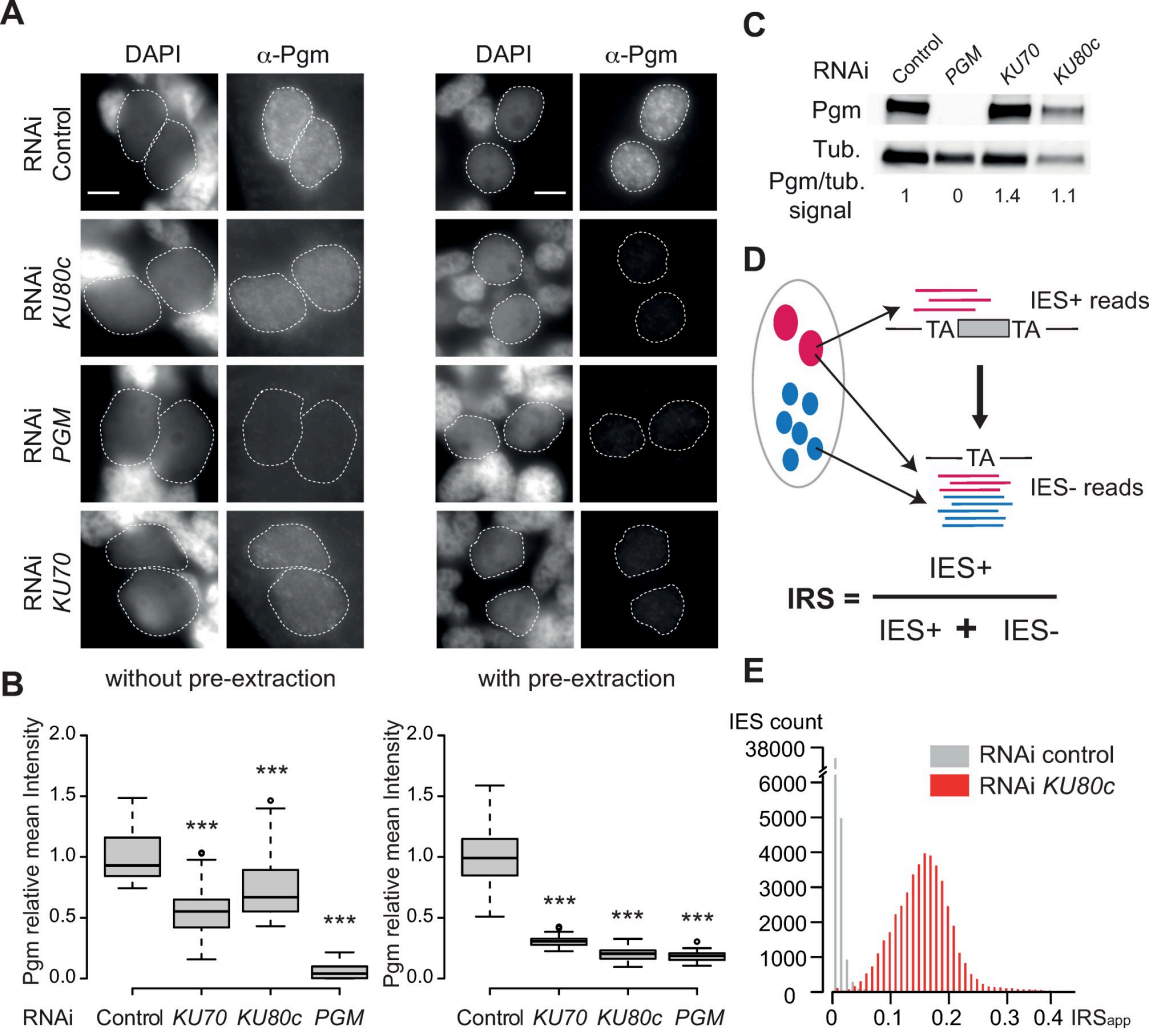
Expression and localization of Pgm upon *KU80c* and *KU70* RNAi. **(A)** Immunostaining of Pgm in early (T5-T10) autogamous cells subjected to control (L4440), *KU80c*, *PGM* or *KU70* RNAi. Immunostaining was performed with or without Triton pre-extraction. Scale bar is 5 μm. **(B)** Boxplots of Pgm mean fluorescence intensity in developing MACs of cells subjected to the different RNAi conditions. The size range of new MACs used for the analysis was defined as described in S1 Fig. **(C)** Western blot analysis of Pgm expression in early autogamous cells subjected to control (L4440), *PGM*, *KU70* or *KU80c* RNAi. Numbers at the bottom indicate the ratio between the Pgm and tubulin signals, normalized relative to the control. **(D**) Schematic representation of the IES+ and IES- Illumina sequencing reads that were counted to determine IES retention scores. **(E)** Distribution of IES retention scores in total genomic DNA extracted from late (T80) autogamous cells subjected to control (*ND7*) or *KU80c* RNAi. A replicate is presented in S2 Fig.

To gain insight into the molecular mechanisms underlying the activation of Pgm by Ku during IES excision, we quantitated the endogenous Pgm signal in Ku70- or Ku80-depleted cells, using immunofluorescence labelling with specific anti-Pgm antibodies [15]. Autogamous cells were fixed either with or without a Triton pre-extraction step (Fig 1A and 1B, S1 Fig), at a stage where IES excision takes place in control conditions (between T5 and T10, *i*.*e*. 5 to 10 hrs after the T0 time-point, which was defined as the stage when 50% of cells in the population harbor a fragmented old MAC). As shown previously [14], immunodetection without a pre-extraction step allows unbiased assessment of the Pgm nuclear signal. In contrast, immunodetection with a pre-extraction step, which removes the most labile proteins, reveals only conditions where Pgm is strongly associated to the nucleus. Cells were submitted to control, *PGM*, *KU70* or *KU80c* RNAi and the efficiency of each KD was monitored based on the absence of viable progeny with a functional new MAC (S1 Table). As judged from western blots (Fig 1C), total Pgm cellular amounts did not vary in *KU70* and *KU80* KDs relative to the control. In the absence of pre-extraction, the Pgm nuclear signal decreased moderately in Ku80c- and Ku70-depleted cells (25% and 44% decrease, respectively), whereas it completely vanished from control Pgm-depleted cells, as expected for efficient *PGM* KD (Fig 1A and 1B), indicating that Ku70/80c are not strictly required for Pgm localization *per se* in the new developing MAC. Strikingly, following Triton pre-extraction of the same cells, the Pgm nuclear signal dropped to the background level detected in a *PGM* KD. This phenotype, reminiscent of that observed upon depletion of any of the PgmL proteins, recently characterized as essential Pgm partners [14], suggests that Ku70 or Ku80c depletion weakens the association of Pgm with chromatin in the new developing MAC. Immunostaining of endogenous Pgm at a late autogamy time-point (T30), after IES excision is completed in control conditions, revealed that the Pgm signal reappears at this stage in the new MAC of Ku80c-depleted cells (S1 Fig). This observation is consistent with the previously reported aberrant up-regulation of *PGM* at late autogamy stages in a *KU80c* KD [26] or under other conditions impeding DNA rearrangements [27]. We conclude that during early autogamy, Ku70/80c, like PgmLs, contributes to Pgm retention in the new developing MAC, most likely through stabilizing the IES excision complex bound on chromatin.

### Ku80c is required for genome-wide IES excision

IES retention in a *KU80c* KD was documented previously for a small set of IESs using molecular approaches [26]. To extend this observation to the entire genome, we sequenced total genomic DNA from autogamous cells upon Ku80c depletion (see Materials and Methods). As a control, the same karyonidal population (*i*.*e*. a clonal population of cells issued from a single MAC differentiation event) was subjected to a KD against the non-essential *ND7* gene, which is not involved in DNA rearrangements [13,28]. IES retention scores (IRSs) were calculated following the mapping of Illumina sequencing reads on the MAC and MAC+IES reference genomes (Fig 1D and Materials and Methods) and analyzed using the ParTIES pipeline [29]. Of note, the new developing MAC co-exists in autogamous cells with numerous old MAC fragments harboring exclusively IES- forms. Here, genomic DNA was extracted from whole cells without partial purification of the developing new MACs, which can reduce more than 2-fold the proportion of IES+ over IES(+/-) forms and, therefore, introduce a general shift of the whole IRS distribution towards lower values relative to other published data [11–14]. As a consequence, IRSs cannot be considered as an absolute indicator of IES retention and should be referred to as apparent IES retention scores (IRS_app_). Taking this into account, we found that essentially no IES was significantly retained in the *ND7* KD control, whereas almost the entire set of IESs was retained in the two *KU80c* KD replicates, with an IRS mean value of 0.15. Even though we cannot exclude that partial excision may have occurred, we conclude that all IESs depend on Ku80c for their elimination (Fig 1E and S2 Fig).

### Pgm is dispensable for the stable localization of Ku80c in the new developing MAC

**Fig 2:**
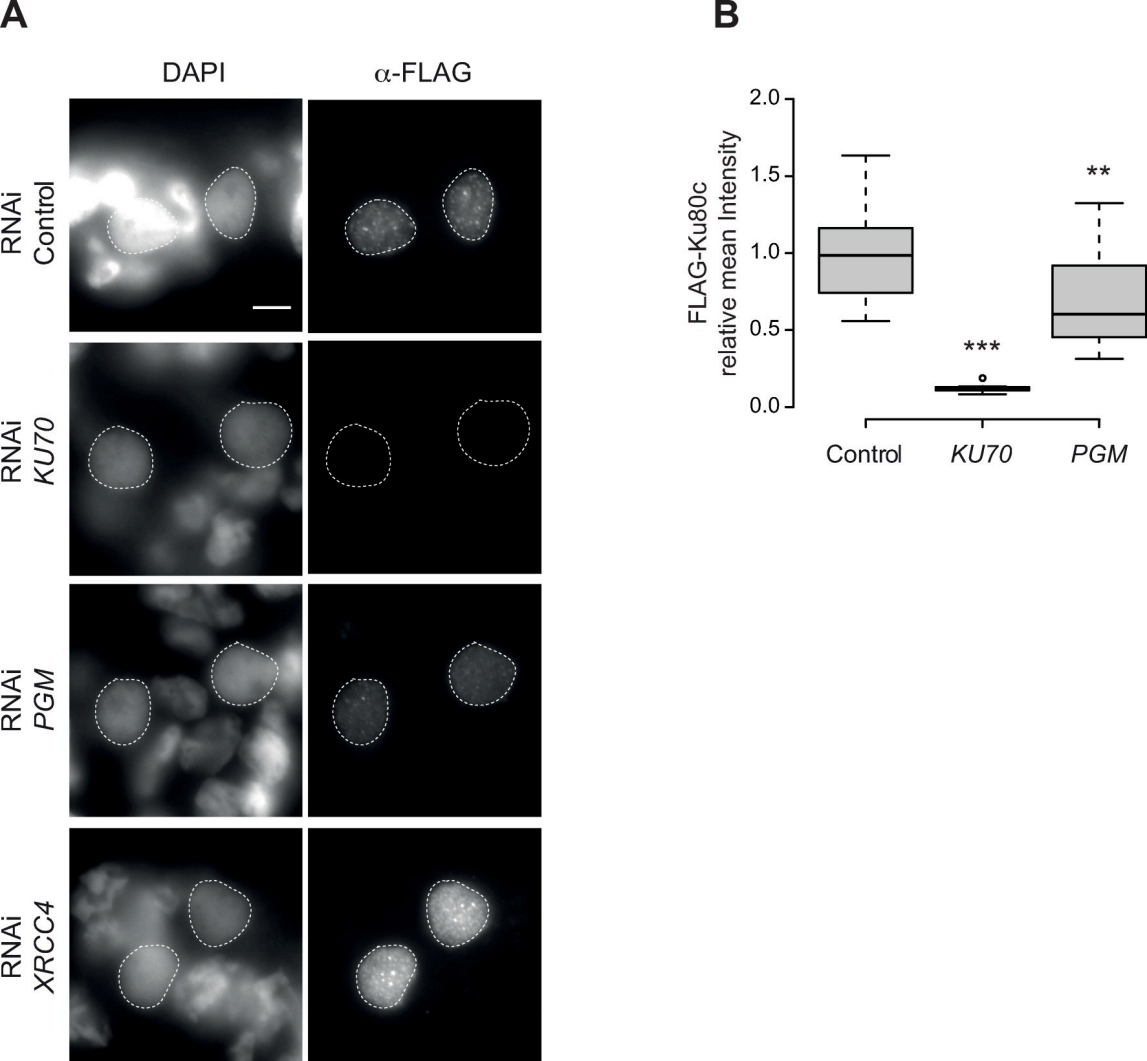
Expression and localization of FLAG-Ku80c in *KU70*, *PGM* and *XRCC4* RNAi. **(A)** Immunostaining of FLAG-Ku80c in early autogamous cells subjected to control (L4440), *KU70*, *PGM*, or *XRCC4* RNAi. Scale bar is 5 μm. **(B)** Boxplot of FLAG-Ku80c immunofluorescence intensity in developing MACs (see S3 Fig). For each condition, 12 to 20 MACs were analyzed. Quantification could not be performed for developing MACs subjected to *XRCC4* RNAi, since the same acquisition time was used for imaging all RNAi conditions, which led to saturated FLAG-Ku80c signal in the case of *XRCC4* RNAi.

As shown above, Pgm becomes weakly associated with the developing MAC when cells are depleted of Ku70/80c. Reciprocally, we investigated whether the nuclear localization of Ku80c and its sensitivity to Triton treatment are impacted by the depletion of Pgm. In the absence of specific antibodies against *P*. *tetraurelia* Ku proteins, we constructed a transgene expressing Ku80c fused to a 3xFLAG tag at its amino-terminal extremity (hereafter designated as FLAG-Ku80c), under the control of *KU80c* regulatory sequences. The FLAG signal was monitored by immunostaining of Ku70- or Pgm-depleted cells using the Triton pre-extraction protocol (Fig 2, S3 Fig). In the control, FLAG-Ku80c was observed exclusively in the new developing MAC and found to form foci, as reported previously for a GFP-Ku80c fusion [26]. In the *KU70* KD, the signal was strongly reduced, supporting the idea that Ku80c works in tight association with Ku70 inside the nucleus. In the *PGM* KD, in which DSBs are no longer introduced at IES ends [13], the FLAG-Ku80c signal was 70% as strong as the control signal, suggesting that a major fraction of Ku is still tightly retained in the nucleus without Pgm, *i*.*e*. even in the absence of Pgm-induced programmed DSBs. The missing signal may correspond to FLAG-Ku80c molecules stably bound to DNA ends during DSB repair in control conditions. To test the contribution of DSBs in stabilizing the nuclear localization of Ku80c, we analyzed Xrcc4-depleted cells, in which DSBs are introduced normally at IES ends, but are not repaired and accumulate in the developing new MAC [16]. Under these conditions, the FLAG-Ku80c signal strongly increases in the developing MAC, likely as a consequence of continuous recruitment of Ku80c to unrepaired DSBs.

### Developmental expression of Ku80a does not complement a *KU80c* KD

**Fig 3:**
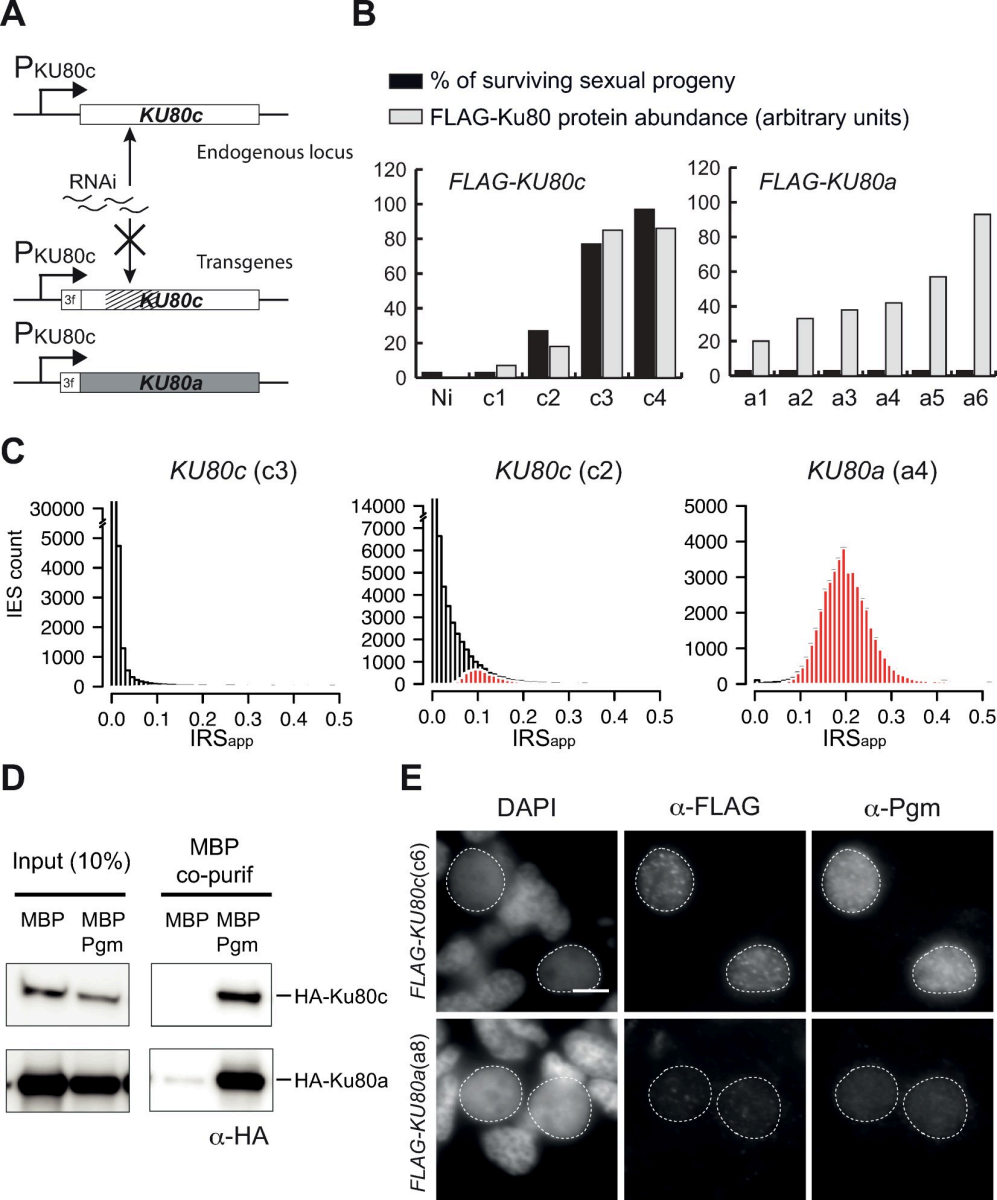
Ku80a does not rescue Ku80c-depleted cells during DNA rearrangements. **(A)** Schematic representation of the DNA tools used in complementation assays. The modified *KU80c* nucleic acid sequence that confers resistance to RNAi is represented by hatched lines. The *FLAG-KU80c* and *FLAG-KU80a* transgenes are under the control of the endogenous *KU80c* regulatory sequences. **(B)** Recovery of post-autogamous cells in *FLAG-KU80c* and *FLAG-KU80a* transformants. The percentage of surviving sexual progeny is in black. The relative abundance of FLAG-Ku80 (in gray) was quantified on western blots 5 hours after the beginning of autogamy and normalized by the tubulin signal (S6 Fig). **(C)** Distribution of IES retention scores in total genomic DNA extracted from late autogamous c3, c2 and a4 transformants subjected to *KU80c* RNAi. Significantly retained IESs relative to the control RNAi (*ND7*, see Fig 1D) are highlighted in red. **(D)** Pull down of HA-Ku80a and HA-Ku80c fusions with MBP-Pgm using recombinant proteins expressed in insect cells. In each panel, the HA-tagged protein that was co-expressed with MBP or MBP-Pgm is indicated on the right. MBP and MBP-Pgm expression and efficient precipitation were checked on western blots using anti-MBP antibodies (S7 Fig). **(E)** Co-immunostaining with α-FLAG and α-Pgm antibodies in early autogamous FLAG-Ku80c- (c6 transformant) or FLAG-Ku80a- (a8 transformant) expressing cells subjected to *KU80c* RNAi. Scale bar is 5 μm. Survival of the sexual progeny of each transformant and quantification of FLAG-Ku80 abundance are reported in S8 Fig.

In addition to their different expression patterns, the two groups of *P*. *tetraurelia KU80* paralogs issued from the intermediate WGD have diverged in their amino acid sequences: Ku80c shares 73% and 74% amino acid identity with Ku80a and Ku80b, respectively [26]. Interestingly, all *P*. *aurelia* species whose MAC genome has been sequenced [6,30–32] have retained at least one ohnolog of the Ku80a type and one of the Ku80c type, whereas more distant *Paramecium* species, which did not undergo the last two WGDs, each encode a single version of Ku80 (S4 & S5 Figs). The conservation of Ku80c across *P*. *aurelia* species suggests that the *KU80c* gene has diverged not only through the acquisition of a specific transcriptional pattern during MAC development, but also through the function of its encoded protein. To test this hypothesis, we examined whether Ku80a can substitute for a depletion of Ku80c during sexual processes. Because gene replacement is not straightforward in *Paramecium*, we used RNAi to knock down endogenous *KU80c* and provided Ku80a by microinjection of a transgene. The *KU80a* open reading frame was cloned under the control of *KU80c* regulatory sequences and fused at its 5’ end to sequences encoding the 3xFLAG peptide (Fig 3A and Materials and Methods). In parallel, silent mutations were introduced into the *KU80c* nucleic acid sequence to make it insensitive to RNAi (Fig 3A and S1 File). Each *FLAG-KU80a* and *FLAG-KU80c* transgene was microinjected individually into the MAC of vegetative cells and the resulting transformants were grown and starved to induce autogamy. Cells were collected 5 to 10 hrs following the T0 time-point and expression of FLAG-Ku80 was checked on western blots using anti-FLAG antibodies (Fig 3B, gray bars; S6 Fig). Transformants showing different expression levels for each fusion were selected for further analysis. In control non-injected cells (Ni) or in those transformants expressing the lowest levels of FLAG-Ku80c (c1 and c2), few if any viable post-autogamous progeny were recovered (Fig 3B, black bars). Higher expression levels allowed functional complementation and the recovery of progeny harboring a functional new MAC (c3 and c4), confirming that the FLAG-Ku80c fusion protein is functional. In contrast, at similar expression levels, FLAG-Ku80a failed to complement a *KU80c* KD. The same experiment was repeated several times and functional complementation was never observed (S1 Table). Moreover, expression of the *FLAG-KU80a* transgene exhibited a dominant negative phenotype in control KD conditions (S1 Table), raising the possibility that FLAG-Ku80a titrates out an essential partner of Ku80c, such as Ku70 for instance.

To gain more insight into the step at which Ku80a is defective during MAC development, we tested whether IES excision takes place normally in cells expressing FLAG-Ku80a and depleted of their endogenous Ku80c. We extracted total genomic DNA from late autogamous cells (~50 hrs following the T0 time-point) subjected to *KU80c* KD and expressing two different levels of FLAG-Ku80c (c2 and c3) or FLAG-Ku80a (a4), and performed Illumina sequencing of each sample (Fig 3C). We monitored IES retention scores for the three conditions and found that almost no IESs (7 out of 45,000) are retained in the fully complemented c3 clone, while 5710 are retained in the partially complemented c2 transformant. In contrast, a large majority (~44,000 out of 45,000) of IESs are significantly retained in a4 (Fig 3C), in which higher levels of Ku80 protein were expressed compared to c2. Taken together, these results demonstrate that FLAG-Ku80a is unable to promote IES excision.

### Ku80a can bind Pgm but does not promote its anchoring in the new MAC

Ku80a and Ku80c do not substitute for each other during autogamy and must, therefore, have different properties. In view of the previously reported interaction between Pgm and Ku80c [26], we speculated that Ku80a might be unable to interact with Pgm. To test this hypothesis, a recombinant HA-Ku80a fusion was co-expressed with MBP-Pgm in insect cells and the interaction between both proteins was tested using an MBP-pulldown assay (Fig 3D and S7 Fig). We found that both HA-Ku80a and HA-Ku80c co-precipitate with MBP-Pgm, ruling out that loss of interaction with Pgm provides a simple explanation for the functional difference between Ku80a and Ku80c.

**Fig 4:**
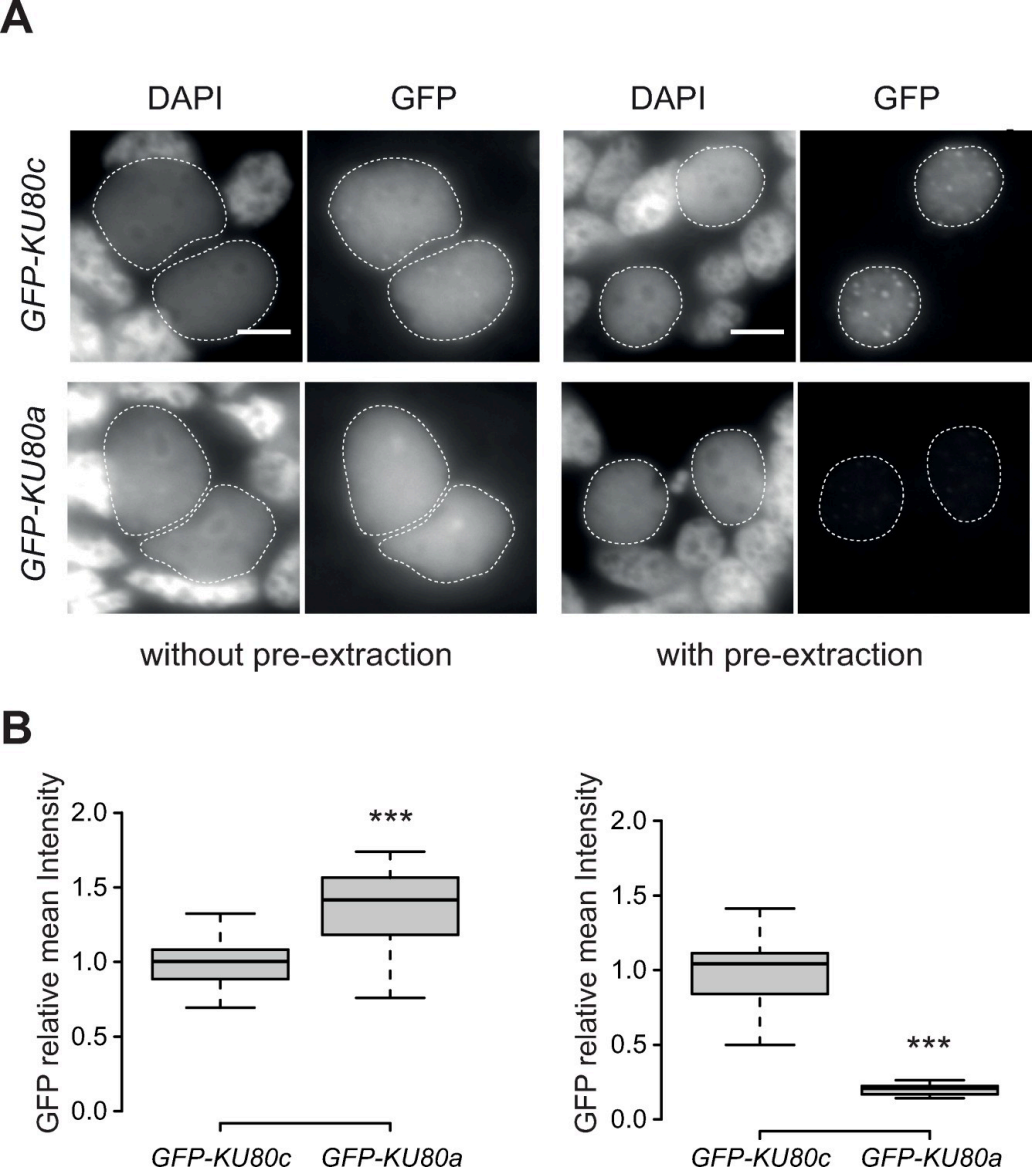
Nuclear localization of GFP-Ku80a and GFP-Ku80c fusions with or without Triton pre-extraction. **(A)** Early autogamous cells expressing GFP-Ku80c or GFP-Ku80a fusions imaged after paraformaldehyde fixation with or without a Triton pre-extraction step. Scale bar is 5 μm. Quantification of total cellular protein levels is shown in S10 Fig. **(B)** Boxplots of GFP fluorescence intensity in developing MACs from the cells shown in A, fixed without or with pre-extraction. For each fixation condition, the values were normalized relative to the mean signal obtained for the *GFP-KU80c* transformant. For each condition, 28 to 56 developing MACs were analyzed.

We further checked the localization of Ku80 and Pgm in the new MAC of Ku80c-depleted cells complemented with FLAG-Ku80a (a8) or FLAG-Ku80c (c6), using the Triton pre-extraction protocol (Fig 3E). FLAG-Ku80c-expressing cells showed a wild type localization pattern for both FLAG-Ku80c and Pgm, with an intense signal for each protein and a few large foci in the new developing MAC. In contrast, the FLAG-Ku80a and Pgm nuclear signals were severely reduced in FLAG-Ku80a-expressing cells depleted of their endogenous Ku80c (Fig 3E and S8 Fig). In order to infer whether Ku80a is deficient in entering the new developing MAC or in its stable nuclear association, we needed to compare its localization with or without the pre-extraction step. We found that FLAG immunodetection was not possible without the pre-extraction step, probably due to epitope masking (S10 Fig). We therefore switched to GFP-Ku80 fusions and confirmed that Ku80a and Ku80c had a localization pattern similar to their respective FLAG-Ku80 counterparts following Triton pre-extraction (Fig 4A). Omitting the Triton pre-extraction step, we found that GFP-Ku80a and GFP-Ku80c both localized efficiently in the new MAC (Fig 4 and S9 Fig), showing that Ku80a is efficiently imported into the new developing MAC. We conclude that Ku80a - in contrast to Ku80c - is released from the new MAC following Triton pre-extraction. Taken together, these data indicate that Ku80a only weakly associates with the new developing MAC and is therefore unable to stably anchor Pgm.

### The α/β domain of Ku80c plays a specific essential role during DNA rearrangements

**Fig 5:**
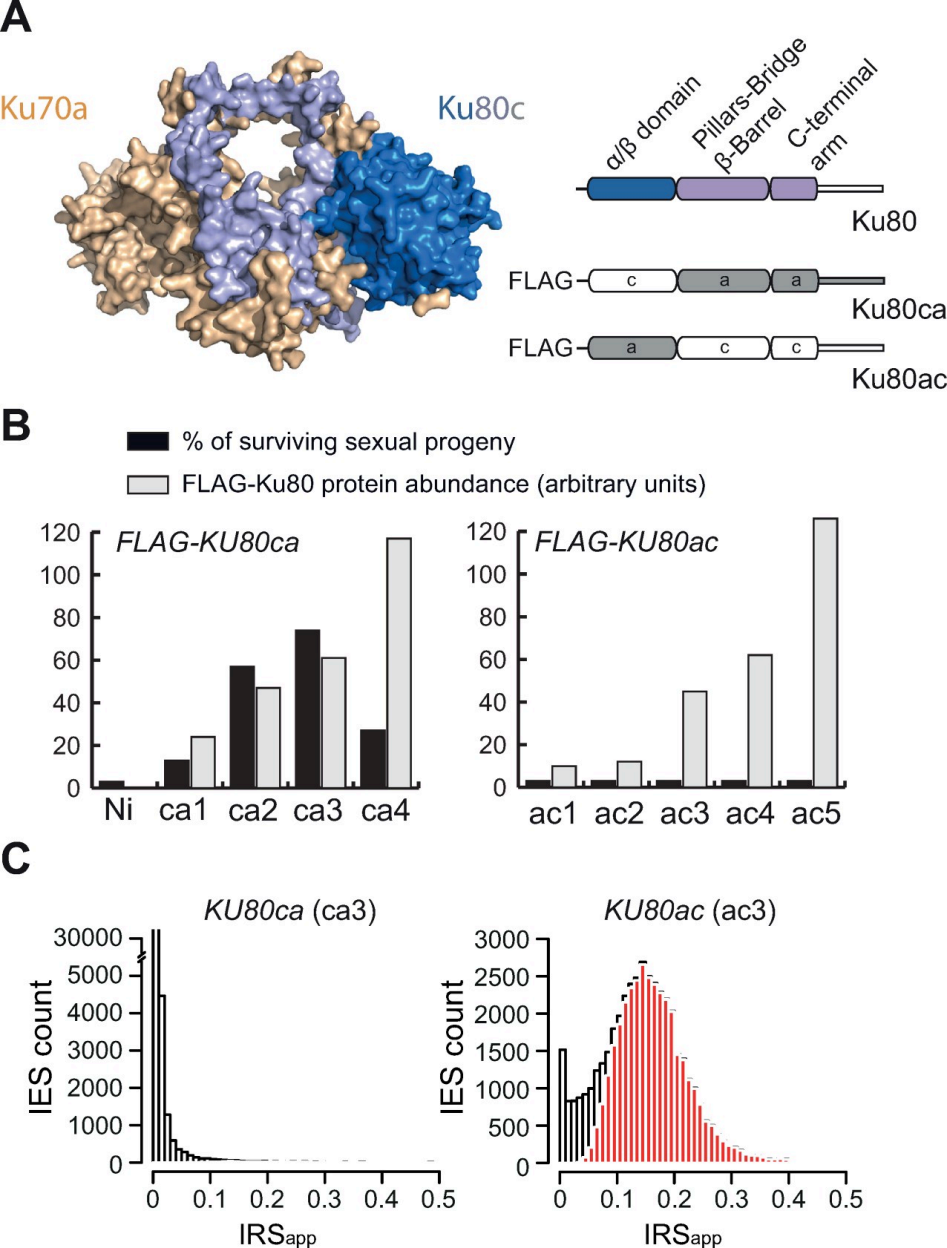
The Ku80c α/β domain fulfills an essential function during DNA rearrangements. **(A)** A structural model of the Ku70a/Ku80c heterodimer (left) was obtained using PHYRE2 v2.0 [57], with the N-terminal α/β domain of Ku80c in dark blue and its C-terminal part in light blue. A schematic representation of the chimeric constructs used in complementation assays is shown on the right. The *FLAG-KU80ca* and *FLAG-KU80ac* transgenes are expressed under the control of the *KU80c* regulatory sequences. **(B)** Recovery of post-autogamous cells in *FLAG-KU80ca* and *FLAG-KU80ac* transformants. The percentage of surviving sexual progeny is shown in black. The relative abundance of FLAG-Ku80 (in gray) was quantified on western blots 5 hours after the beginning of autogamy and normalized by the tubulin signal (S11 Fig). **(C)** Distribution of genome-wide IES retention scores in Ku80c-depleted cells complemented by FLAG-Ku80ca (ca3) or FLAG-Ku80ac (ac3). Significantly retained IESs relative to the control RNAi (*ND7* in Fig 1D) are highlighted in red.

The crystal structure of human Ku70/80 has revealed a domain organization comprising an amino-terminal α/β domain, a central β-barrel domain and a C-terminal helical arm (Fig 5A) [25]. MUSCLE alignment of all Ku80 amino acid sequences from *P*. *aurelia* species indicated that differences between the Ku80a and Ku80c groups are distributed along the sequence, without highlighting a particular domain that might account for their functional divergence (S4 Fig). To better define whether a specific domain is implicated in the functional specialization of Ku80c, we tested the activity of Ku80ca and Ku80ac chimeric proteins, in which the Ku80a and Ku80c α/β domains have been swapped (Fig 5A). Ku80ca carries the α/β domain of Ku80c (residues 1–240) fused to the C-terminal part of Ku80a (residues 241–739). Ku80ac is formed by the α/β domain of Ku80a (residues 1–240) and the C-terminal part of Ku80c (residues 241–737). Both chimeric constructs were expressed as N-terminal FLAG fusions in *P*. *tetraurelia* under the control of *KU80c* regulatory sequences and their expression was verified on western blots using anti-FLAG antibodies (Fig 5B gray bars and S11 Fig). We compared sexual progeny survival in a complementation assay, after cells expressing each chimeric protein were subjected to a *KU80c* KD during autogamy (Fig 5B black bars and S1 Table). In this assay, the Ku80ac chimera failed to complement a *KU80c* KD at any protein expression level that we tested. In contrast, expression of Ku80ca allowed complementation and restored up to 75% viable progeny in some transformants (Fig 5B). We noticed, however, that the offspring of FLAG-Ku80ca-complemented cells had slightly different phenotypes from those of FLAG-Ku80c-complemented cells, with post-autogamous survivors growing more slowly and exhibiting heterogeneous cell shapes. Nevertheless, they succeeded in resuming vegetative growth and, for this reason, were considered as viable sexual progeny (Fig 5B).

To gain more insight into the efficiency of IES excision in both samples, we purified genomic DNA from late autogamous cells (T50) expressing either Ku80ca (ca3, 73% viable progeny) or Ku80ac (ac3, 0% viable progeny) and performed Illumina high throughput sequencing of each sample (Fig 5C and S3 Table). IES retention scores were analyzed as described [29]. Almost all IESs were excised in Ku80ca-complemented cells, with 608 IESs being significantly retained, whereas a vast majority (36302 IESs) were retained in Ku80ac-complemented cells. We repeated the complementation experiment using a different series of transformants expressing FLAG-Ku80ca (S12 Fig). In this replicate, post-autogamous survivors again exhibited slow growth and altered cell shape. Genome sequencing of late autogamous cells originating from one transformant (ca8, 74% viable progeny) further confirmed that a small subset of IESs (961 IESs) were significantly retained (S13 Fig). Of note, these phenotypes are quite similar to the behavior of some *P*. *tetraurelia* mutants, in which excision of only a small subset of IESs is impaired [33]. When comparing the two replicates, we found that 83% of those IESs that are retained in ca3 are also retained in ca8 (S14 Fig), indicating that IESs that fail to excise in the presence of FLAG-Ku80ca represent a specific subset, which we hereafter designate as ‘Ku80ca-sensitive IESs’. Altogether, our results point to an essential role of the α/β domain in the functional specialization of Ku80c during genome rearrangements, while the C-terminal domain has limited impact on progeny survival and IES excision.

### Ku80ca-sensitive IESs share specific features

**Fig 6:**
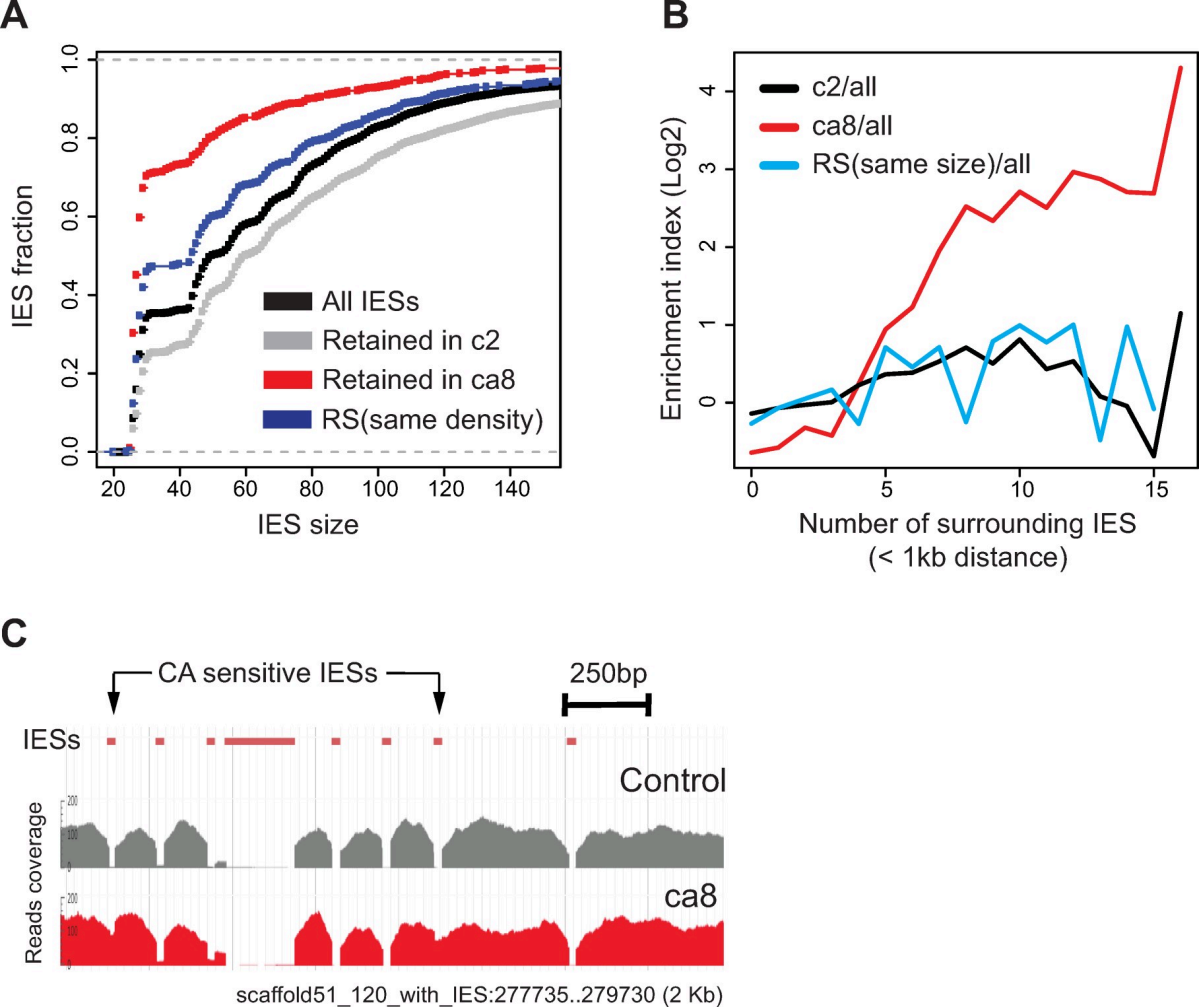
Analysis of IES retention in complemented cells reveals subtle functional differences between the C-terminal domains of Ku80c and Ku80a. **(A)** Cumulative distribution of IES lengths for the entire set of IESs (black) or the subset of retained IESs in c2 (partial complementation, see Fig 3) and ca8 complemented samples (see S12 Fig). Dark blue: cumulative distribution of IES lengths for a random sampling (RS) of 961 IESs showing the same density of neighboring IESs as the 961 IESs retained in Ku80ca-complemented cells. **(B)** IES density bias around retained IESs in cells complemented with Ku80ca (ca8) or partially complemented with Ku80c (c2). To calculate IES enrichment indexes, retained IESs from each dataset were classified in density subsets based on the number of surrounding IESs located at a <1kb distance and the fraction of retained IESs in each density subset (S16 Fig) was divided by the fraction of all IESs from the same subset. Black and red curves: enrichment index (Log2 scale) for c2 and ca8, respectively. Light blue: similar indexes calculated for a random sampling (RS) of 937 IESs showing the same size distribution as the 961 IESs retained in Ku80ca-complemented cells. **(C)** Screenshot of a 2-kb genomic region harboring 8 IESs, two of which are retained in Ku80ca-complemented cells (red) and fully excised in a control (gray; *ND7* KD in non-injected cells).

The experiments reported above reveal that a few hundred IESs (608 to 961 IESs according to replicates) are significantly retained in the zygotic MAC genome upon complementation of a *KU80c* KD with Flag-Ku80ca. We found that Ku80ca-sensitive IESs tend to be short, with 70% belonging to the first size peak (26–30 bp) compared to 30% for the reference set of all IESs (Fig 6A and S15 Fig, ca8 transformant). Moreover, using the ParameciumDB genome browser [34,35], we noticed that Ku80ca-sensitive IESs are often found in IES-rich genomic regions (Fig 6C). To confirm the latter observation at the genome-wide level, we classified IESs in density subsets based on the number of surrounding IESs within a 1-kb distance on either side, for both Ku80ca-sensitive IESs and all IESs. We found that for IES densities > 4–5, the fractions of Ku80ca-sensitive IESs were higher than expected (S16 Fig), as illustrated by the higher enrichment indexes of Ku80ca-sensitive IESs relative to all IESs (Fig 6B). However, not all IESs from IES-rich regions are Ku80ca-sensitive (Fig 6C), indicating that high density of neighboring IESs is not the sole determinant for poor excision in the presence of Ku80ca.

To test whether retention of Ku80ca-sensitive IESs is linked to globally reduced activation of Pgm in the presence of Ku80ca, we compared the characteristics of these IESs with those that fail to excise upon partial complementation of a *KU80c* KD with FLAG-Ku80c (c2 transformant in Fig 3). We found that 70% of Ku80ca-sensitive IESs are also retained when excision activity is reduced owing to limiting amounts of wild-type Ku80c (S14 Fig). In the latter case, however, we did not find the same size bias for retained IESs, among which short IESs even tend to be under-represented (Fig 6A). In addition, we observed no bias for retained IESs in IES-dense regions (Fig 6B). To test whether short IES size and location in IES-dense regions are linked in the reference set of all IESs, we computationally generated two different randomized samples (RS) sharing either the same number of surrounding IESs (same density, dark blue in Fig 6A) or the same size distribution (light blue in Fig 6B) as Ku80ca-sensitive IESs in the ca8 sample. We observed that a randomized sample of IESs with the same size distribution as Ku80ca-sensitive IESs (over-representation of short IESs) are not overrepresented in IES rich regions (Fig 6B), whereas a randomized sample of IESs with the same density bias in their surrounding regions as those of the ca8 sample do tend to be shorter (Fig 6A). We conclude from these experiments that the Ku80ca chimeric protein fails to properly excise a specific subset of IESs, which are located in IES-rich regions and, in part for that reason, tend to be short.

## Discussion

### Functional specialization of a WGD paralog

The present study of *KU80* genes in *P*. *tetraurelia* provides novel evidence for the functional divergence of paralogs originating from a WGD. *KU80a* and *KU80b*, constitutively expressed during vegetative growth and at all stages of the sexual cycle [26], likely encode housekeeping DSB repair proteins. Here, we show that Ku80a cannot replace Ku80c during MAC development even when expressed under *KU80c* transcription signals (Fig 3), demonstrating that the protein Ku80c itself has the unique property to license Pgm-dependent DNA cleavage during programmed rearrangements. The functional specialization of *KU80c* is further supported by the conservation of an orthologous, intermediate WGD *KU80* paralog in all species of the *P*. *aurelia* group (S5 Fig).

**Fig 7:**
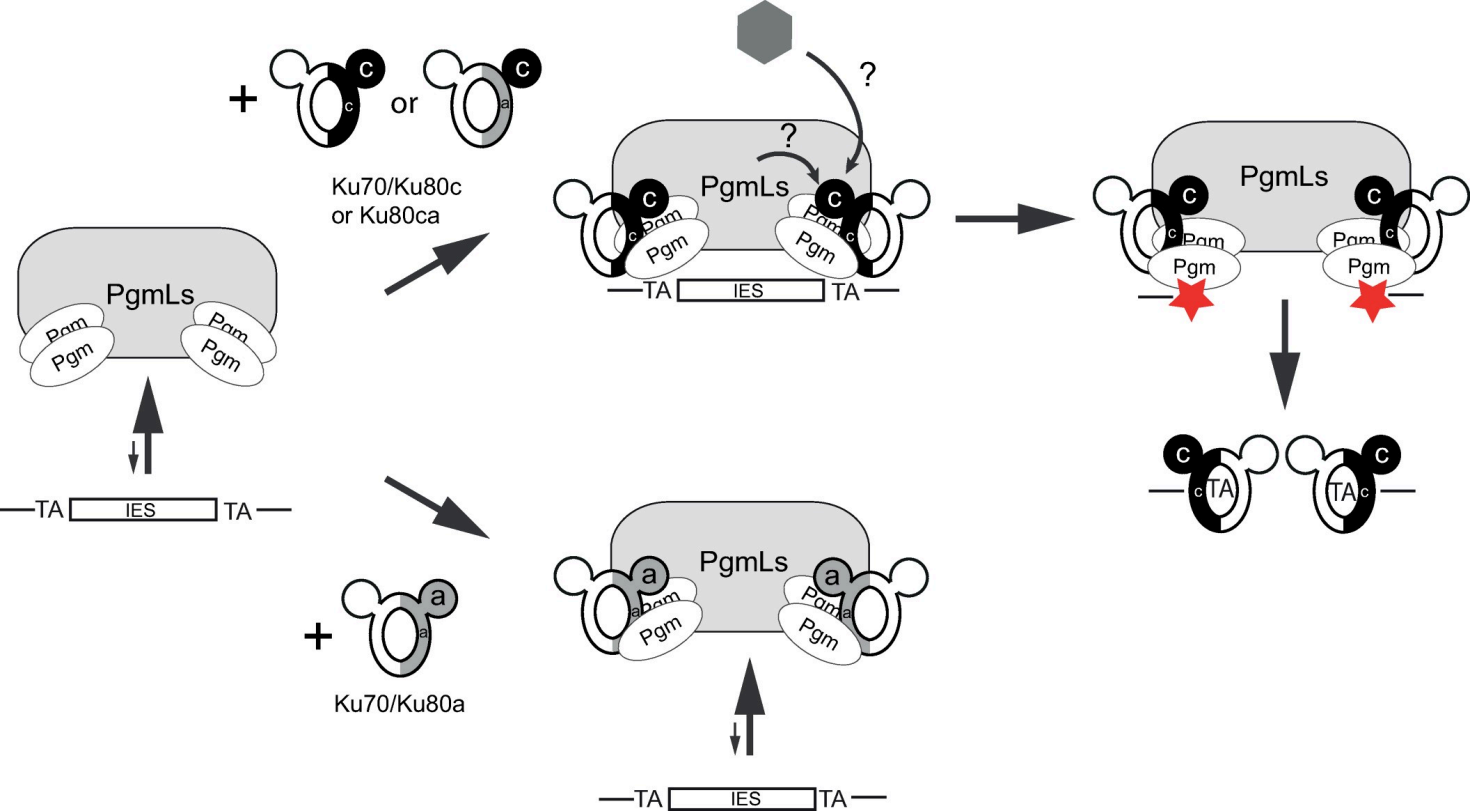
A model for Ku-dependent activation of Pgm. The Pgm/PgmLs complex is represented on the left, with arrows indicating that it binds poorly to IES DNA on its own. In the absence of detailed information on the stoichiometry of its different components, PgmLs are represented as a single box and two Pgm dimers are proposed to be positioned for IES end cleavage. On top, the two active Ku heterodimers identified in our study are shown, with Ku70 in white and Ku80c in black (black ball: α/β domain of Ku80c; black or gray half ring: C-terminal part of Ku80c or Ku80a, respectively). The association of Pgm/PgmLs with Ku70/Ku80c or Ku70/Ku80ca strengthens the interaction of the complex with DNA, through a mechanism that remains to be unraveled, but which involves the specific contribution of the α/β domain of Ku80c. Interactions of the Ku80c α/β domain with putative partners (PgmLs or other protein factors) are represented by arrows and question marks. We propose that Ku-mediated anchoring of Pgm/PgmLs to chromatin activates Pgm DNA cleavage activity (symbolized by red stars) and tightens the coupling between DSB introduction and repair by directly positioning Ku on broken DNA ends. At the bottom, Ku70/Ku80a is able to interact with Pgm/PgmLs but does not stabilize its interaction with IESs, resulting in defective IES excision.

We show here that Ku80c (and not Ku80a) anchors Pgm in the developing new MAC (Fig 3), even though both proteins can each interact with Pgm when co-expressed in a heterologous insect cell system. This result is reminiscent of our previous report that other Pgm partners, PgmL1 to PgmL5, all need to be present in the cell to promote the stable association of Pgm with the developing MAC and its full activity [14]. Consistent with Pgm anchoring taking place upstream of DNA cleavage, a catalytically dead Pgm_3A_-GFP fusion was shown to stably localize in the new MAC following Triton pre-extraction [15]. We propose a model, in which Ku80c, in association with Ku70, tightens the association of the Pgm/PgmL IES excision complex with chromatin, thereby allowing Pgm to cleave IES ends (Fig 7). The exact assembly order of the different components of the excision complex remains to be deciphered. In particular, it will be important to test the impact of *KU70* or *KU80c* KDs on the nuclear anchoring of PgmL proteins. We report here that Ku80c localizes stably in the new MAC in the absence of Pgm (Fig 2), consistent with the idea that tight association of Ku80c with chromatin, a property that is not shared by Ku80a, is a hallmark of its functional specialization. Future studies will address whether the stimulatory role of Ku80c on IES excision is only due to stabilized anchoring of Pgm in the new MAC or whether Ku80c further licenses DNA cleavage by inducing some conformational change of the Pgm catalytic site.

### Specific determinants for the functional specialization of Ku80c

All *P*. *tetraurelia* Ku80 paralogs share the same domain organization as their eukaryotic homologs, with an N-terminal α/β domain exposed on the outside of the Ku70/Ku80 heterodimer and a C-terminal part including the conserved β-barrel dimerization domain and DNA binding ring and a downstream flexible domain (Fig 5). The domain-swap experiment performed in the present study provides strong evidence that the α/β domain is a major determinant for the specific function of *P*. *tetraurelia* Ku80c during MAC development. Indeed, we found that the chimeric Ku80ca protein supports excision of a vast majority of IESs, while Ku80ac is mostly inactive (Fig 5). Because Ku80a and Ku80c both have the ability to interact with Pgm, we do not favor a discriminating role of their α/β domains in direct binding to Pgm. We propose instead that the Ku80c α/β domain specifically contributes to stabilizing Pgm on chromatin, either by interacting directly with a chromatin component or through a tri-partite interaction with another component of the excision complex or a yet unidentified chromatin-associated partner (Fig 7). The α/β domain of Ku80 (also called von Willebrand factor type A or vWA domain) was recently shown to interact with different partners, such as the accessory NHEJ factors APLF and XLF in humans [36,37] or the telomeric chromatin protein Sir4 in budding yeast [38], and may more generally act as a recruitment platform for Ku-interacting partners.

Close examination of IES retention scores in Ku80c-depleted cells complemented with wild-type Ku80c or the Ku80ca chimeric protein also points to a contribution of the C-terminal part of Ku80c in excision of a small subset of 600 to 1000 IESs, which are retained in Ku80ca-complemented cells (S13 Fig). These so-called ‘Ku80ca-sensitive’ IESs tend to be located in IES-dense regions of the genome (Fig 6). We speculate that multiple excision events in these regions may represent a challenge and require rapid turnover of the excision complex, which could be less easily achieved in the presence of Ku70/Ku80ca. During NHEJ-mediated repair, indeed, the Ku heterodimer remains trapped on DNA following ligation. We suspect that the C-terminal part of Ku80c plays a key role in its efficient release from DNA, consistent with a previous report that this region is involved in polyubiquitylation-dependent release from DNA [39]. In IES-dense regions, delayed release of Ku from DNA after excision of the first IESs might sterically inhibit subsequent excision of other IESs. Since we know that all IESs are not excised simultaneously [40], further work will address whether Ku80ca-sensitive IESs at these loci are excised at later stages during MAC development.

### Functional specialization of Ku80 paralogs enables precise DNA elimination during programmed genome rearrangements

One major implication of our study is that pressure to strongly couple DNA cleavage and DSB repair during programmed DNA elimination in *P*. *tetraurelia* has driven the functional specialization of Ku80c, one among the three WGD paralogs of Ku80. We propose that Ku-mediated nuclear anchoring of the Pgm/PgmLs excision complex to chromatin licenses DNA cleavage at IES ends and allows the straightforward recruitment of Ku70/Ku80c to the resulting broken ends, channeling them towards precise NHEJ repair (Fig 7). Given the large number of IESs within coding genes, the observed requirement for a specialized Ku heterodimer upstream of DNA cleavage represents an extreme example of coupling, which appears critical to avoid jeopardizing the integrity of the somatic genome during IES excision. In this regard, the C-terminal part of Ku80c may also have been optimized to carry out massive DNA excision in IES-dense regions. In the future, as germline genomes become available, the IES landscape of *Paramecium* species harboring a single *KU80* gene (S5 Fig) should help us determine whether Ku80c is an example of sub-functionalization following duplication of a bivalent ancestor or whether it is a rare example of *bona fide* neo-functionalization.

Different levels of coupling between DNA cleavage and DSB repair may exist in the related ciliate *Tetrahymena thermophila*. In this species, the vast majority of IESs are excised by Tpb2 [41], a Pgm-related domesticated transposase that introduces DSBs at variable positions around IES boundaries [42], which results in micro-heterogeneous IES excision junctions [43]. *T*. *thermophila* encodes a single *bona fide* Ku80 protein and the closure of its IES excision sites also depends upon the classical NHEJ pathway [44]. However, a *KU80* knockout does not abolish Tpb2-dependent DNA cleavage at IES ends and results in accumulation of unrepaired broken ends in the new MAC [44]. Thus, because most of ~10,000 *T*. *thermophila* IESs are located in intergenic or other non-coding regions [45], the pressure for precise excision may not be as strong as for *P*. *tetraurelia* IESs. Tpb2 likely interacts with Ku70/Ku80, because this appears to be a general property of PiggyBac transposases, be they domesticated or not (this work and [26,46]), but this interaction would simply direct the loading of Ku onto broken ends once DSBs have been introduced, which would represent a minimal form of coupling. Apart from Tpb2-dependent IESs, 12 intragenic *piggyBac*-derived IESs were described in *T*. *thermophila* [47,48]. These are excised precisely between their two 5’-TTAA-3’ flanking repeats by a distinct set of domesticated transposases, Tpb1 and Tpb6. Remarkably, Tpb1 and Tpb6 are protein fusions between a PiggyBac domesticated transposase and a Ku80-like protein, which may have originated from an ancestral gene duplication/fusion event [47,48]. Whether their N-terminal Ku80 domains have become specialized to license Tpb1/Tpb6-mediated DNA cleavage and carry out subsequent DSB repair during precise IES excision has not been established. However, these observations altogether suggest that quite a different evolutionary scenario may have taken place in *Tetrahymena* to secure the precise excision of its intragenic IESs, through physically linking a Ku80 paralog to an endonuclease.

We speculate that functional diversification of duplicated *KU* genes in ciliates and the pressure for precise IES excision may have favored the emergence of different types of coupling between DNA double-strand endonucleases and DSB repair factors, as a solution to face the threat represented by massive programmed rearrangements on genome integrity. Thus, work on ciliates may provide a general framework for the coupling between DNA cleavage and DSB repair during other programmed biological processes, such as V(D)J recombination of immunoglobulin genes in vertebrates or meiosis (discussed in [49]).

## Materials and methods

### *Paramecium* strains and culture conditions

*P*. *tetraurelia* wild-type 51 new [50] or its mutant derivative 51 *nd7-1* [15] were grown in a standard medium made of a wheat grass infusion inoculated with *Klebsiella pneumoniae* and supplemented with 0.8 μg/mL β-sitosterol and 100 μg/mL ampicillin [51]. Autogamy was carried out as described [15] and the progression of old MAC fragmentation and new MAC development was monitored using a Zeiss Lumar.V12 fluorescence stereo-microscope, following quick fixation and staining of cells in 0.2% paraformaldehyde 20 μg/mL DAPI.

### Gene knockdowns during autogamy

RNAi was achieved using the feeding procedure, as described [14,52]. *Paramecium* cells grown for 10 to 15 vegetative fissions in plasmid-free *Escherichia coli* HT115 bacteria [53] were transferred to medium containing non-induced HT115 harboring each RNAi plasmid and grown for ~4 additional divisions. Cells were then diluted into plasmid-containing HT115 induced for dsRNA production and allowed to grow for ~8 additional vegetative divisions before they starve and start autogamy. Final volumes were 50 to 100 mL for middle-scale experiments (western blotting, immunostaining and DNA extraction) and 0.5 to 1 L for large-scale experiments (whole-genome sequencing). The presence of a functional new MAC in the progeny was tested after four days of starvation, as described [15].

Control experiments were performed using the L4440 vector [54] or plasmid p0ND7c, which targets RNAi against the non-essential *ND7* gene [55]. RNAi plasmids were L4440 derivatives carrying the following inserts: PGM-1 (bp 873–1439 from *PGM*) [13], KU70a-1 (bp 514–813 from *KU70a*) and KU80c-2 (bp 557–1006 from *KU80c*) [26].

### Micro-injection of *FLAG-KU80* or *GFP-KU80* transgenes

All transgene-bearing pUC18 derivatives were linearized with appropriate restriction enzymes and co-injected with an *ND7*-complementing plasmid into the MAC of vegetative 51 *nd7-1* cells as described [15]. Sequences of the *FLAG-KU80* transgenes encoding N-terminal fusions of the 3X Flag tag (YKDHDGDYKDHDIDYKDDDDKT) to Ku80c or Ku80a are displayed in the S1 File. The *GFP-KU80c* transgene was described previously [26]. The sequences of the *GFP-KU80a* transgene and of the *KU80a/KU80c* domain-swap constructs are shown in S1 File.

Transgene injection levels (copy per haploid genome or cphg) was determined by qPCR on genomic DNA extracted from vegetative transformants, using a LightCycler 480 and the LightCycler 480 SYBR Green Master kit (Roche Diagnostics). Oligonucleotide primers for the *KU80a* and *KU80c* transgenes and the genomic reference locus are listed in S2 Table. Expressed Flag-Ku80 protein levels were determined on western blots for each transformant.

### Immunofluorescence analysis

Immunostaining of fixed cells using polyclonal anti-Pgm guinea pig antibody α-Pgm 2659-GP [15] or monoclonal anti-Flag antibody α-FLAG M2 (Sigma-Aldrich) was performed as described previously [14]. Unless otherwise specified, the protocol includes a Triton pre-extraction step prior to cell fixation. Observations were made using a Zeiss Axioplan 2 epifluorescence microscope with a 63x oil objective or an Olympus BX63 epifluorescence microscope with a 60x oil objective, focusing on the maximal area section of new developing MACs. Quantification of new MAC sizes and mean fluorescence intensities was performed using the ImageJ software (https://imageJ.nih.gov). We developed the MicMac_Epi.ijm ImageJ macro (https://github.com/Rom-LB/Mic-Mac) to automatically detect the new developing MACs among all nuclei present in autogamous cells, using a threshold based on green (and red when available) fluorescence. The macro delineates regions of interest (ROI) around the new MACs that can be adjusted manually using the DAPI fluorescence signal, if necessary. Fluorescence intensity analysis, boxplot representation and statistical analysis were performed as described [14]. All numerical values that were used for the boxplot representations can be found in S3 File. In all statistical analyses ** stands for p<0.01 and *** for p<0.0001 (Mann-Whitney-Wilcoxon statistical test).

### Protein extraction from *Paramecium* cells and western blot analysis

Approximately 6 x 10^4^ autogamous cells from middle-scale cultures were collected by centrifugation at T5-T10, washed with Dryl's buffer (2 mM sodium citrate, 1 mM NaH_2_PO_4_, 1 mM Na_2_HPO_4_, 1 mM CaCl_2_) and aliquoted before freezing in liquid nitrogen. Aliquots of ~1.5 x 10^4^ frozen concentrated cells were directly lysed following addition of an equal volume of boiling 10% SDS containing 1x Protease Inhibitor Cocktail Set 1 (Merck Chemicals) and incubation at 100°C for 3 min. SDS-PAGE, western blotting with α-Pgm 2659-GP, α-Flag M2 (Sigma), α-GFP (Roche Diagnostics) or α-alpha Tubulin TEU435 [56] antibodies and quantification of chemiluminescence signals were performed as described [14,15].

### Co-precipitation of proteins expressed in insect cells

Recombinant baculoviruses expressing MBP-Pgm, MBP alone and HA-Ku80c were produced in High Five cells as described [15,26]. A synthetic *KU80a* gene adapted to the universal genetic code (Eurofins Genomics) was cloned into the pFastBAC vector (ThermoFisher Scientific) and fused at its 5’ end to a nucleotide sequence encoding the HA tag (S2 File). Production of recombinant baculoviruses was performed using the BAC-to-BAC baculovirus expression system (ThermoFisher Scientific).

To co-express each HA-Ku80 fusion with MBP-Pgm (or the MBP control), High Five cells were co-infected with the appropriate recombinant baculoviruses. Cell lysis, preparation of soluble protein extracts, co-precipitation on amylose beads and detection of HA-tagged proteins on western blots using HA-7 monoclonal α-HA antibodies (Sigma Aldrich) were performed as described [15]. We showed previously that the HA epitope does not interact with MBP-Pgm on its own [14].

### Localisation of GFP-Ku80 fusions

Autogamous cells harboring *GFP-KU80* transgenes were fixed for 10 min in PHEM (60 mM PIPES, 25 mM Hepes, 10 mM EGTA, 2 mM MgCl_2_ pH 6.9) + 1.2% paraformaldehyde, washed once in TBST (10 mM Tris pH 7.4, 0.15 M NaCl, 0.1% Tween20) + 3% BSA, and stained with 0.5 μg/ml DAPI (Sigma Aldrich). Alternatively, cells were extracted with ice-cold PHEM + 0.7% Triton during 4 min, and fixed for 10 min in PHEM +1.2% paraformaldehyde + 0.3% Triton before the final TBST wash and DAPI staining.

### High throughput sequencing and analysis of IES retention

For each condition, total genomic DNA of late autogamous cells (4 days of starvation) was extracted from large-scale cultures as described [50] or from middle-scale cultures using the NucleoSpin Tissue extraction kit (Macherey Nagel). DNA was sequenced at a 76 to 160X coverage by a paired-end strategy using Illumina HiSeq (paired-end read length: 2x100 nt) or NextSeq (paired-end read length: 2x~75 nt) sequencers (S3 Table). Sequencing reads were mapped against the MAC or MAC+IES reference genomes of *P*. *tetraurelia* 51 [11] and IES retention scores (see S3 File) were calculated using the MIRET module of the ParTIES package [29]. A statistical test for the significance of each IES retention score was performed as described [14], using the *ND7* RNAi control as a reference (Fig 1).

## Supporting information

S1 Fig Quantification of mean immunofluorescence intensity *vs* developing MAC size in cells subjected to control, *PGM*, *KU70* or *KU80c* RNAi. **(A**) At the early time point (without pre-extraction), quantification was performed for developing MAC sizes ranging between 55–110 μm^2^ at their maximal area section, which corresponds to the peak of Pgm signal in the control. **(B)** For early autogamous cells that were fixed following pre-extraction, quantification was performed for developing MAC sizes ranging between 40–75 μm^2^. **(C)** Immunostaining of Pgm in late (T30) autogamous cells subjected to control (L4440), *KU80c* and *PGM* RNAi. **(D)** At the late time point, all developing MACs larger than 75 μm^2^ were analyzed. **(E)** Boxplots of Pgm mean fluorescence intensity in developing MACs of late autogamous cells shown in D. From 27 to 76 developing MACs were quantified for each RNAi.
(PDF)

S2 Fig Distribution of IES retention scores in Ku80c-depleted cells. **(A)** Distribution of IES retention scores in total genomic DNA extracted from late autogamous cells subjected to control (*ND7*) or two replicates of *KU80c* RNAi. Data obtained with ND7_r2 and KU80c_r2 samples are also displayed in Fig 1D. Significantly retained IESs in *KU80c* knockdowns relative to the *ND7* RNAi control are highlighted in red. **(B)** Spearman correlation plot of *KU80c* RNAi replicates.
(PDF)

S3 Fig Plot of FLAG-Ku80c mean immunofluorescence intensity *vs* developing MAC size in cells subjected to control, *PGM*, or *KU70* RNAi. Quantification was performed for developing MAC sizes ranging between 25–60 μm^2^ at their maximal area section, which corresponds to the peak of the Flag signal in the control RNAi (see Fig 2).
(PDF)

S4 Fig Alignment of ciliate Ku80 proteins. The analysis includes 39 amino acid sequences of Ku80 proteins or protein domains from different *Paramecium* species, *Tetrahymena thermophila* and *Homo sapiens*. Full-length sequences were used for the alignment, except for *P*. *polycaryum* Ku80 (PPOLY.Hb20-6.1.P0260103: residues 1–735) and the Ku80 domains of *T*. *thermophila* Tpb1 and Tpb6 (residues 1–704 and 1–709, respectively). Amino acid sequences were aligned using MUSCLE (http://www.ebi.ac.uk/Tools/msa/muscle/). Accession numbers of *P*. *tetraurelia* proteins: Ku80a (PTET.51.1.P1460025), Ku80b (PTET.51.1.P1510135), Ku80c (PTET.51.1.P1140146). Complete accession numbers can be found in S5 Fig. Note that *P*. *novaurelia* also encodes Ku80c/d proteins, which were not included in the alignment because their full sequence could not be deduced from the current assembly of the somatic genome.
(PDF)

S5 Fig Maximum Likelihood tree of ciliate Ku80 proteins. The tree includes 39 amino acid sequences of Ku80 proteins or protein domains from different *Paramecium* species and from *Tetrahymena thermophila*. Human Ku80 was used as an outgroup to root the tree. To construct the tree, the alignment of S4 Fig was edited to remove specific insertions restricted to 1 to 3 sequences only. All accession numbers are indicated. *P*. *tetraurelia* proteins are in red. The evolutionary history was inferred by using the Maximum Likelihood method based on the JTT matrix-based model [58]. The tree with the highest log likelihood (-4384.20) is shown. The percentage of trees in which the associated taxa clustered together is shown next to the branches. Initial tree(s) for the heuristic search were obtained automatically by applying Neighbor-Join and BioNJ algorithms to a matrix of pairwise distances estimated using a JTT model, and then selecting the topology with superior log likelihood value. A discrete Gamma distribution was used to model evolutionary rate differences among sites (5 categories (+G, parameter = 1.7816)). The tree is drawn to scale, with branch lengths measured in the number of substitutions per site. There were a total of 208 positions in the final dataset. Evolutionary analyses were conducted in MEGA7 [59]. The Ku80a/b and Ku80c/d groups of ohnologs from *P*. *aurelia* species are highlighted by colored boxes.
(PDF)

S6 Fig Western blot analysis of Pgm and FLAG-Ku80 expression levels in early autogamous cells subjected to *KU80c* RNAi. For the *FLAG-KU80a* and *FLAG-KU80c* transformants shown in Fig 3, total protein extracts were prepared at T5 during autogamy. FLAG-Ku80 proteins were revealed on western blots using α-Flag antibodies and the signal was normalized by the tubulin signal (see Fig 3B).
(PDF)

S7 Fig Co-precipitation of MBP-Pgm with HA-Ku80a and HA-Ku80c. Whole pictures of the western blots shown in Fig 3D. Detection of co-immunoprecipitated HA-Ku80 was performed first using α-HA antibodies (top panels). Following membrane stripping, expression of MBP fusions in all samples was checked using α-MBP antibodies (bottom panels: the residual post-stripping HA signal is marked with an asterisk). Dotted lines delimit the lanes that were used in Fig 3D. The five central lanes of each panel are unrelated to the present study.
(PDF)

S8 Fig Controls of the injection experiment shown in Fig 3E. **(A)** Detection of FLAG-Ku80 expression in *FLAG-KU80c* and *FLAG-KU80a* transformants on western blots. Transformants c6 and a8 were picked for further quantitative immunofluorescence analysis. **(B)** Survival of the sexual progeny and quantification of the Flag signal relative to the Tub signal from the western blots shown in A. **(C)** Boxplots of FLAG-Ku80 (left panel) and Pgm (right panel) immunofluorescence intensities in developing MACs of early autogamous cells from transformants c6 and a8 subjected to *KU80c* RNAi (see panel D). In the right panel, the first two samples correspond to non-injected cells subjected to control RNAi (L4440: Control) or *KU80c* RNAi (KU80c KD). **(D)** Plots of FLAG-Ku80 (left panel) and Pgm (right panel) immunofluorescence intensities. Quantification for the boxplots shown in C was performed for developing MAC sizes ranging between 35–65 μm^2^ at their maximal area section, which corresponds to the peak of Pgm in non-injected cells subjected to control RNAi.
(PDF)

S9 Fig Analysis of GFP-Ku80c and GFP-Ku80a expression in early autogamous transformants subjected to control (L4440) RNAi. **(A)** Western blot analysis. Total protein extracts were prepared at T5 during autogamy. GFP-Ku80 proteins were quantified on western blots using α-GFP antibodies and the signal was normalized relative to the tubulin signal. Transformants c13 (GFP-Ku80c) and a5 (GFP-Ku80a) were picked for further analysis. **(B)** Plots of GFP fluorescence intensities in new MACs. Quantification for the boxplots shown in Fig 4B was performed for developing MAC sizes ranging between 55–110 μm^2^ or 40–65 μm^2^ at their maximal area section, in cells that were treated without (left panel) or with (right panel) Triton pre-extraction, respectively. These size windows correspond to the peak of GFP in GFP-Ku80c-expressing cells. The nuclear localization of GFP-Ku80c (c13) and GFP-Ku80a (a5) is analyzed in Fig 4.
(PDF)

S10 Fig Omitting the pre-extraction step prevents immunodetection of Flag-Ku80c in fixed autogamous cells. Immunostaining of FLAG-Ku80c in early autogamous cells (T5-T10) subjected to RNAi against their endogenous *KU80c* was performed after the cells were prepared with (bottom panel) or without (top panel) Triton pre-extraction. Scale bar is 5 μm.
(PDF)

S11 Fig Western blot analysis of FLAG-Ku80 expression in early autogamous cells from *FLAG-KU80* transformants subjected to *KU80c* RNAi. Total protein extracts were prepared at T5 during autogamy. FLAG-Ku80 proteins were quantified on western blots using α-Flag antibodies and the signal was normalized by the tubulin signal. The results of the quantification are shown in Fig 5B.
(PDF)

S12 Fig Replicate of the complementation experiment using *FLAG-KU80c* and *FLAG-KU80ca* transgenes. **(A)** Recovery of post-autogamous cells from *FLAG-KU80c* and *FLAG-KU80ca* transformants (replicates of experiments shown in Figs 3B and 5B). For each clone the percentage of surviving sexual progeny is shown in black. The relative abundance of FLAG-Ku80 (in gray) was quantified on western blots 5 hours after the beginning of autogamy and normalized by the tubulin signal. **(B)** Western blot analysis of FLAG-Ku80 expression in early autogamous *FLAG-KU80c* and *FLAG-KU80ca* transformants subjected to *KU80c* RNAi. The ca8 transformant was used for the genome-wide analyses shown in Fig 6.
(PDF)

S13 Fig Distribution of IES retention scores in total genomic DNA extracted from late autogamous *FLAG-KU80c* or *FLAG-KU80ca* transformants subjected to RNAi against endogenous *KU80c*. Left panels: Retention scores were plotted for all IESs in the genome, with significantly retained IESs (relative to the control *ND7* RNAi) highlighted in red. Right panels: Distribution of significantly retained IESs in each experiment shown on the left.
(PDF)

S14 Fig Venn diagram of significantly retained IESs in *FLAG-KU80c* or *FLAG-KU80ca* transformants c2, ca3 and ca8. The sets of significantly retained IESs in transformants c2, ca3 and ca8 subjected to *KU80c* RNAi are displayed in S13 Fig.
(PDF)

S15 Fig Analysis of the length of retained IESs in *FLAG-KU80c* or *FLAG-KU80ca* transformants c2 and ca8. **(A)** IES length distributions for all IESs (left) and significantly retained IESs in c2 (middle) and ca8 (right) transformants subjected to *KU80c* RNAi. **(B)** Relative distribution of IES lengths for all IESs (black) and significantly retained IESs in c2 (dark gray) and ca8 (light gray). Only IESs shorter than 150 bp were taken into account to draw the graphs.
(PDF)

S16 Fig Analysis of IES density in the vicinity of retained IESs in *FLAG-KU80c* or *FLAG-KU80ca* transformants c2 and ca8. For each condition (ca8 or c2 transformants subjected to *KU80c* RNAi), retained IESs were classified in density subsets based on the number of surrounding IESs located at a <1kb distance, and the fraction represented by each subset relative to all retained IESs was calculated. For all IESs, the total fraction of IESs in each density subset is represented (black bars).
(PDF)

S1 File Sequences of *FLAG-KU80* and *GFP-KU80* plasmids used in micro-injection experiments. *KU80c* regulatory sequences are in blue italics, coding sequences are in capital letters, with the 3X Flag- or GFP-coding sequence in green, *KU80* sequences in capital italics (original ATG initiation codons are underlined) and the modified RNAi-resistant insert in red. Transgene initiation and stop codons are in bold.
(DOCX)

S2 File Sequence of the pFastBAC derivative harboring the *HA-KU80a* used for protein expression in insect cells. (DOCX)

S3 File IES retention score and immunofluorescence quantification data. (XLSX)

S1 Table Summary of all RNAi experiments. (XLSX)

S2 Table Oligonucleotides used in qPCR. (XLSX)

S3 Table Sequencing data with ENA accession numbers. (XLSX)
