# Supplementary figures and images for: Functional diversification of Paramecium Ku80 paralogs safeguards genome integrity during precise programmed DNA elimination
Source: PLoS Genet. 2020 Apr 16;16(4):e1008723. doi: 10.1371/journal.pgen.1008723 (PMC7161955; doi:10.1371/journal.pgen.1008723)

**A**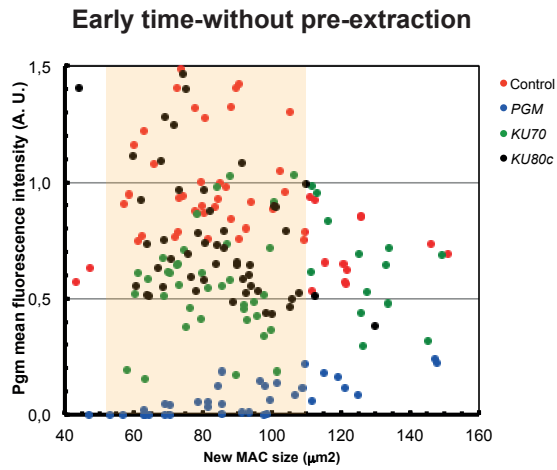**B**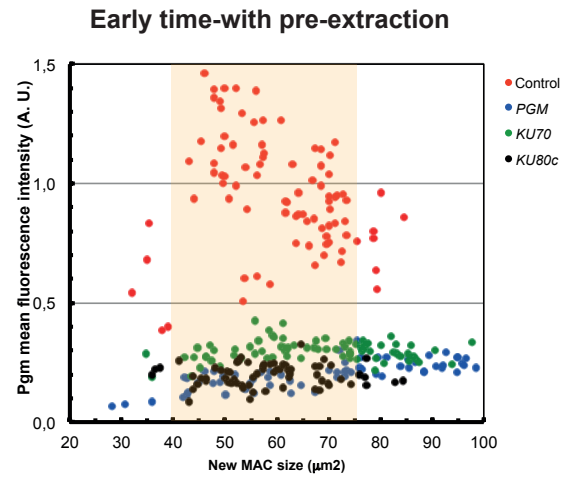**C**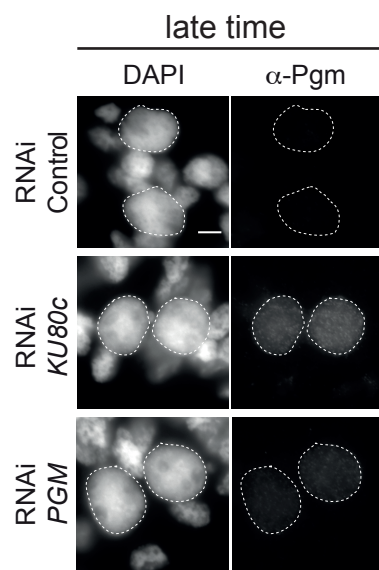**D**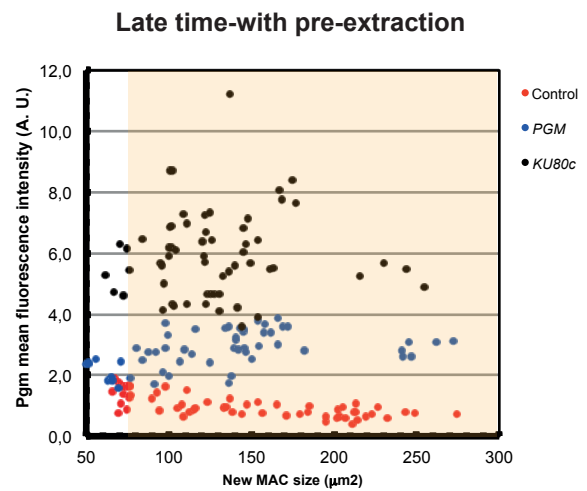**E**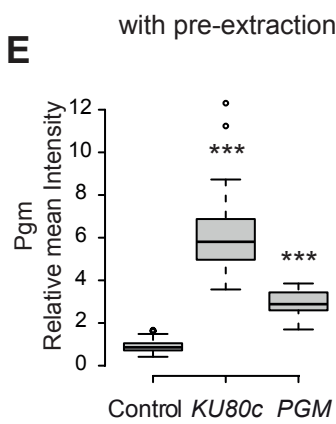

Supplement: S1 Fig — (A) At the early time point (without pre-extraction), quantification was performed for developing MAC sizes ranging between 55–110 μm2 at their maximal area section, which corresponds to the peak of Pgm signal in the control. (B) For early autogamous cells that were fixed following pre-extraction, quantification was performed for developing MAC sizes ranging between 40–75 μm2. (C) Immunostaining of Pgm in late (T30) autogamous cells subjected to control (L4440), KU80c and PGM RNAi. (D) At the late time point, all developing MACs larger than 75 μm2 were analyzed. (E) Boxplots of Pgm mean fluorescence intensity in developing MACs of late autogamous cells shown in D. From 27 to 76 developing MACs were quantified for each RNAi. (PDF) [file pgen.1008723.s001.pdf]

A

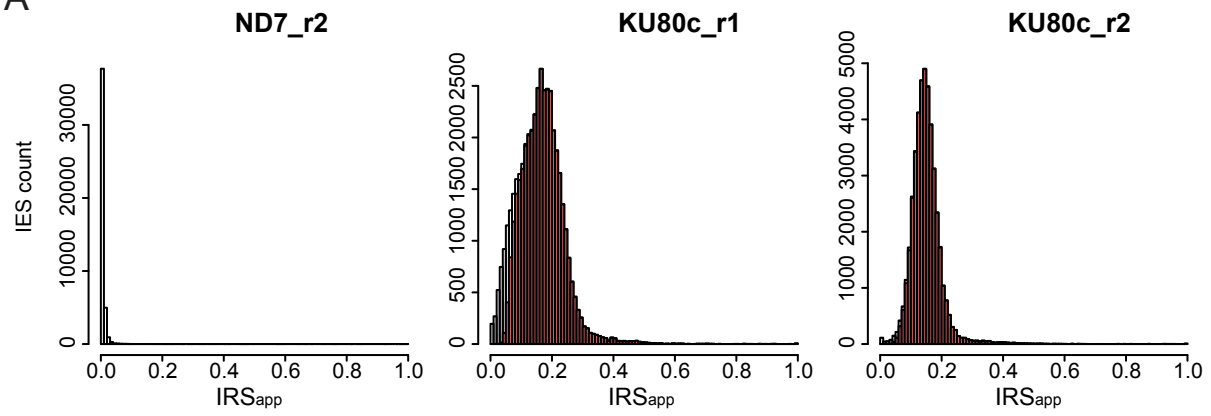

B

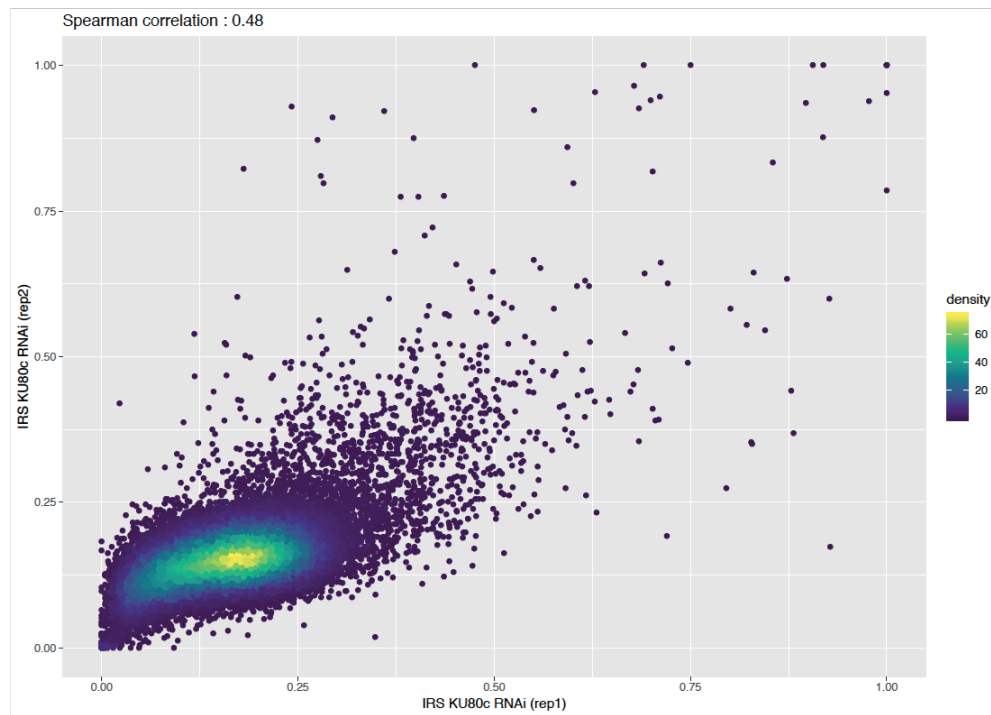

Supplement: S2 Fig — (A) Distribution of IES retention scores in total genomic DNA extracted from late autogamous cells subjected to control (ND7) or two replicates of KU80c RNAi. Data obtained with ND7_r2 and KU80c_r2 samples are also displayed in Fig 1D. Significantly retained IESs in KU80c knockdowns relative to the ND7 RNAi control are highlighted in red. (B) Spearman correlation plot of KU80c RNAi replicates. (PDF) [file pgen.1008723.s002.pdf]

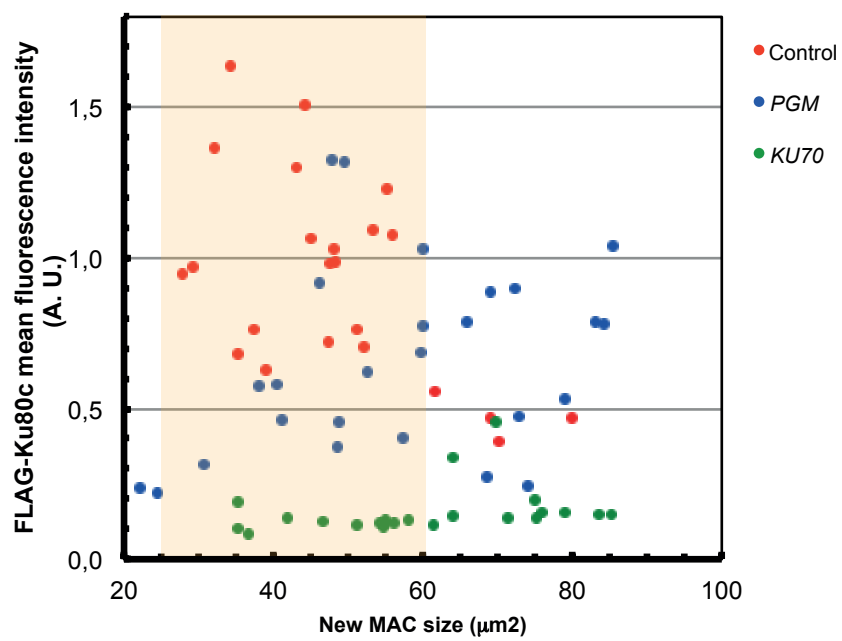

Supplement: S3 Fig — Quantification was performed for developing MAC sizes ranging between 25–60 μm2 at their maximal area section, which corresponds to the peak of the Flag signal in the control RNAi (see Fig 2). (PDF) [file pgen.1008723.s003.pdf]

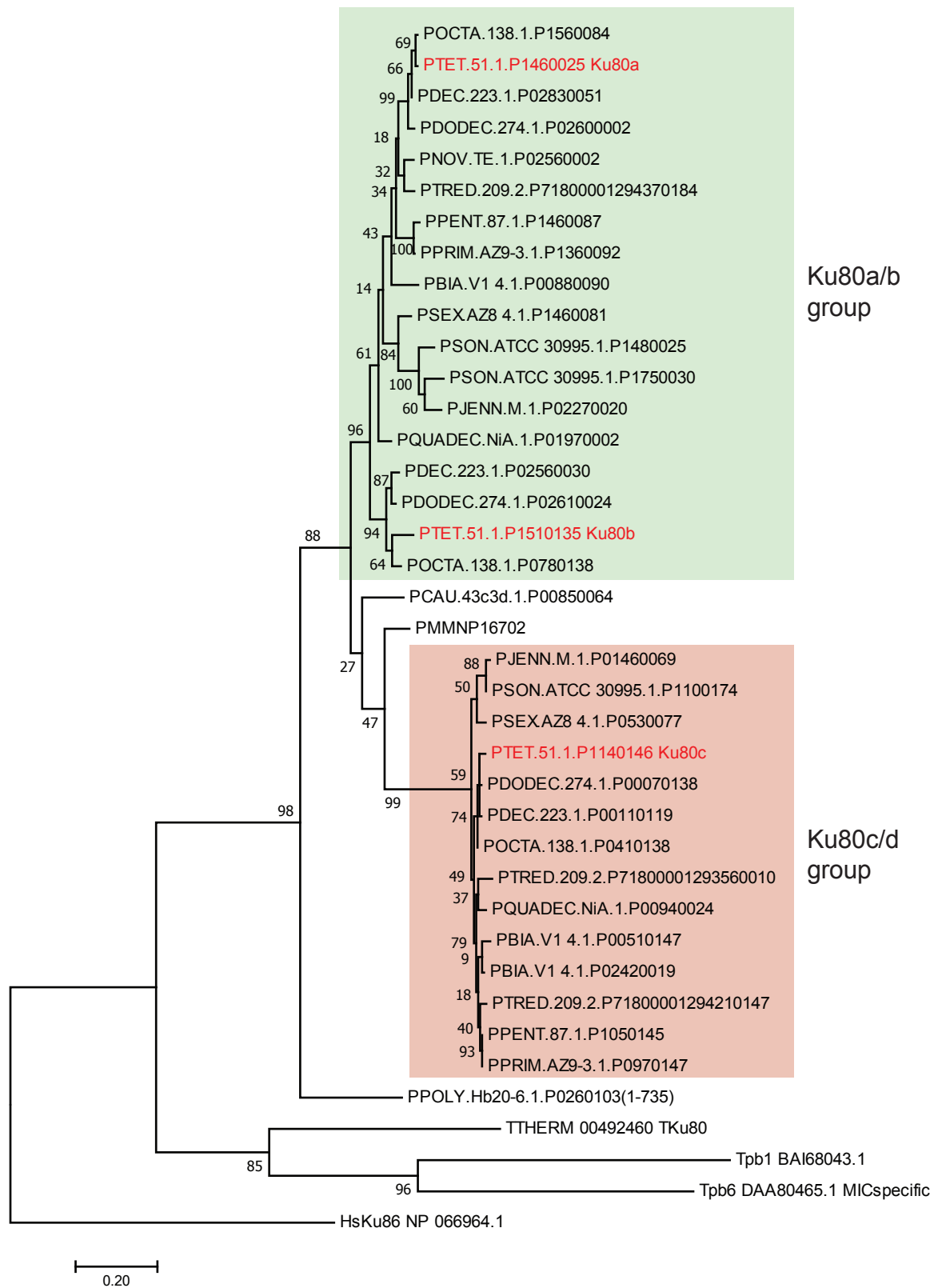

Supplement: S5 Fig — The tree includes 39 amino acid sequences of Ku80 proteins or protein domains from different Paramecium species and from Tetrahymena thermophila. Human Ku80 was used as an outgroup to root the tree. To construct the tree, the alignment of S4 Fig was edited to remove specific insertions restricted to 1 to 3 sequences only. All accession numbers are indicated. P. tetraurelia proteins are in red. The evolutionary history was inferred by using the Maximum Likelihood method based on the JTT matrix-based model [58]. The tree with the highest log likelihood (-4384.20) is shown. The percentage of trees in which the associated taxa clustered together is shown next to the branches. Initial tree(s) for the heuristic search were obtained automatically by applying Neighbor-Join and BioNJ algorithms to a matrix of pairwise distances estimated using a JTT model, and then selecting the topology with superior log likelihood value. A discrete Gamma distribution was used to model evolutionary rate differences among sites (5 categories (+G, parameter = 1.7816)). The tree is drawn to scale, with branch lengths measured in the number of substitutions per site. There were a total of 208 positions in the final dataset. Evolutionary analyses were conducted in MEGA7 [59]. The Ku80a/b and Ku80c/d groups of ohnologs from P. aurelia species are highlighted by colored boxes. (PDF) [file pgen.1008723.s005.pdf]

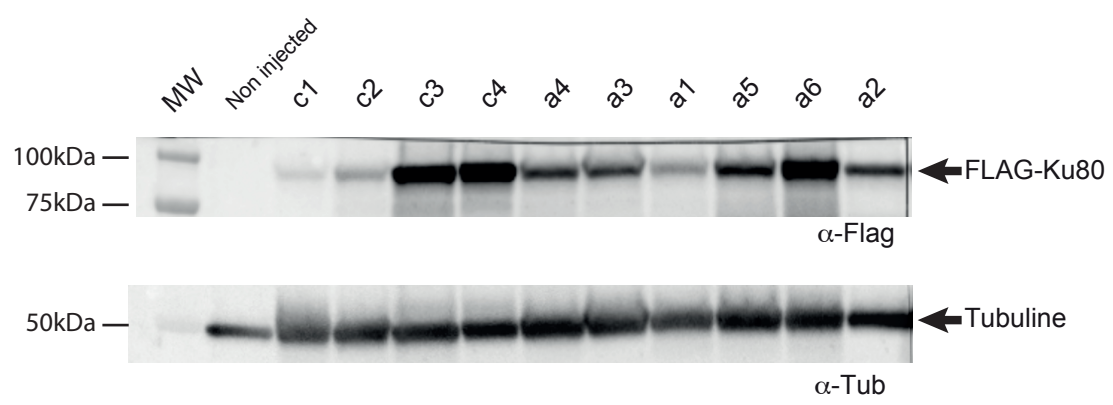

Supplement: S6 Fig — For the FLAG-KU80a and FLAG-KU80c transformants shown in Fig 3, total protein extracts were prepared at T5 during autogamy. FLAG-Ku80 proteins were revealed on western blots using α-Flag antibodies and the signal was normalized by the tubulin signal (see Fig 3B). (PDF) [file pgen.1008723.s006.pdf]

# Input

# MBP pull-down

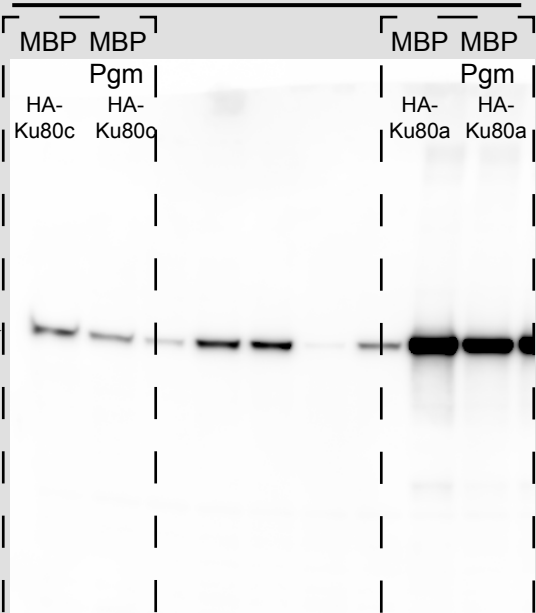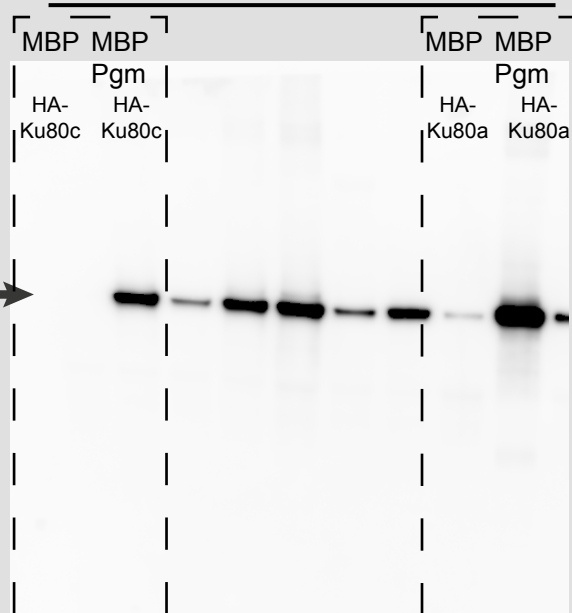

$\alpha$ -HA

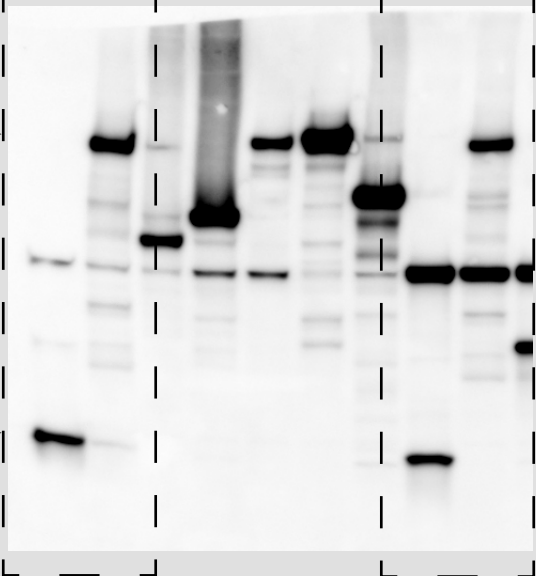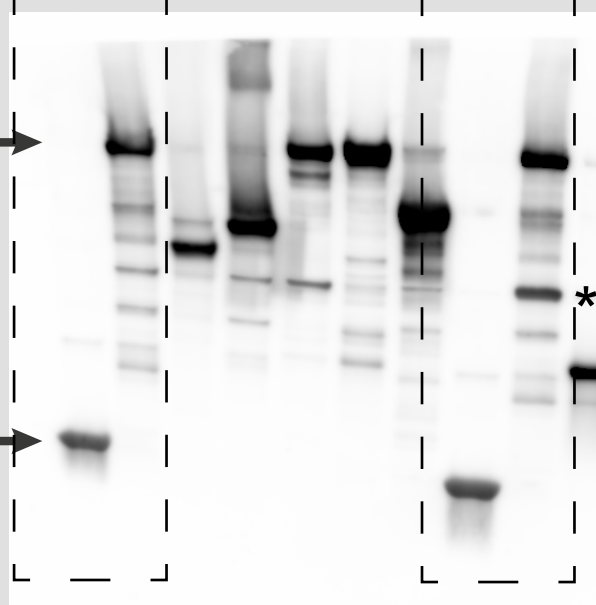

$\alpha$ -MBP

Supplement: S7 Fig — Whole pictures of the western blots shown in Fig 3D. Detection of co-immunoprecipitated HA-Ku80 was performed first using α-HA antibodies (top panels). Following membrane stripping, expression of MBP fusions in all samples was checked using α-MBP antibodies (bottom panels: the residual post-stripping HA signal is marked with an asterisk). Dotted lines delimit the lanes that were used in Fig 3D. The five central lanes of each panel are unrelated to the present study. (PDF) [file pgen.1008723.s007.pdf]

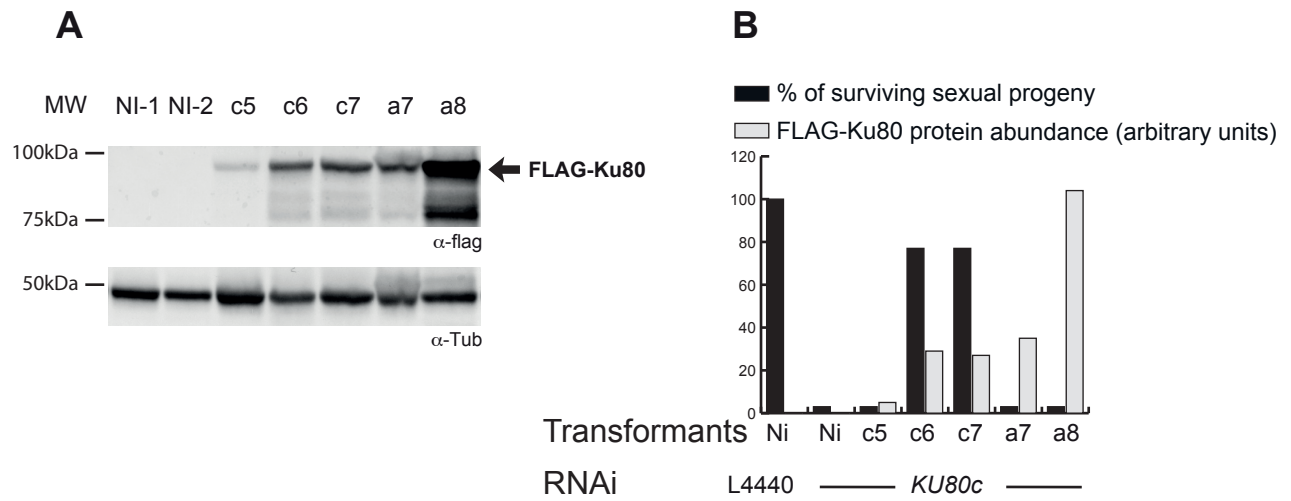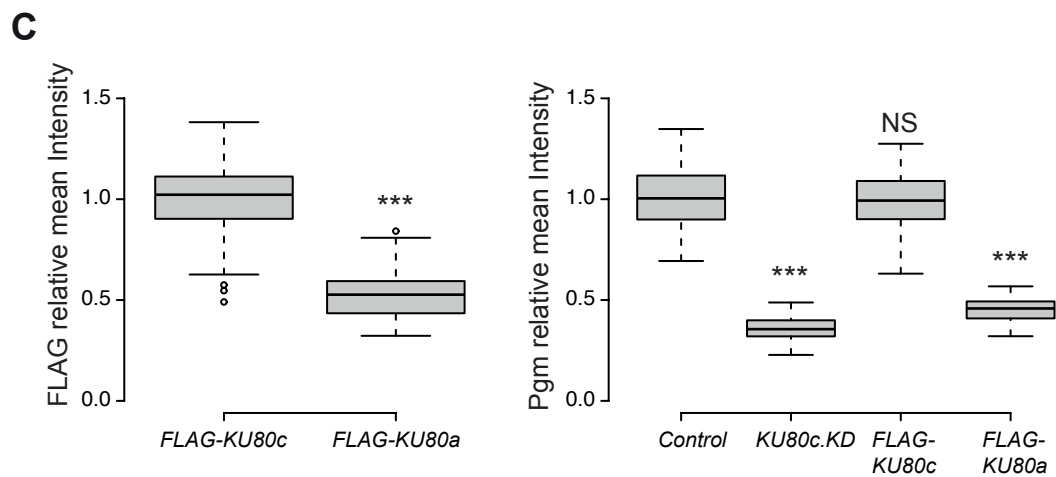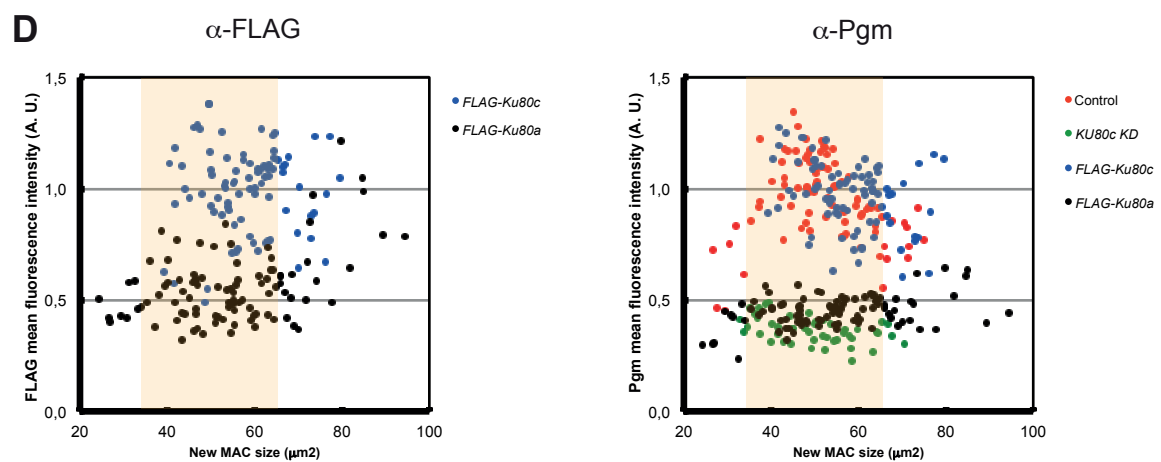

Supplement: S8 Fig — (A) Detection of FLAG-Ku80 expression in FLAG-KU80c and FLAG-KU80a transformants on western blots. Transformants c6 and a8 were picked for further quantitative immunofluorescence analysis. (B) Survival of the sexual progeny and quantification of the Flag signal relative to the Tub signal from the western blots shown in A. (C) Boxplots of FLAG-Ku80 (left panel) and Pgm (right panel) immunofluorescence intensities in developing MACs of early autogamous cells from transformants c6 and a8 subjected to KU80c RNAi (see panel D). In the right panel, the first two samples correspond to non-injected cells subjected to control RNAi (L4440: Control) or KU80c RNAi (KU80c KD). (D) Plots of FLAG-Ku80 (left panel) and Pgm (right panel) immunofluorescence intensities. Quantification for the boxplots shown in C was performed for developing MAC sizes ranging between 35–65 μm2 at their maximal area section, which corresponds to the peak of Pgm in non-injected cells subjected to control RNAi. (PDF) [file pgen.1008723.s008.pdf]

**A**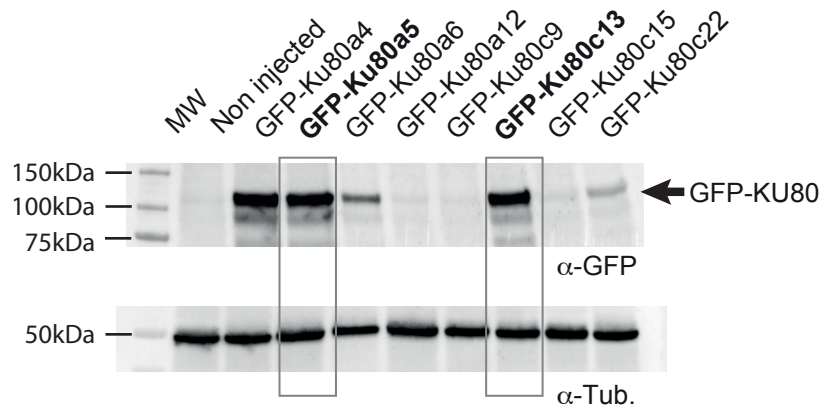**B**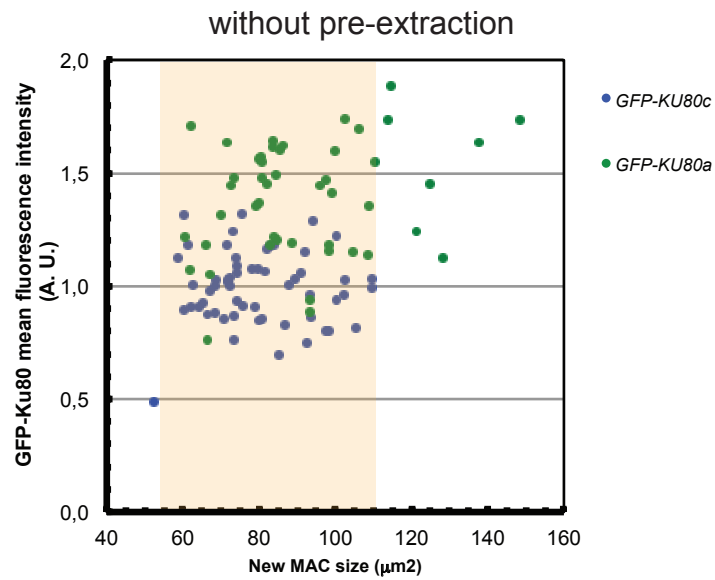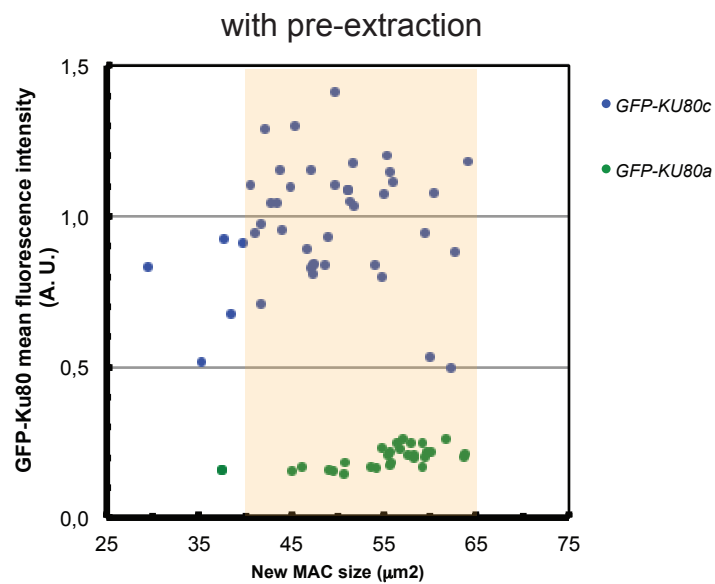

Supplement: S9 Fig — (A) Western blot analysis. Total protein extracts were prepared at T5 during autogamy. GFP-Ku80 proteins were quantified on western blots using α-GFP antibodies and the signal was normalized relative to the tubulin signal. Transformants c13 (GFP-Ku80c) and a5 (GFP-Ku80a) were picked for further analysis. (B) Plots of GFP fluorescence intensities in new MACs. Quantification for the boxplots shown in Fig 4B was performed for developing MAC sizes ranging between 55–110 μm2 or 40–65 μm2 at their maximal area section, in cells that were treated without (left panel) or with (right panel) Triton pre-extraction, respectively. These size windows correspond to the peak of GFP in GFP-Ku80c-expressing cells. The nuclear localization of GFP-Ku80c (c13) and GFP-Ku80a (a5) is analyzed in Fig 4. (PDF) [file pgen.1008723.s009.pdf]

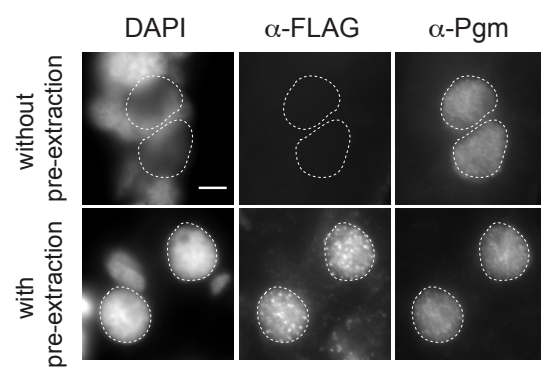

Supplement: S10 Fig — Immunostaining of FLAG-Ku80c in early autogamous cells (T5-T10) subjected to RNAi against their endogenous KU80c was performed after the cells were prepared with (bottom panel) or without (top panel) Triton pre-extraction. Scale bar is 5 μm. (PDF) [file pgen.1008723.s010.pdf]

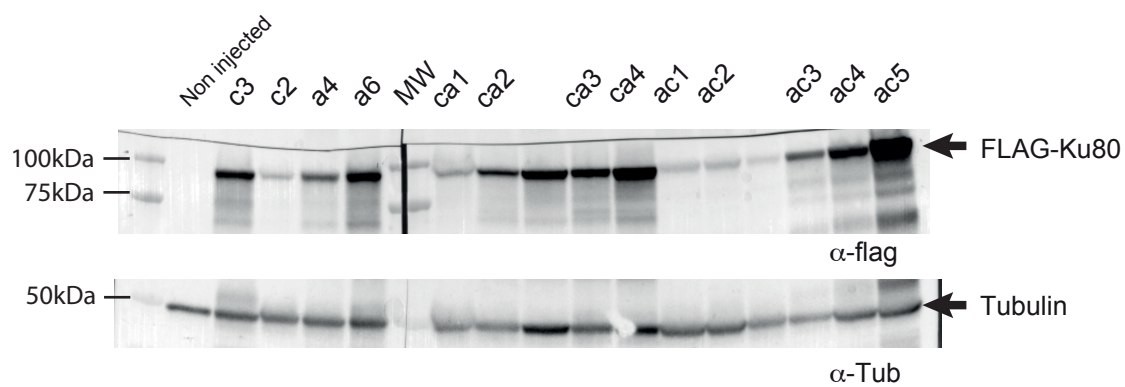

Supplement: S11 Fig — Total protein extracts were prepared at T5 during autogamy. FLAG-Ku80 proteins were quantified on western blots using α-Flag antibodies and the signal was normalized by the tubulin signal. The results of the quantification are shown in Fig 5B. (PDF) [file pgen.1008723.s011.pdf]

**A**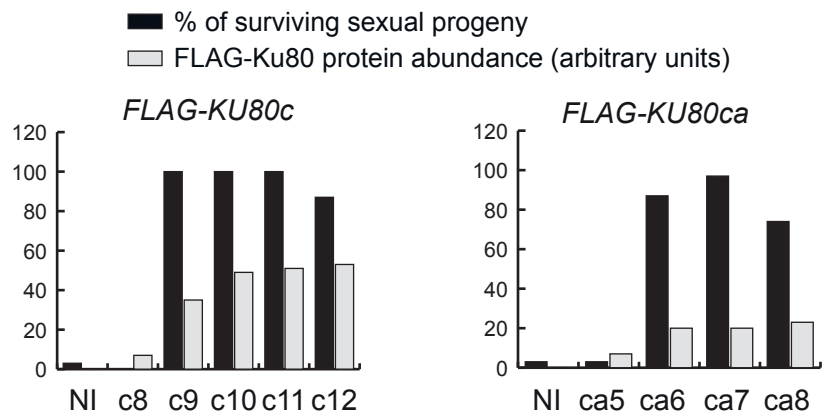**B**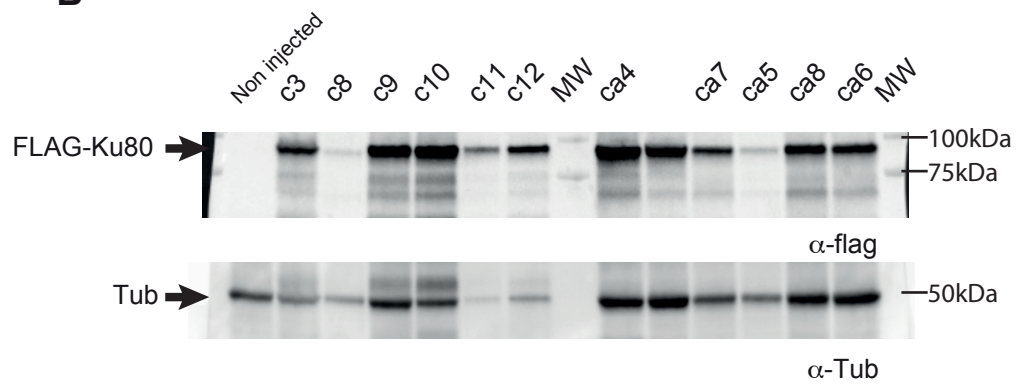

Supplement: S12 Fig — (A) Recovery of post-autogamous cells from FLAG-KU80c and FLAG-KU80ca transformants (replicates of experiments shown in Figs 3B and 5B). For each clone the percentage of surviving sexual progeny is shown in black. The relative abundance of FLAG-Ku80 (in gray) was quantified on western blots 5 hours after the beginning of autogamy and normalized by the tubulin signal. (B) Western blot analysis of FLAG-Ku80 expression in early autogamous FLAG-KU80c and FLAG-KU80ca transformants subjected to KU80c RNAi. The ca8 transformant was used for the genome-wide analyses shown in Fig 6. (PDF) [file pgen.1008723.s012.pdf]

### c2-transformant

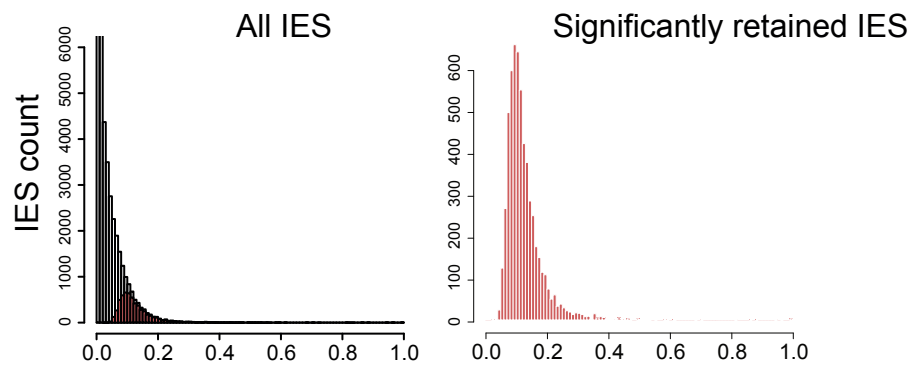

### ca3-transformant

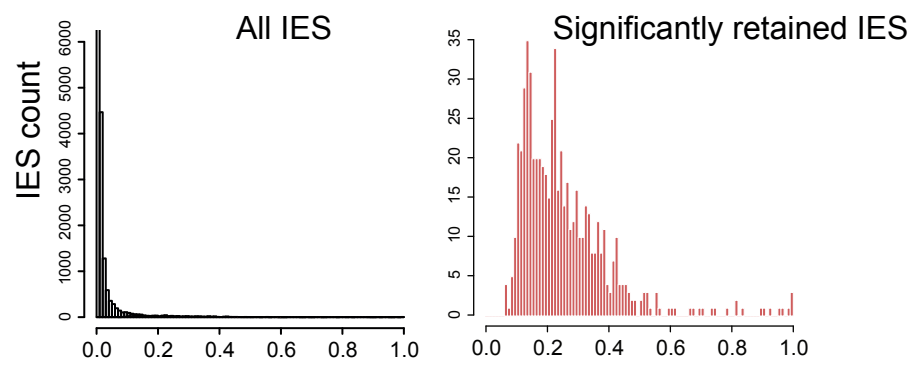

### ca8-transformant

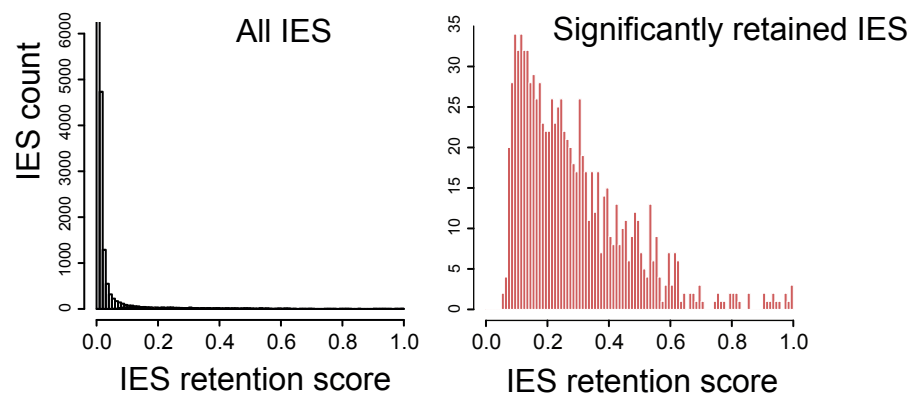

Supplement: S13 Fig — Left panels: Retention scores were plotted for all IESs in the genome, with significantly retained IESs (relative to the control ND7 RNAi) highlighted in red. Right panels: Distribution of significantly retained IESs in each experiment shown on the left. (PDF) [file pgen.1008723.s013.pdf]

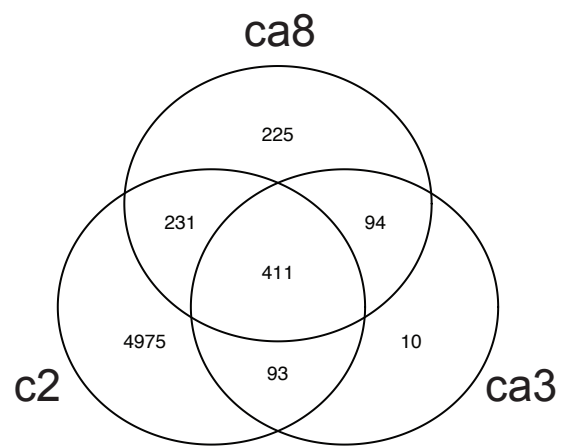

Supplement: S14 Fig — The sets of significantly retained IESs in transformants c2, ca3 and ca8 subjected to KU80c RNAi are displayed in S13 Fig. (PDF) [file pgen.1008723.s014.pdf]

**A**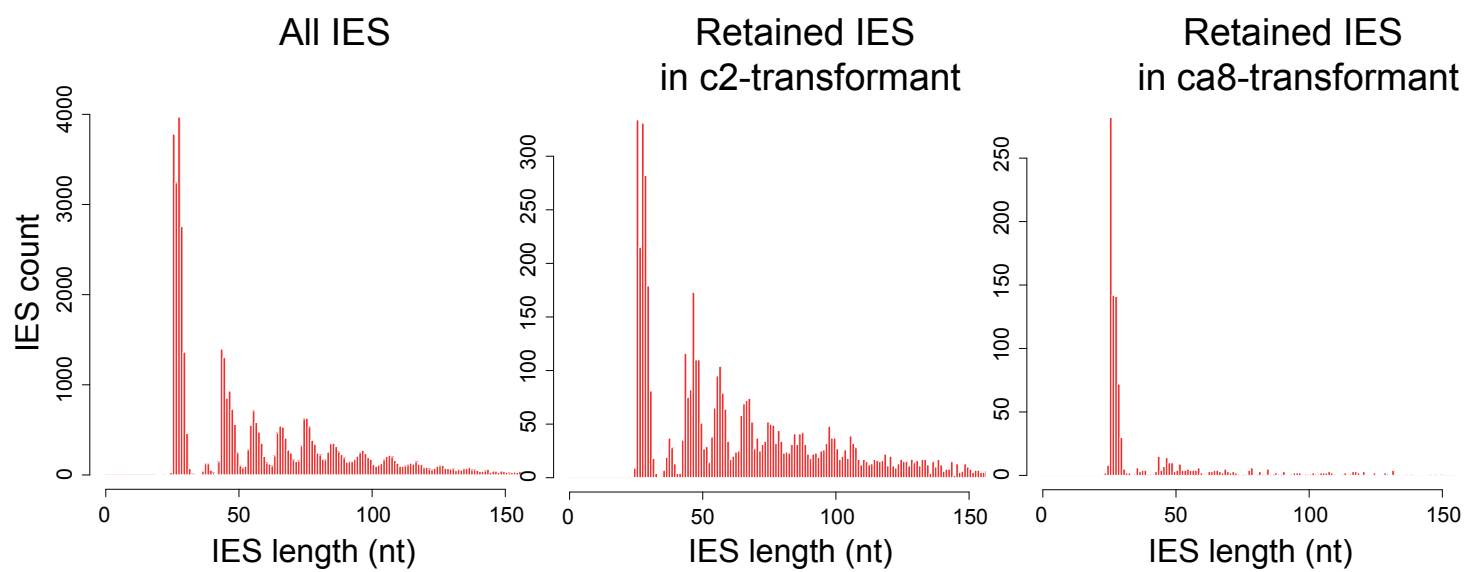**B**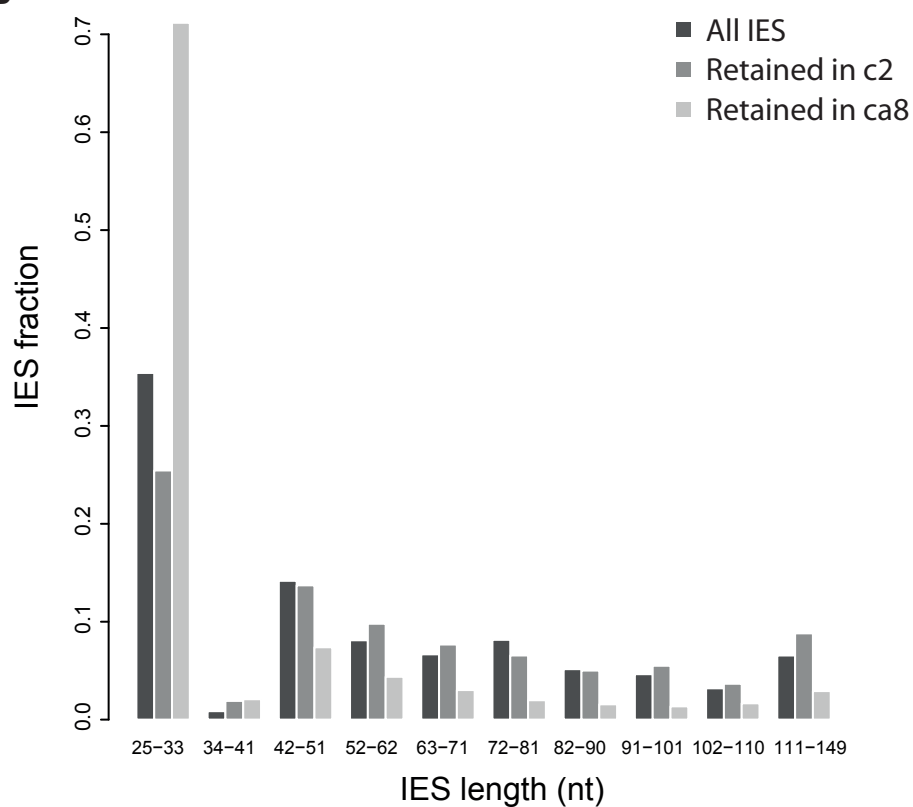

Supplement: S15 Fig — (A) IES length distributions for all IESs (left) and significantly retained IESs in c2 (middle) and ca8 (right) transformants subjected to KU80c RNAi. (B) Relative distribution of IES lengths for all IESs (black) and significantly retained IESs in c2 (dark gray) and ca8 (light gray). Only IESs shorter than 150 bp were taken into account to draw the graphs. (PDF) [file pgen.1008723.s015.pdf]

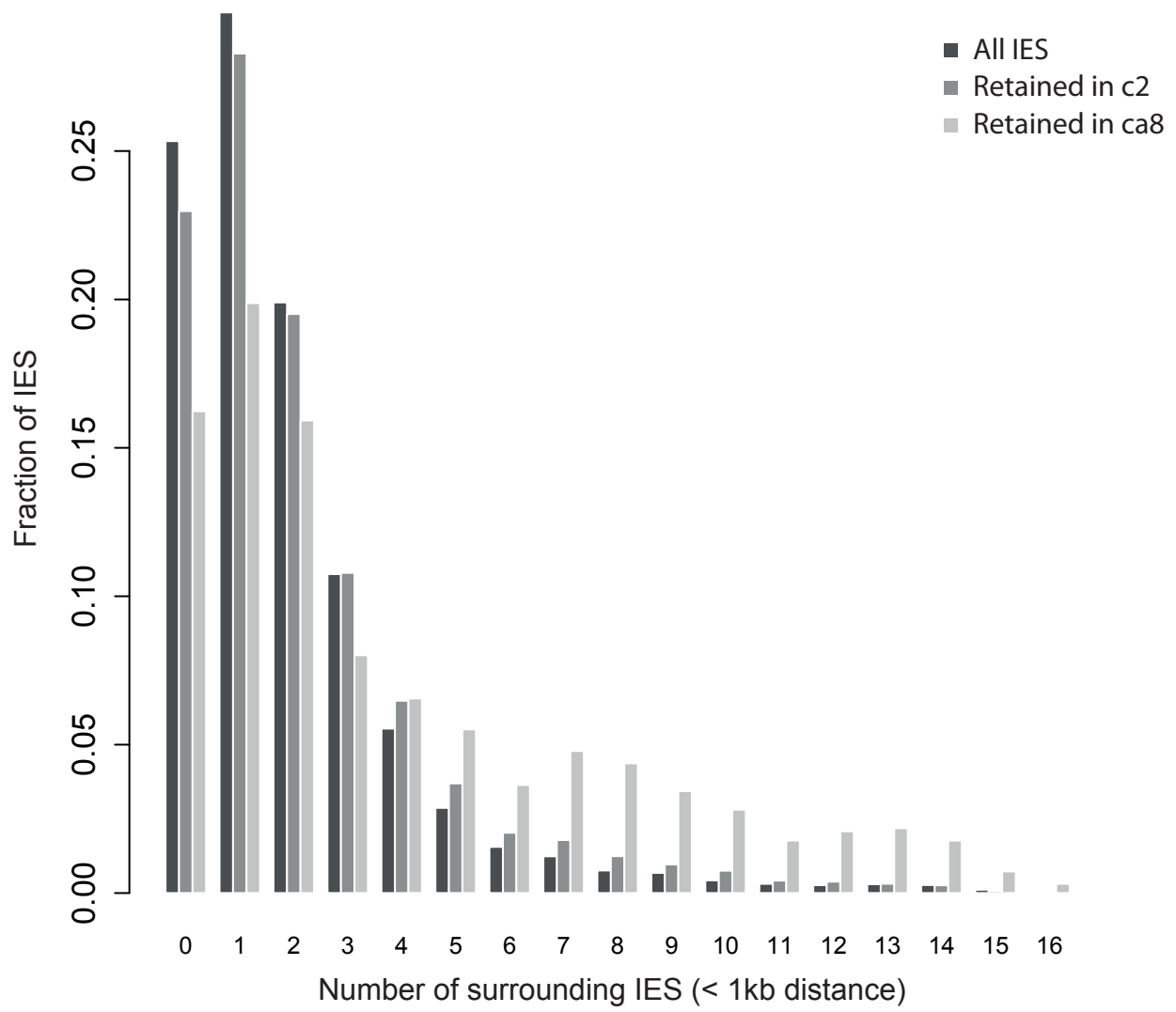

Supplement: S16 Fig — For each condition (ca8 or c2 transformants subjected to KU80c RNAi), retained IESs were classified in density subsets based on the number of surrounding IESs located at a <1kb distance, and the fraction represented by each subset relative to all retained IESs was calculated. For all IESs, the total fraction of IESs in each density subset is represented (black bars). (PDF) [file pgen.1008723.s016.pdf]
