# Supplementary material for: Functional diversification of Paramecium Ku80 paralogs safeguards genome integrity during precise programmed DNA elimination
Source: PLoS Genet. 2020 Apr 16;16(4):e1008723. doi: 10.1371/journal.pgen.1008723 (PMC7161955; doi:10.1371/journal.pgen.1008723)
Supplement: S4 Fig — The analysis includes 39 amino acid sequences of Ku80 proteins or protein domains from different Paramecium species, Tetrahymena thermophila and Homo sapiens. Full-length sequences were used for the alignment, except for P. polycaryum Ku80 (PPOLY.Hb20-6.1.P0260103: residues 1–735) and the Ku80 domains of T. thermophila Tpb1 and Tpb6 (residues 1–704 and 1–709, respectively). Amino acid sequences were aligned using MUSCLE (http://www.ebi.ac.uk/Tools/msa/muscle/). Accession numbers of P. tetraurelia proteins: Ku80a (PTET.51.1.P1460025), Ku80b (PTET.51.1.P1510135), Ku80c (PTET.51.1.P1140146). Complete accession numbers can be found in S5 Fig. Note that P. novaurelia also encodes Ku80c/d proteins, which were not included in the alignment because their full sequence could not be deduced from the current assembly of the somatic genome. (PDF) [file pgen.1008723.s004.pdf]

```
Tpb1_BAI68043.1      1 MKKKFIQNDCILIV-LNQQASQRMKAQF-------------IESVSLILKNLMFN-QQQS
HsKu86_NP_06696      1 --MVRSGNKAAVVLCMDVGFTMSNSIPG-----IESPFEQAKKVITMFVQRQVFAENKDE
Tpb6_DAA80465.1      1 ----MYRREYFIVL-LDSGFIKHQQMPEEEILDQNKIVDQATNMIQLLLFDKIRNQQENM
TTHERM_00492460      1 ----MAGKEATIIL-LDMGSSM-QQYLGDRGTNGQKRIEIAVNCIKLLLQQKMFNTKTHE
PPOLY.Hb20-6.1.      1 ----MAGKEATLVL-LDVGKSMQQQYE--GSKNKMKRLDISIDCINLMLENKLFNFKNHE
PTRED.209.2.P71      1 -----------------------------------------------MIQQKIFNYKNHE
PJENN.M.1.P0146      1 ----MSGKEASLIL-LDVGASMYGEYQM-GGGKKLSRLELAVDCLGLMIQQKIFNYKNHE
PSON.ATCC_30995      1 ----MSGKEASLIL-LDVGASMYGEYQM-GGGKKLSRLELAVDCLGLMIQQKIFNYKNHE
PSEX.AZ8_4.1.P0      1 ----MSGKEATLIL-LDVGASMYSEYQQ-GGSKKLTRLELAVDCLGLMIQQKIFNYKNHE
PQUADEC.NiA.1.P      1 ----MSGREATLIL-LDVGASMYGQYQQ-GGNRKMSRLELAVDCLGLMIQQKIFNYKNHE
PBIA.V1_4.1.P00      1 ----MSGKEATLIL-LDVGASMYGQYQQ-GGSRKMSRLELAVDCLGLMIQQKIFNYKNHE
PTRED.209.2.P71      1 ----MSGKEATLIL-LDVGASMYGQYLQ-GGNRKMSRLELAVDCLGLMIQQKIFNYKNHE
PTET.51.1.P1140      1 ----MSGKEATLIL-LDVGASMYGQYQQ-GGSKKLSRLELAVDCLGLMIQQKIFNYKNHE
POCTA.138.1.P04      1 ----MSGKEATLIL-LDVGASMYSQYQQ-GGSKKLSRLELAVDCLGLMIQQKIFNYKNHE
PDEC.223.1.P001      1 ----MSGKEATLIL-LDVGASMYSQYQQ-GGSKKLSRLELAVDCLGLMIQQKIFNYKNHE
PDODEC.274.1.P0      1 ----MSGKEATLIL-LDVGASMYGQYQQ-GGSKKLSRLELAVDCLGLMIQQKIFNYKNHE
PBIA.V1_4.1.P02      1 ----MSSKEATLIL-LDVGASMYGQYQQ-GGNKKLSRLELAVDCIGLMIQQKIFNYKNHE
PPENT.87.1.P105      1 ----MSGREATLIL-LDVGASMYSQYQQ-GGNKKLSRLELAVDCLGLMIQQKIFNYKNHE
PPRIM.AZ9-3.1.P      1 ----MSGREATLIL-LDVGASMYSQYQQ-GGNKKLSRLELAVDCLGLMIQQKIFNYKNHE
PDEC.223.1.P025      1 ----MAGKEATLVL-LDVGASMYEPYKQ-AQGKNITRLELAVDCIGMMIQQKIFNYKNHE
PDODEC.274.1.P0      1 ----MAGKEATLVL-LDVGASMYEPYKQ-AQGKNITRLELAVDCIGMMIQQKIFNYKNHE
PTET.51.1.P1510      1 ----MAGKEATLVL-LDVGASMYEPYKQ-AQGKNITRLELAVDCIGMMIQQKIFNYKNHE
POCTA.138.1.P07      1 ----MAGKEATLVL-LDVGASMYEPYKQ-AQGKNITRLELAVDCIGMMIQQKIFNYKNHE
PSEX.AZ8_4.1.P1      1 ----MAGKEATLVL-LDVGASMYEPYKQ-GQGKKITRLELAVDCLGMMIQQKIFNYKNHE
PPENT.87.1.P146      1 ----MAGKEATLVL-LDVGASMYQPYKQ-AQGKKITRLELAVDCLGMMIQQKIFNYKNHE
PPRIM.AZ9-3.1.P      1 ----MAGKEATLVL-LDVGASMYEPYKQ-AQGKKITRLELAVDCLGMMIQQKIFNYKNHE
PBIA.V1_4.1.P00      1 ----MAGKEATLVL-LDVGASMYEPYKQ-AQGKKITRLELAVDCIGMMIQQKIFNYKNHE
PQUADEC.NiA.1.P      1 ----MAGKEATLVL-LDVGASMYQAYKQ-AQGKKITRLELAVDCIGMMIQQKIFNYKNHE
POCTA.138.1.P15      1 ----MAGKEATLVL-LDVGASMYEPYKQ-AQGKKITRLELAVDCIGMMIQQKIFNYKNHE
PTET.51.1.P1460      1 ----MAGKEATLVL-LDVGASMYEPYKQ-AQGKKITRLELAVDCIGMMIQQKIFNYKNHE
PDODEC.274.1.P0      1 ----MAGKEATLVL-LDVGASMYEPYKQ-AQGKKITRLELAVDCIGMMIQQKIFNYKNHE
PDEC.223.1.P028      1 ----MAGKEATLVL-LDVGASMYEPYKQ-AQGKKITRLELAVDCIGMMIQQKIFNYKNHE
PNOV.TE.1.P0256      1 ----MAGKEATLVL-LDVGASMYEPYKQ-AQGKKITRLELAVDCIGMMIQQKIFNYKNHE
PTRED.209.2.P71      1 ----MAGKEATLVL-LDVGASMYQAYKQ-AQGKKITRLELAVDCIGMMIQQKIFNYKNHE
PSON.ATCC_30995      1 ----MAGKEATLVL-LDVGASMYEPYKQ-VQGKEIRRIELAIDCIGMMIQQKIFNYKNHE
PSON.ATCC_30995      1 ----MAGKEATLVL-LDVGSSMYEKYKQGGQGKEIRRLELAIDCIGMMIQQKIFNYKNHE
PJENN.M.1.P0227      1 ----MAGKEATLVL-LDVGASMYEQYKQ-VQGKQITRLELAIDCIGMMIQQKIFNYKNHE
PCAU.43c3d.1.P0      1 ----MAGKEATLVL-LDVGASMYTPYQE-AQGKKMTRLELAVDCITLMLQQKIFNYKNHE
PMMNP16702           1 ----MAGKEATMIL-LDVGASMYQPYQQ-AQGKQLTRLELAVDCINLMIQQKIFNYKNHE
```

```
Tpb1_BAI68043.1      46 VHQILFIPDQYEK------HFSQKTSE-------PNFTQVLFKDIRNFQD--KAKEFIENV
HsKu86_NP_06696      54 IALVLFGTDGTDNPLSGGDQY-QNITVHRHLMLPDFD--LLEDIESKIQ-----PGSQQA
Tpb6_DAA80465.1      56 FQIQLFKQQNNDE---GEIFSFLESKQ-------PIIQ--DIKNINNLKKSLQQDEQSIQK
TTHERM_00492460      55 VGLILFGLKDEGD---DKIMYIRGIGK-------PDID--FLKNVQDLKD--YQSDECEGG
PPOLY.Hb20-6.1.      54 VGLILFGAADAPD---GNTIYAIDISK-------PNFE--FIRSVSRLRN--HKNFDVQGG
PTRED.209.2.P71      14 IGLILFGTEEAPD---GKTLYIQDLSI-------PDLD--FFRNLNDLPN--HDVGQQVGG
PJENN.M.1.P0146      55 VGLILFGTEEAPD---GKTLYIQDLSI-------PDLD--FFRNVSELPN--HDVGLQVGG
PSON.ATCC_30995      55 VGLILFGTEEAPD---GKTLYIQDLSI-------PDLD--FFRNVSELPN--HDVGLQVGG
PSEX.AZ8_4.1.P0      55 IGLILFGTEEAPD---GKTLYIQDLSI-------PDLD--FFRNVSELPN--HDVGLQVGG
PQUADEC.NiA.1.P      55 VGLILFGTEEAPD---GKTLYIQDLSI-------PDLD--FFRNINDLPN--HDVGQQVGG
PBIA.V1_4.1.P00      55 IGLILFGTEEAPD---GKTLYIQDLSI-------PDLD--FFRNISDLPN--HDVGQQVGG
PTRED.209.2.P71      55 IGLILFGTEEAPD---GKTLYIQDLSI-------PDLD--FFRNISDLPN--HDVGQQVGG
PTET.51.1.P1140      55 VGLILFGTEEAPD---GKTLYIQDLSI-------PDLD--FFRNISDLPN--HDVGQQVGG
POCTA.138.1.P04      55 VGLILFGTEEAPD---GKTLYIQDLSI-------PDLD--FFRNISDLPN--HDVGQQVGG
PDEC.223.1.P001      55 VGLILFGTEEAPD---GKTLYIQDLSI-------PDLD--FFRNISDLPN--HDVGQQVGG
PDODEC.274.1.P0      55 VGLILFGTEEAPD---GKTLYIQDLSI-------PDLD--FFRNIGDLPN--HDVGQQVGG
PBIA.V1_4.1.P02      55 IGLILFGTEEAPD---GKTLYIQDLSI-------PDLD--FFRNISDLPN--HDVGQQVGG
PPENT.87.1.P105      55 VGLILFGTEEAPD---GKTLYIQDLSI-------PDLD--FFRNLSDLPN--HDVAQQVGG
PPRIM.AZ9-3.1.P      55 VGLILFGTEEAPD---GKTLYIQDLSI-------PDLD--FFRNLSDLPN--HDVAQQVGG
PDEC.223.1.P025      55 VGLVLFGTEEAED---GNTFYIQVMST-------PDLE--FYRNLTELPN--HDIPQIKGG
PDODEC.274.1.P0      55 VGLVLFGTEEAED---GNTFYIQVMSP-------PDLE--FYRNLTELPN--HDIPQIKGG
PTET.51.1.P1510      55 VGLVLFGTEEAED---GNTFYIQVMSP-------PDLE--FYRNLTELPN--HDVPKIKGG
POCTA.138.1.P07      55 VGLVLFGTEEAED---GNTFYIQIMST-------PDLE--FYRNLTELPN--HDVPQKQGG
PSEX.AZ8_4.1.P1      55 VGLVLFGTEDAED---GNTFYIQMLSP-------PDLE--FYRNVTELPT--HDRPQKQGG
PPENT.87.1.P146      55 VGLVLFGTEEAED---GNTFYIQTLST-------PDLE--FYRNVTELIN--HDIPKIQGG
PPRIM.AZ9-3.1.P      55 VGLVLFGTEEAED---GNTFYIQTLST-------PDLE--FYRNVTELIN--HDIPKIQGG
PBIA.V1_4.1.P00      55 VGLVLFGTEEAED---GNTFYIQTLSS-------PDLE--FYRNLTELPN--HDLPKIIGG
PQUADEC.NiA.1.P      55 VGLVLFGTEEAED---GNTIYIQLLSQ-------PNLE--FYRNVTELTN--HDIPQIAGG
POCTA.138.1.P15      55 VGLVLFGTEDAED---GNTFYIQTLSS-------PDLE--FYRNLTELPD--HDKPKIIGG
PTET.51.1.P1460      55 VGLVLFGTEDAED---GNTFYIQTLSS-------PDLE--FYRNLTELPN--HDIPKIIGG
PDODEC.274.1.P0      55 VGLVLFGTEEAED---GNTFYIQTLSS-------PDLE--FYRNLTELPN--HDIPKITGG
PDEC.223.1.P028      55 VGLVLFGTEDAED---GNTFYIQTLSS-------PDLE--FYRNLTELPN--HDIPKITGG
PNOV.TE.1.P0256      55 VGLVLFGTEEAED---GNTFYIQQLSQ-------PNLE--FYRNVTELTN--HDLPQINGG
PTRED.209.2.P71      55 VGLVLFGTEEAED---GNTIYIQPLSQ-------PNLE--FYRNVTELTN--HDMPKMNGG
PSON.ATCC_30995      55 VGLVLFGTEDAED---GNTFYIQTLSP-------PDLE--FYRNVTELPK--HDIPKIKGG
PSON.ATCC_30995      56 VGLVLFGAEDAED---GNTFYIQQISA-------PDFE--FYRNVTELVK--HDIPKIRGG
PJENN.M.1.P0227      55 VGLVLFGTEDAED---GNTFYIQQLST-------PDLE--FYRNVTELSK--HDIPKIRGG
PCAU.43c3d.1.P0      55 VGLILFGTEEAQD---GNTLYIQEINT-------PDLE--FFRNVSELPT--HTASQIQGG
PMMNP16702           55 IGLILFGTEEASD---GKTLYIQEIQQ-------PDLE--FFRNVSELPT--HDVPQLEGG
```

```
Tpb1_BAI68043.1     92 DVQEKISFIEVLYNSRNELTAFAKYKQLNKKLFFFMGETESFFFGQSKMFQLQEFIMHLK
HsKu86_NP_06696    106 DF------LDALIVSMDVIQHETIGKKFEKRHIEIFTDLSSR-FSKSQLDIIIHSL---K
Tpb6_DAA80465.1    105 TI------FVPLIKLIEQKKQNTQFMLRQKKIYYLTTGQSQYKPEQRQINQLKDFI---K
TTHERM_00492460    102 DI------FEAIEQTIDVIHDYVKEKKYEKKIQLMTAGFGKTSYKEKQIMDLIEKA---R
PPOLY.Hb20-6.1.    101 DI------FDALDKAVHTLDEYVNTKKIEKKIFILTNGSGSTDYSEKQIQKLIRMI---V
PTRED.209.2.P71     61 DI------FDALDKAVHTLDDHAKTKKMEKKIFILTAGCGQTDYSEKQITKLIRMI---E
PJENN.M.1.P0146    102 DI------FDALDKAVHVLDDHTKTKRMEKKIYILTAGCGQTDYSEKQITKLIKMI---E
PSON.ATCC_30995    102 DI------FDALDKAVHVLDDHTKTKRMEKKIYILTAGCGQTDYSEKQITKLIKMI---E
PSEX.AZ8_4.1.P0    102 DI------FDALDKAVHALDDHAKTKKVEKKIFILTAGCGQTDYSEKQITKLIKMI---E
PQUADEC.NiA.1.P    102 DI------FDALDKAVHALDDHVKTKKMEKKIFILTAGCGQTDYSEKQITKLIKMI---E
PBIA.V1_4.1.P00    102 DI------FDALDKAVHALDDHAKTKRMEKKIFILTAGCGQTDYSEKQITKLIKMI---E
PTRED.209.2.P71    102 DI------FDALDKAVHALDDHAKTRKMEKKIFILTAGCGQTDYSEKQITKLIKMI---E
PTET.51.1.P1140    102 DI------FDALDKAVHALDDHAKTKKMEKKIFILTAGCGQTDYSEKQITKLIKMI---E
POCTA.138.1.P04    102 DI------FDALDKAVHALDDHAKTKKMEKKIFILTAGCGQTDYSEKQITKLIKMI---E
PDEC.223.1.P001    102 DI------FDALDKAVHALDDHAKTKKMEKKIFVLTAGCGQTDYSEKQITKLIKMI---E
PDODEC.274.1.P0    102 DI------FDALDKAVHALDDHAKTKKMEKKIFVLTAGCGQTDYSEKQITKLIKMI---E
PBIA.V1_4.1.P02    102 DI------FDALDKAVHALDDHAKTKKMEKKIFILTAGCGQTDYSEKQITKLIKMI---E
PPENT.87.1.P105    102 DI------FDALDKAVHALDDHAKTKKMEKKIFILTAGCGQTDYSEKQITKLIKMI---E
PPRIM.AZ9-3.1.P    102 DI------FDALDKAVHALDDHAKTKKMEKKIFILTAGCGQTDYSEKQITKLIKMI---E
PDEC.223.1.P025    102 DI------FDALDKAVSTLDEHVKAKKIDKKIFVLTAGFGQTQYNEKKIGKLIKMI---E
PDODEC.274.1.P0    102 DI------FDALDKAVSTLDEHVKTKKIDKKIFVLTAGFGQTQYNEKKIGKLIKMI---E
PTET.51.1.P1510    102 DI------FDALDKAVSTLDEYVKAKKMEKKIFVLTAGFGQTDYNEKKIGKLIKMI---E
POCTA.138.1.P07    102 DI------FDALDKAVSTLDEHVKTKKMEKKIFVLTAGYGQTEYNEKKIGKLIKMI---E
PSEX.AZ8_4.1.P1    102 DI------FDALDKAICTLDGHVKTKKMDKKIFVLTSGFGQTEYNEKKIAKLIKMI---E
PPENT.87.1.P146    102 DI------FDALDKSVSTLDQHVKTKKMDKKIFVLTAGFGQTDYNEKKIAKLVKMI---E
PPRIM.AZ9-3.1.P    102 DI------FDALDKSVSTLDEYVKTKKIDKKIFVLTAGFGQTDYNEKKIAKLVKMI---E
PBIA.V1_4.1.P00    102 DI------FDALDKSVSSLDEHVKTKKMEKKIFVLTAGYGQTEYNEKKIAKLIKMI---E
PQUADEC.NiA.1.P    102 DI------FDALDKAVSTLDEHVKAKKIDKKIFVLTAGYGQTEYNEKKIAKLIKMI---E
POCTA.138.1.P15    102 DI------FDALDKSVSTLDEYVKTKKMDKKIFVLTAGFGQTEYNEKKIAKLIKMI---E
PTET.51.1.P1460    102 DI------FDALDKSVSTLDEYVKTKKMDKKIFVLTAGFGQTEYNEKKIAKLIKMI---E
PDODEC.274.1.P0    102 DI------FDALDKSVSTLDEHVKTKKMDKKIFVLTAGFGQTEYNEKKIAKLIKMI---E
PDEC.223.1.P028    102 DI------FDALDKSVSTLDEYVKTKKMDKKIFVLTAGFGQTEYNEKKIAKLIKMI---E
PNOV.TE.1.P0256    102 DI------FDALDKSVSTLDEHVKTKKMDKKIFVLTAGYGQTEYNEKKIAKLIKMI---E
PTRED.209.2.P71    102 DI------FDALDKAVSTLDEHVKTKKMDKKIFVLTAGFGQTEYNEKKIAKLIKMI---E
PSON.ATCC_30995    102 DI------FDALDKSVSTLDDHVKTKKMDKKIFVLTAGFGQTEYNEKKIGKLIKMI---E
PSON.ATCC_30995    103 DI------FDALDKSVSTLDDHVKTKKMDKKIFVLTAGFGQTEYNEKMIAKLIKMI---Q
PJENN.M.1.P0227    102 DI------FDALDKSVSTLDEHVKTKKMDKKIFVLTAGFGQTEYNEKKIAKLIKMI---E
PCAU.43c3d.1.P0    102 DI------FDALDKGIHTLDDYVKQRKIEKKIFILTAGCGQTEYSEKQLTKLIRMI---E
PMMNP16702         102 DI------FDALDKAVHTLDDYVKTKKIDKKIFILTSGYGQTDYSEKHLTKLIKMI---E
```

```
Tpb1_BAI68043.1   152 RDQIKITLIGEEIMKNLREDSVTDWKDNQNIEFL-----IKLKNQISKKNIQ----FL--
HsKu86_NP_06696   156 KCDISLQFFLPFSLGKEDGS-GDRGDGPFRLGGHGPSFPLKGITEQQKEGLEIVKMVM-I
Tpb6_DAA80465.1   156 QFNIKINIILFDYLND-----LNDRNNSNNYE--------------------NNKHAFLDL
TTHERM_00492460   153 KVQTKINVIGFDFLKKYNPE-ESNTDVLKKDDAAQN----------TRQNLNQKLFSV-V
PPOLY.Hb20-6.1.   152 KVDVKINFIALDFMNQYDPD-MDDPSKPDVLEVLTHRM-MTAETESREQLINARHIFL-M
PTRED.209.2.P71   112 KIDVKINFIALDFMNDYNSY-MDDPDKPEEFEALNNRM-LTASYQCQEQSINSRYLFV-M
PJENN.M.1.P0146   153 KVDVKINFIALDFMNDYNGE-MDDPDKPEEYEALNNRM-LTASYQCQEQSINSRYVFL-M
PSON.ATCC_30995   153 KVDVKINFIALDFMNDYNGD-MDDPDKPEEYEALNNRM-LTASYQCQEQSINSRYVFL-M
PSEX.AZ8_4.1.P0   153 KVDVKINFIALDFMNDYNGD-MDDPDKPEEFEALNNRM-LTASYQCQEQSINSRYVFL-M
PQUADEC.NiA.1.P   153 KVDVKINFIALDFMNDYNGY-MDDPDKPEEFEALNNRM-LTASYQCQEQSINSRYVFL-M
PBIA.V1_4.1.P00   153 KVDVKINFIALDFMNDYNGE-MDDPDRPEEFEALNNRM-LTASYQCQEQSINSRYVFL-M
PTRED.209.2.P71   153 KVDVKINFIALDFMNDYNGD-MDDPNKPEEFEALNNRM-LTASYQCQEQSINSRYIFL-M
PTET.51.1.P1140   153 KVDVKINFIALDFMNDYNGD-MDDPEKPEEFEALNNRM-LTASYQCQEQSINSRYVFL-M
POCTA.138.1.P04   153 KVDVKINFIALDFMNDYNGD-MDDPEKPEEFEALNNRM-LTASYQCQEQSINSRYVFL-M
PDEC.223.1.P001   153 KVDVKINFIALDFMNDYNGD-MDDPEKPEEFEALNNRM-LTASYQCQEQSINSRYVFL-M
PDODEC.274.1.P0   153 KVDVKINFIALDFMNDYNGD-MDDPEKPEEFEALNNRM-LTASYQCQEQSINSRYVFL-M
PBIA.V1_4.1.P02   153 KVDVKINFIALDFMNDYNGD-MDDPDRPEEFEALNNRM-LTASYQCQEQSINSRYVFL-M
PPENT.87.1.P105   153 KVDVKINFIALDFMNDYNGD-MDDPDKPEEFEALNNRM-LTASYQCQEQSINSRYVFL-M
PPRIM.AZ9-3.1.P   153 KVDVKINFIALDFMNDYNGD-MDDPDKPEEFEALNNRM-LTASYQCQEQSINSRYVFL-M
PDEC.223.1.P025   153 KVDVKINFIAMDFMNEYDVE-LDDPTKPENQEILNNRM-LNAVYQNQEQTINSRLVYQ-M
PDODEC.274.1.P0   153 KVDVKINFIAMDFMNEYDVE-LDDPTKPENQETLCNRM-LNAVYQNQEQTINSRLVYQ-M
PTET.51.1.P1510   153 KVDVKINFIAMDFMNEYDVE-LDDPTKPENQATLNDRM-LNAVYQHQEQSINSRLIYQ-M
POCTA.138.1.P07   153 KVDVKINFIALDFMNEYDAE-LDDPTKPENQVILNDRM-FNAVYQNQEQSINSRLIYQ-M
PSEX.AZ8_4.1.P1   153 KVDVKINFIALDFMNEYDVE-FDDPNKPENQEILNDRM-LNAVYETQEQQVNSRLIYY-M
PPENT.87.1.P146   153 KVDVKINFIALDFMNEYDVD-LDDPTKPENQEILNDRM-LNAVYENQQQSINSRLTYY-M
PPRIM.AZ9-3.1.P   153 KVDVKINFIALDFMNEYDVE-LDDPTKPENQEILNDRM-LNAVYENQQQSINSRLTYY-M
PBIA.V1_4.1.P00   153 KVDVKINFIALDFMNEYDVE-LDDPSKPQNQETLNDRM-LNAAYESQEQSINSRLVYY-M
PQUADEC.NiA.1.P   153 KVDVKINFIALDFMNEYDAE-LDDPSKPENQETLNDRM-LNAVYETKEQSINSRLTYY-M
POCTA.138.1.P15   153 KVDVKINFIALDFMNEYDAE-LDDPTKPENQETLNDRM-LNAVYESQEQSINSRLVYY-M
PTET.51.1.P1460   153 KVDVKINFIALDFMNEYDAE-LDDPSKPENQETLNDRM-LNAVYESQEQSINSRLVYY-M
PDODEC.274.1.P0   153 KVDVKINFIALDFMSEYDVE-LDDPTKPENQETLNDRM-LNAAYESQEQSINSRLVYY-M
PDEC.223.1.P028   153 KVDVKINFIALDFMNEYDVE-LDDPTKPENQETLNDRM-LNAAYESQEQSINSRLVYY-M
PNOV.TE.1.P0256   153 KVDVKINFIALDFMNEYDVE-LDDPSKPENQETLNDRM-LNAVYESQEQSINSRLTYY-M
PTRED.209.2.P71   153 KVDVKINFIALDFMNEYDVE-LDDPSKPESQETLNDRM-LNAVYETQEQSINSRLTYY-M
PSON.ATCC_30995   153 KVDVKINFIALDFMNEYDVE-LDDPTKPENQEILNNRM-LNSIYESKEQQINQNLIYY-M
PSON.ATCC_30995   154 KVDVKINFIALDFMNEYDVE-LDDPNKIDNQELLNDKM-LNAVYESQEQSINSNLIYY-M
PJENN.M.1.P0227   153 KVDVKINFIALDFMNEYNVE-LDDPNNPQNQEKLNDTM-LNAIYESKEQSINSHLTYY-M
PCAU.43c3d.1.P0   153 KVDVKINFIALDFMNDYDAE-MDDPNKPDEFEALNNRM-LTATYSCQEQSINSRYVFL-M
PMMNP16702        153 KVDVKINFIALDFMNDYDGD-MDDPNKPEELEALQNRI-LTASYQSQEQSINSRYIFL-M
```

```
Tpb1_BAI68043.1   201 ------------TSEEALQISLRIKPKQILQRPKYNGTFQIRNELTADVVVFTKTLEKHL
HsKu86_NP_06696   214 SLEGEDGLDEIYSFSESLRKLCVFK-KIERHSIHWPCRLTIGSNLSIRIAAYKSILQERV
Tpb6_DAA80465.1   192 QSQLKEHV-RIFTYDQAIQILKSFNPKPIQNRIKFKGTLQIAN-LNINVLIYTKTLERKF
TTHERM_00492460   201 KDELGEQI-QLFPSDVAIKIYEQFRTRQVNLRSKFNGDLQLAPNLNIAVQMFTKTSEEKL
PPOLY.Hb20-6.1.   209 TQELRENM-RIFPAHIAFELYSQFHTRSVSARASFRGDLQINDEYSIQVLIYKKTAEEKL
PTRED.209.2.P71   169 VQELRNNM-RIFPANVAFELYSQFHTKSQQARASFRGDFQINDEISIKVLIYKRCFEERL
PJENN.M.1.P0146   210 VQELRNNM-RIFPANVAFELYSQFHTRSLQARASFRGDFQINDEISIQVLIYKRCFEERL
PSON.ATCC_30995   210 VQELRNNM-RIFPANVAFELYSQFHTRSLQARASFRGDFQINDEISIQVLIYKRCFEERL
PSEX.AZ8_4.1.P0   210 VQELRNNM-RIFPANVAFELYSQFHTRSLQARASFRGDFQINDEISIQVLIYKRCFEERL
PQUADEC.NiA.1.P   210 VQELRNNM-RIFPANVAFELYSQFHTRSQQARASFRGDFQINDEISIQVLIYKRCFEERL
PBIA.V1_4.1.P00   210 VQELRNNM-RIFPANVAFELYSQFHTRCLQSRASFRGDFQINDEISIHVLIYKRCFEERL
PTRED.209.2.P71   210 VQELRNNM-RIFPANVAFELYSQFHTRSLQARASFRGDFQINDEISIQVLIYKRCFEERL
PTET.51.1.P1140   210 VQELRNNM-RIFPANVAFELYSQFHTRSLQARASFRGDFQINDEISVQVLIYKRCFEERL
POCTA.138.1.P04   210 VQELRNNM-RIFPANVAFELYSQFHTRSLQARASFRGDFQINDEISIQVLIYKRCFEERL
PDEC.223.1.P001   210 VQELRNNM-RIFPANVAFELYSQFHTRSLQARASFRGDFQINDEISIQVLIYKRCFEERL
PDODEC.274.1.P0   210 VQELRNNM-RIFPANVAFELYSQFHTRSLQARASFRGDFQINDEISIQVLIYKRCFEERL
PBIA.V1_4.1.P02   210 VQELRNNM-RIFPANAAFELYSQFHTKCLQARASFRGDFQINDEISIQVLIYKRCFEERL
PPENT.87.1.P105   210 VQELRNNM-RIFPANVAFELYSQFHTRSLQARASFRGDFQINDEISIQVLIYKRCFEERL
PPRIM.AZ9-3.1.P   210 VQELRNNM-RIFPANVAFELYSQFHTRSLQARASFRGDFQINDEISIQVLIYKRCFEERL
PDEC.223.1.P025   210 VQELRNHM-RIFPANVAFELYSQFHTKQMQARASFRGDFQINDETSIQVLIYKRCAEDRL
PDODEC.274.1.P0   210 VQELRNHM-RIFPANVAFELYSQFHTKQMQARASFRGDFQINDETSIQVLIYKRCAEDRL
PTET.51.1.P1510   210 VQELRNHM-RIFPANIAFELYSQFHTKQMQARASFRGDFQINDETSIQVLIYKRCAEDRL
POCTA.138.1.P07   210 VQELRNHM-RIFPANIAFELYSQFHTKQMQARASFRGDFQINDETTIQVLIYKRCAEDRL
PSEX.AZ8_4.1.P1   210 VQELRNHM-RIFPANVAFELYSQFHTKQTQARASFRGDFQINDETLIQVLIYKRCAEERL
PPENT.87.1.P146   210 VQELRNHM-RIFPANIAFELYSQFHTKQMQARASFRGDFQINDETNISVLIYKRCTEEKL
PPRIM.AZ9-3.1.P   210 VQELRNHM-RIFPANIAFELYSQFHTKQMQARASFRGDFQINDETNIQVLIYKRCTEEKL
PBIA.V1_4.1.P00   210 VQELRNHM-RIFPANIAFELYSQFHTKQTSARASFRGDFQINDETSIQVLIYKRCAEERL
PQUADEC.NiA.1.P   210 VQELRNHM-RIFPANIAFELYSQFHTKQMSARASFRGDFQINDDTSIQVLIYKRCAEERL
POCTA.138.1.P15   210 VQELRNHM-RIFPANIAFELYSQFHTKQMQARASFRGDFQINDETSISVLVYKRCTEEKL
PTET.51.1.P1460   210 VQELRSHM-RIFPANIAFELYSQFHTKQMQARASFRGDFQINDETSISVLVYKRCTEEKL
PDODEC.274.1.P0   210 VQELRNHM-RIFPANIAFELYSQFHTKQMQARASFRGDFQINDETSISVLVYKRCTEEKL
PDEC.223.1.P028   210 VQELRNHM-RIFPANIAFELYSQFHTKQMQARASFRGDFQINDETSISVLVYKRCTEEKL
PNOV.TE.1.P0256   210 VQELRNHM-RIFPANIAFELYSQFHTKQMQARASFRGDFQINDETSIQVLIYKRCTEEKL
PTRED.209.2.P71   210 VQELRNHM-RIFPANIAFELYSQFHTKQMQARASFRGDFQINDETSIQVLIYKRCTEERL
PSON.ATCC_30995   210 VQELRNHM-RIFPANIAFELYSQFHTKQMQARASFRGDFQINDQTFLQVLIYKRCAEEKL
PSON.ATCC_30995   211 VQELRNHM-RIFPANIAFELYSQFHTKQMQARASFRGDFQINDQTYLQVLIYKRCTEERL
PJENN.M.1.P0227   210 VQELRNHM-RIFPANIAFELYSQFHTKQMQARASFRGDFQINDSTFIQVLVYKRCTEEKL
PCAU.43c3d.1.P0   210 VQELRNNM-RIFPANVAFELYSQFHTKQTNARASFRGDFQINDETSIQVLIYKRCSEEKL
PMMNP16702        210 VQELRNNM-RIFPANIAFELYSQFHTKQLQARASFRGDFQINDETSIQVLIYKRCSEERL
```

```
Tpb1_BAI68043.1  249 L-NLKTYSN-ITEWNPSTQRQ-----------MVEQQIQYYQVD-DILLENPVQDIS----
HsKu86_NP_06696  273 K---KTWTV-VD--AKTLKKE-----------DIQKETVYCLNDDDE-TEVLKEDII----
Tpb6_DAA80465.1  250 PFQLKEYFIKLDNNNKSVYKECKMIYDYIYEEQNNQLVDQNNEMDNNNINDENNIIPQQK
TTHERM_00492460  260 P-GLKMYSL-AVDFNPHCESG-----------QIERDVIMALQEDPNINPIDKSNIT----
PPOLY.Hb20-6.1.  268 P-SLKKHST-VGPFNKDPNGN-----------GIRNDAVLYIHDDPTMTPQEKEHVI----
PTRED.209.2.P71  228 P-TLKKHST-LGEFVTDIHKN-----------HVRNDLIYYNPEDPNMTPIEKENII----
PJENN.M.1.P0146  269 P-TLKKHST-LGQFQIDTNKN-----------HVRNDLIYYNPEDPNMTPIEKENII----
PSON.ATCC_30995  269 P-TLKKHST-LGQFQIDINKN-----------HVRNDLIYYNPEDPNMTPIEKENII----
PSEX.AZ8_4.1.P0  269 P-TLKKHST-LGQFQTDINKN-----------HVRNDLIYYNPEDPNMTPIEKDNII----
PQUADEC.NiA.1.P  269 P-TLKKHST-LDEFVSDIHKN-----------HVRNDLIYYNPEDPNMTPIEKENII----
PBIA.V1_4.1.P00  269 P-TLKKHST-LGQFQTHIQNN-----------HVRNDLIYYNPEDPNMTPIEKENII----
PTRED.209.2.P71  269 P-NLKKHST-LGEFVTDINKN-----------HVRNDLIYYNPEDPNMTPIEKENII----
PTET.51.1.P1140  269 P-TLRKHST-LGEFQTDTNKN-----------HVRNDLIYYNPEDPNMTPIEKDNII----
POCTA.138.1.P04  269 P-TLRKHST-LGEFQTDINKN-----------HVRNDLIYYNPEDPNMTPIEKDNII----
PDEC.223.1.P001  269 P-TLRKHST-LGEFQTDINKN-----------HVRNDLIYYNPEDPNMTPIEKDNII----
PDODEC.274.1.P0  269 P-TLRKHST-LGEFQTDINKN-----------HVRNDLIYYNPEDPNMTPIEKDNII----
PBIA.V1_4.1.P02  269 P-TLKKHST-LGQFQTDIHNN-----------HVRNDLIYYNPEDPNMTPIEKENII----
PPENT.87.1.P105  269 P-NLKKHST-LGEFQTDIHKN-----------HVRNDLIYYNPEDPNMTPIEKDNII----
PPRIM.AZ9-3.1.P  269 P-NLKKHST-LGEFQTDIHKN-----------HVRNDLIYYNPEDPNMTPIEKDNII----
PDEC.223.1.P025  269 P-SLKKHSA-IGEFSNEPSKN-----------IVRNDAIHYNPEDPNMTPIERENII----
PDODEC.274.1.P0  269 P-SLKKHSA-IGEFSNEPTRN-----------IVRNDTIHYNPEDPNMTPIERENII----
PTET.51.1.P1510  269 P-SLKKHSA-IGEYSSEPTRN-----------IVRNDSIHYNPEDPNMNPIERENII----
POCTA.138.1.P07  269 P-SLKKHSA-IGEFNSEPSRN-----------IVRNDSIHYNPEDPNMTPIERENII----
PSEX.AZ8_4.1.P1  269 P-SLKKHSA-IGEFSGETSKN-----------IVRNDTIHYNPEDPNMAPIEKENII----
PPENT.87.1.P146  269 P-SLKKHSA-IGDFSTEPSRN-----------IVRNDTIHYNPEDPNMNPIERENII----
PPRIM.AZ9-3.1.P  269 P-SLKKHSA-IGDFSTEPSRN-----------IVRNDTIHYNPEDPNMNPIERENII----
PBIA.V1_4.1.P00  269 P-SLKKHSA-IGEFNSEPQRN-----------VVRNDSIHYNPEDPNITPIERENII----
PQUADEC.NiA.1.P  269 P-SLKKHSG-IGEFSNEPSRN-----------VVRNDTIHYNPEDPNMTPIEKENII----
POCTA.138.1.P15  269 P-SLKKHSA-TGEFSSEPTRN-----------VVRNDTIHYNPEDPNMTPIERENII----
PTET.51.1.P1460  269 P-SLKKHSA-TGEFSSEPTRN-----------VVRNDTIHYNPEDPNMTPIERENII----
PDODEC.274.1.P0  269 P-SLKKHSA-TGEFSSEPSRN-----------VVRNDTIHYNPEDPNMTPIERENII----
PDEC.223.1.P028  269 P-SLKKHSA-TGEFSSEPSRN-----------VVRNDTIHYNPEDPNMTPIERENII----
PNOV.TE.1.P0256  269 P-SLKKHSG-TGEFSSEPTRN-----------VVRNDTIHYNPEDPNMTPIERENII----
PTRED.209.2.P71  269 P-SLKKHSG-IGEFNQEPSRN-----------VVRNDTIHYNPEDPNMTPIERENII----
PSON.ATCC_30995  269 P-SLKKHSA-IGEFSSEQNRN-----------IVRNDTIHYNPEDPNMAPIERENII----
PSON.ATCC_30995  270 P-SLKKHSA-IGEFSSETNRN-----------IVRNDTIHYNPEDPNMAPIEKENII----
PJENN.M.1.P0227  269 P-SLKKHSA-IGEFYSETNRN-----------IVRNDTIHYNPEDPNMAPIEKENII----
PCAU.43c3d.1.P0  269 P-SMKKHSG-IGEFNSEPSRN-----------VVRNDTIFYNQDDPNMTPIERENII----
PMMNP16702       269 P-SLKKHSG-IGDFNTEPTRN-----------LIRNDSISYNPEDPNMTPIDKENII----
```

```
Tpb1_BAI68043.1   292 -KYYKYGNQLVKMSELFLNQINLFTL-KEIKLIGSVQKSSIPRQSFMSGCDI-LFAKQDS
HsKu86_NP_06696   312 -QGFRYGSDIVPFSKVDEEQMKYKSEGKCFSVLGFCKSSQVQRRFFMGNQVLKVFAARDD
Tpb6_DAA80465.1   310 VRIYQYGNQLVQISDLMYEQTNLETY-SCLVLLGTVKAYSIPRSSFMRGTDV-VFAQ-KD
TTHERM_00492460   304 -KAYHYGKQLIPVTQALEAQMNYTSN-RELKVLGFVDSKKVPRQSFMAGVDI-IIANKND
PPOLY.Hb20-6.1.   312 -KGYVYGRVTVPIDSLMEERIKYQCP-RSFQLLGFVERKTIPRYYFMGSVDM-VVAV-EG
PTRED.209.2.P71   272 -RGYQYGRNLVPVDQIMEDKMKYQCP-RQFQLLGFVDRSHIPRYYYTSTVDM-VIAV-EN
PJENN.M.1.P0146   313 -RGYQYGRNLVPVDQIMEDKMKYQCP-RQFQLLGFVDRTQIPRYYYTSTVDM-VISV-EN
PSON.ATCC_30995   313 -RGYQYGRNLVPVDQIMEDKMKYQCP-RQFQLLGFVDRTQIPRYYYTSTVDM-VISV-EN
PSEX.AZ8_4.1.P0   313 -RGYQYGRNLVPVDQIMEDKMKYQCP-RQFQLLGFVDRSHIPRYYYTSTVDM-VIAV-EN
PQUADEC.NiA.1.P   313 -RGYQYGRNLVPVDQIMEDKMKYQCP-RQFQLLGFVDRFNIPRYYYTSTVDM-VIAV-EN
PBIA.V1_4.1.P00   313 -RGYQYGRNLVPVDQIMEDKMKYQCP-RQFQLLGFVDRSHIPRYYYISTVDM-VIAV-EN
PTRED.209.2.P71   313 -RGYQYGRNLVPVDQIMDDKMKYQCP-RQFQLLGFVDRSHIPRYYYISTVDM-VIAV-EN
PTET.51.1.P1140   313 -RGYQYGRNLVPVDQIMEDKMKYQCP-RQFQLLGFVDRSHIPRYYYTSTVDM-VIAV-EN
POCTA.138.1.P04   313 -RGYQYGRNLVPVDQIMEDKMKYQCP-RQFQLLGFVDRSHIPRYYYTSTVDM-VIAV-EN
PDEC.223.1.P001   313 -RGYQYGRNLVPVDQIMEDKMKYQCP-RQFQLLGFVDRSHIPRYYYTSTVDM-VIGV-EN
PDODEC.274.1.P0   313 -RGYQYGRNLVPVDQIMEDKMKYQCP-RQFQLLGFVDRSHIPRYYYTSTVDM-VIAV-EN
PBIA.V1_4.1.P02   313 -RGYQYGRNLVPVDQIMEDKMKYQCP-RQFQLLGFVDRSHIPRYYYISTVDM-VIAV-EN
PPENT.87.1.P105   313 -RGYQYGRNLVPVDQIMEDKMKYQCP-RQFQLLGFVDRSHIPRYYYISTVDM-VIAV-EN
PPRIM.AZ9-3.1.P   313 -RGYQYGRNLVPVDQIMEDKMKYQCP-RQFQLLGFVDRSHIPRYYYISTVDM-VIAV-EN
PDEC.223.1.P025   313 -KGYQYGRNLIPVDSIMEEKMKYQCS-RSFQLLGFVDRTQIPRHYFMFNVDM-VIAI-DC
PDODEC.274.1.P0   313 -KGYQYGRNLIPVDSIMEEKMKYQCS-RSFQLLGFVDRSQIPRHYFMFNVDM-VIAI-DC
PTET.51.1.P1510   313 -KGYQYGRNLIPVDSLMEEKMKYQCN-RSFQLLGFVERSQIPRHYFIFNVDM-VIAI-DC
POCTA.138.1.P07   313 -KGYQYGRNLIPVDSLMDEKMKYQCS-RSFQLLGFVDRSQIPRHYFMFNVDM-VIAI-DC
PSEX.AZ8_4.1.P1   313 -KGYLYGRNLIPVDSIMEDKMKYQCQ-RSFQLLGFVDRSQIPRQYFISNVDM-VVAV-DC
PPENT.87.1.P146   313 -KGYLYGRNLIPVDSIMEDKMKYQCT-RSFQLLGFVDKSQIPRHYFMSTVDM-VVSI-DC
PPRIM.AZ9-3.1.P   313 -KGYLYGRNLIPVDSIMEDKMKYQCP-RSFQLLGFVDKSQIPRHYFMSSVDM-VVSI-DC
PBIA.V1_4.1.P00   313 -KGYLYGRNLIPVDGLMEDKMKYQCV-RSFQLLGFVDKSQIPRHYFMSTVDM-VVSV-DC
PQUADEC.NiA.1.P   313 -KGYQYGRSLIPVDSIMEDKMKYQCP-RSFQLLGFVDRSQIPRHYFISNVDM-VVAI-DC
POCTA.138.1.P15   313 -KGYLYGRSLIPVDSIMEDKMKYQCP-RSFQLLGFVDKSQIPRHYFMSSVDM-VVAI-DC
PTET.51.1.P1460   313 -KGYLYGRSLIPVDSIMEDKMKYQCV-RSFQLLGFVDKSQIPRHYFMSSVDM-VVAI-DC
PDODEC.274.1.P0   313 -KGYLYGRSLIPVDSIMEDKMKYQCA-RSFQLLGFVDKSQIPRHYFMSSVDM-VVAI-DC
PDEC.223.1.P028   313 -KGYLYGRSLIPVDSIMEDKMKYQCV-RSFQLLGFVDKSQIPRHYFMSSVDM-VVAI-DC
PNOV.TE.1.P0256   313 -KGYLYGRSLIPVDSIMEDKMKYQCP-RSFQLLGFVDKSQIPRHYFMSSVDM-VVSI-DC
PTRED.209.2.P71   313 -KGYVYGRSLIPVDSIMEDKMKYQCP-RSFQLLGFVDKSQIPRHYFMSSVDM-IVSI-DC
PSON.ATCC_30995   313 -KGYQYGRSLIPVDSIMEDKMKYQCS-RTFQLLGFVERSQIPRHYFISNVDM-VVAV-DS
PSON.ATCC_30995   314 -KGYLYGRSLIPVDSIMEEKMKYQCP-RIFQLLGFVERSQIPRHYFMSNVDM-VVAV-DS
PJENN.M.1.P0227   313 -KGYLYGRSLIPVDSIMEEKMKYQCS-RTFQLLGFVERSQIPRHYFLSSVDM-VVAV-DS
PCAU.43c3d.1.P0   313 -KGYQYGRNLIPVDSLMEDKMKYQCP-RQFQLLGFVDRSQISRQYFMTSVDM-VVAV-DS
PMMNP16702        313 -KGYQYGRNLIPVDQLMEDKMKYQCP-RQFQLLGFVDRQQIPRYYYMSNVDM-VISV-ES
```

```
Tpb1_BAI68043.1    349 KRSRYIIASLIKACFEEQRYLVARFVLRQNSIPKLVVLIPHLKKNCEYFYIIELPTVESI
HsKu86_NP_06696    371 EAAAVALSSLIHALDDLDMVAIVRYAYDKRANPQVGVAFPHIKHNYECLVYVQLPFMEDL
Tpb6_DAA80465.1    367 VLSKKQFSAFVQALKDQNRYFVCRYVPRKNSVPRLVALIPYFASKYECFYLNELPTSESV
TTHERM_00492460    361 SVAKKGIAALCHSMIQTNKYAIARYVWRNNGAPKLCVLTPQIGKDYECLYMCQIPTSESV
PPOLY.Hb20-6.1.    368 EKAQKMLSALITALIATKKVAVARFVGRDKGSPKLMVLLPHKSARYECFWMIQLPTAEDI
PTRED.209.2.P71    328 QKQQTALTALVIALIATRKVAIARFVGREKTAPKIIMLLPHKSKNSQCFWMISLPTTEDI
PJENN.M.1.P0146    369 QKQQKALAALVIALIATRKVALARFVGRERTAPKLIMLLPHKSKNSQCFWMISLPTTEDI
PSON.ATCC_30995    369 QKQQKALAALVIALIATRKVAIARFVGRERTAPKLIMLLPHKSKNSQCFWMISLPTTEDI
PSEX.AZ8_4.1.P0    369 QKQQKALAALVIGLIATRKVAIARFVGRERTAPKLIMLLPHKSKNSQCFWMISLPTTEDI
PQUADEC.NiA.1.P    369 QKQQKALAALVIALIATRKVAIARFVGREKTAPKLIMLLPHKSKNSQCFWMISLPTTEDI
PBIA.V1_4.1.P00    369 QKQQKALAALVIALIATRKVAIARFVGREKTAPKLIMLLPHKSKNSQCFWMISLPTTEDI
PTRED.209.2.P71    369 QKQQKALAALVIALIATRKVAIARFVGREKTAPKLIMLLPHKSKNSQCFWMISLPTTEDI
PTET.51.1.P1140    369 QKQQKALAALVIALIATRKVALARFVGREKTAPKLIMLLPHKSKNSQCFWMISLPTTEDI
POCTA.138.1.P04    369 QKQQKALAALVIALIATRKVAIARFVGREKTAPKLIMLLPHKSKNSQCFWMISLPTTEDI
PDEC.223.1.P001    369 QKQQKALAALVIALIATRKVALARFVGREKTAPKLIMLLPHKSKNSQCFWMISLPTTEDI
PDODEC.274.1.P0    369 QKQQKALAALVIALIATRKVALARFVGREKTAPKLIMLLPHKSKNSQCFWMISLPTTEDI
PBIA.V1_4.1.P02    369 QKQQKALAALVIALIATRKVAIARFVGREKTAPKLIMLLPHKSKNSQCFWMISLPTTEDI
PPENT.87.1.P105    369 QKQQKALAALVIALIATRKVAIARFVGREKTAPKLIMLLPHKSKNSQCFWMISLPTTEDI
PPRIM.AZ9-3.1.P    369 QKQQKALAALVIALIATRKVAIARFVGREKTAPKLIMLLPHKSKNSQCFWMISLPTTEDI
PDEC.223.1.P025    369 EKARKSLSALIIALIATKKVAIARFVGREKSSPKLMVLLPHKSKSYQCFWMISLPTSEDI
PDODEC.274.1.P0    369 EKARKSLSALIIALIATKKVAIARFVGREKSSPKLMVLLPHKSKSYQCFWMISLPTSEDI
PTET.51.1.P1510    369 EKARKSLSALIIALIATKKVAIARFVGRQKSSPKLMLLLPHKSKSYQCFWMISLPTSEDI
POCTA.138.1.P07    369 EKAKKSLSALIIALIATKKVAIARFVGREKSSPKLMVLLPHKSKSYQCFWMISLPTSEDI
PSEX.AZ8_4.1.P1    369 EKSKKSLSALIIALIATKKVAIARFVGREKSSPKMVVLLPHKSKSYQCFWMIALPTSEDI
PPENT.87.1.P146    369 EKAKKSLSSLIIALIATKKVAIARFVGREKGSPKLVVLLPHKSKSYSCFWMISLPTTEDI
PPRIM.AZ9-3.1.P    369 EKAKKSLSSLIIALIATKKVAIARFVGREKGSPKLVVLLPHKSKSYSCFWMISLPTTEDI
PBIA.V1_4.1.P00    369 EKSRKSLSSLIIALIATKKVAIARFVGREKGSPKMVVLLPHKSKSYQCFWMISLPTSEDI
PQUADEC.NiA.1.P    369 EKAKKSLSSLIIALIATKKVAIARFVGREKSSPKMMLLLPHKSKSYQCFWMISLPTSEDI
POCTA.138.1.P15    369 EKAKKSLSSLIIALIATKKVAIARFVGREKSSPKMVVLLPHKSKSYQCFWMIALPTSEDI
PTET.51.1.P1460    369 EKAKKSLSSLIIALIATKKIAIARFVGREKSSPKMVVLLPHKSKSYQCFWMIALPTSEDI
PDODEC.274.1.P0    369 EKAKKSLSSLIIALIATKKVVIARFVGREKSSPKMVVLLPHKSKSYQCFWMISLPTSEDI
PDEC.223.1.P028    369 EKAKKSLSSLIIALIATKKVAIARFVGREKSSPKMVVLLPHKSKSYQCFWMISLPTSEDI
PNOV.TE.1.P0256    369 EKAKKSLSSLIIALIATKKVAIARFVGREKSSPKLMLLLPHKSKSYQCFWMISLPTSEDV
PTRED.209.2.P71    369 EKAKKSLSSLIIALIATKKVAIARFVGREKSSPKLMVLLPHKSKSYQCFWMISLPTSEDI
PSON.ATCC_30995    369 EQSKKCLSALIIALIATKKVAIARFVGREKGSPKMVVLLPHKSKSYQCFWMITLPTSEDI
PSON.ATCC_30995    370 EQSQKTLSALIIALIATKKVAIARFVGREKASPKMVVLLPHKSKSYSCFWMIALPTSEDI
PJENN.M.1.P0227    369 EQSKKSLSALIIALIATKKVAIARFVGREKSSPKMVLLLPHKSKSYQCFWMINLPTSEDI
PCAU.43c3d.1.P0    369 EKAQKSLSALIVALIATRKVAIARFVGRERASPKLMVLLPHKSKSYQCFWMIALPTIEDI
PMMNP16702         369 EKSQKALSALIIALIATRKVAVARFVGREKSSPKLMVLLPHKSKSYQCFWMIQLPTTEDI
```

```
Tpb1_BAI68043.1    409 RDYSFNSLIRS-----TPEQQKLMSQLIDELDLD----QDEKNQNQFKIGSQPNETIAKI
HsKu86_NP_06696    431 RQYMFSSLKNSKKYAPTEAQLNAVDALIDSMSLAKKDEKTDTLEDLFPTTKIPNPRFQRL
Tpb6_DAA80465.1    427 RQYPFNSLKQS-----TEIQQVISKLIDNMDLEKENIQ-------FDLKQIKNPFFQSI
TTHERM_00492460    421 RDFQFNSLKES-----TKEQQDLMGSLIDKMDL--MNLEDG--EEALQMKYTFNPTRQYF
PPOLY.Hb20-6.1.    428 RHFQFASLRRS-----TPQQQHTVGQLIDSMDLEKIPNEEGVPEEILKMKCIANPTKQYF
PTRED.209.2.P71    388 RHFQFAALKRS-----TPPQQMAISALIDCMDLEKIPTEDGKFEELLKMKYIANPTRQYF
PJENN.M.1.P0146    429 RHFQFAALKRS-----TPNQQMAVAAMIDSMDLQKMPTEDGQFEELLKMKYVANPTRQYF
PSON.ATCC_30995    429 RHFQFAALKRS-----TPNQQMAVAAMIDSMDLQKMPTEDGQFEELLKMKYVANPTRQYF
PSEX.AZ8_4.1.P0    429 RHFQFAALKKS-----NPTQQMAVAAMIDSMDLQKMPTEDGQFEELLKMKYVANPTRQYF
PQUADEC.NiA.1.P    429 RHFQFAALKRS-----TPPQQMAVSAMIDSMDLEKMPTEDGQFEELLKMKYVANPTRQYF
PBIA.V1_4.1.P00    429 RHFQFAALKRS-----TPPQQMAVSAMIDNMDLEKMPTEDGQFEELLKLKYVANPTRQYF
PTRED.209.2.P71    429 RHFQFAALKRS-----TPPQQMAVSAMIDSMDLEKMPTEDGQFEELLKMKYVANPTRQYF
PTET.51.1.P1140    429 RHFQFAALKRS-----TPPQQMAVSAMIDCMDLEKMPTEDGQFEELLKMKYVANPTRQYF
POCTA.138.1.P04    429 RHFQFAALKRS-----TPPQQMAVSAMIDSMDLEKMPTEDGQFEELLKMKYVANPTRQYF
PDEC.223.1.P001    429 RHFQFAALKRS-----TPPQQMAVSAMIDSMDLEKMPTEDGQFEELLKMKYVANPTRQYF
PDODEC.274.1.P0    429 RHFQFAALKRS-----TPPQQMAVSALIDSMDLEKMPTEDGQFEELLKMKYVANPTRQYF
PBIA.V1_4.1.P02    429 RHFQFAALKRS-----TPPQQMAVSTMIDSMDLEKMPTEDGQFEELLKMKYVANPTRQYF
PPENT.87.1.P105    429 RHFQFAALKRS-----TPPQQMAVSAMIDSMDLEKMPTEDGQFEELLKMKYVANPTRQYF
PPRIM.AZ9-3.1.P    429 RHFQFAALKRS-----TPPQQMAVSAMIDSMDLEKMPTEDGQFEELLKMKYVANPTRQYF
PDEC.223.1.P025    429 RHFQFSALRKS-----NPNQQTAVATLIDKMDLETLPNESGEAEQLLKMKYIANPTRQYF
PDODEC.274.1.P0    429 RHFQFSAFRKS-----NPNQQAAVATLIDKMDLETLPNESGEAEQLLKMKYIANPTRQYF
PTET.51.1.P1510    429 RHFQFSTLRKS-----TPNQQSAVATLIDKMNLETIPNESGEPEELLKMKYIANPTRQYF
POCTA.138.1.P07    429 RHFQFSALRKS-----TPNQQAAVASLIDKMNLETLPNESGEPEELLKMKYIANPTRQYF
PSEX.AZ8_4.1.P1    429 RHFQFAALRKS-----NPNQQIAVASLIDKMDLENIPNESGEPEQLLKMKYVANPTRQYF
PPENT.87.1.P146    429 RHFQFAALRKS-----TPNQQIAVASLIDKMDLENLPNQSGEPEELLKMKYVANPTRQYF
PPRIM.AZ9-3.1.P    429 RHFQFAALRKS-----TPNQQIAVASLIDKMDLENLPNQSGEPEELLKMKYVANPTRQYF
PBIA.V1_4.1.P00    429 RLFQFAALRKS-----TPNQQIAVASLIDKMDLETLPNESGEPEELLKMKYVANPTRQYF
PQUADEC.NiA.1.P    429 RHFQFAALRKS-----TPNQQIAVASLIDKMDLENLPNQSGEPEELLKMKYVANPTRQYF
POCTA.138.1.P15    429 RHFQFAALRKS-----TPHQQIAVASLIDKMDLEALPNESGEPEELLKMKYVANPTRQYF
PTET.51.1.P1460    429 RHFQFAALRKS-----TPHQQIAVASLIDKMDLEALPNESGEPEELLKMKYIANPTRQYF
PDODEC.274.1.P0    429 RHFQFAALRKA-----TPNQQTAVASLIDKMDLETLPNQSGEPEELLKMKYIANPTRQYF
PDEC.223.1.P028    429 RHFQFAALRKS-----TPHQQIAVASLIDKMDLETLPNESGEPEELLKMKYIANPTRQYF
PNOV.TE.1.P0256    429 RHFQFAALRKS-----TPNQQIAVATLIDKMDLESLPNQSGEPEELLKMKYVANPTRQYF
PTRED.209.2.P71    429 RHFQFAALRKS-----TPNQQIAVASLIDKMDLESIPNQSGEPEELLKMKYVANPTRQYF
PSON.ATCC_30995    429 RHFQFSALRKS-----TPNQQIAVASLIDKMDLETIPNQSGEPEELLKMKYVANPTRQYF
PSON.ATCC_30995    430 RHFQFQTLRKS-----TPNQQIAVASLIDKMDLEKLPNQSGEPEELLKMKYIANPTRQYF
PJENN.M.1.P0227    429 RHFQFSTLRKS-----TPNQQIAVASLIDKMDLETLPNQSGEQEELLKMKYIANPTRQYF
PCAU.43c3d.1.P0    429 RHFQFSTLRKA-----TPAQQHAVACMIDNMDLEKMPNEDGQYEELLKMKYVANPTRQYF
PMMNP16702         429 RHFQFSALRKS-----TPNQQSAVSQMIDNMDLQKIPNEDGQQEELLKMKYIANPTKQYF
```

```
Tpb1_BAI68043.1   460 NDLILMRGMNV----------NEE-----EINKKLFTKEYQQTF--------KSASQKLL
HsKu86_NP_06696   491 FQCLLHRALHP----------REP---LPPIQQHIWNMLNPPAEV-------TTKSQIPL
Tpb6_DAA80465.1   475 NYFIFQKLIKNKFENKEEQDESET---LEQIKAETINYMKQKLIIEKQFLDCQNETKQIL
TTHERM_00492460   472 YQTVFHRVFNP----------PDADTKIPPLDPNIRDYITPEKKV-------YPKAAEEL
PPOLY.Hb20-6.1.   483 QQVVMNKAITQ----------TDI---VPEISPVILDYLHPEGRV-------YQLSKDIL
PTRED.209.2.P71   443 QQVVMHKAITR----------SDI---LPPISPLILEYLHPEQRV-------YNYAKEAL
PJENN.M.1.P0146   484 QQVIMHKAITR----------SDV---LPPISPLILEYLHPEIRV-------YNYAKDAL
PSON.ATCC_30995   484 QQVIMHKAITR----------TDI---LPPISPLILEYLHPEIKV-------YDYAKEAL
PSEX.AZ8_4.1.P0   484 QQVIMHKAITR----------SDI---LPPISPLILEYLHPETRV-------YNYAKEAL
PQUADEC.NiA.1.P   484 QQVVMHKAITR----------SDI---LPPISPLILEYLHPETRV-------YNYAKEAL
PBIA.V1_4.1.P00   484 QQVIMHKAITR----------SDV---LPPISPLILEYLHPEIRV-------YNYAKEAL
PTRED.209.2.P71   484 QQVVMYKAITR----------SDV---LPPISPLILEYLHPEQKV-------YNYAKEAL
PTET.51.1.P1140   484 QQVVMHKAITR----------SDV---LPPISPLILEYLHPEKRV-------YDYAKEAL
POCTA.138.1.P04   484 QQVVMHKAITR----------SDV---LPPISPLILEYLHPEVRV-------YNYAKEAL
PDEC.223.1.P001   484 QQVVMHKAITR----------SDV---LPPISPLILEYLHPEMRV-------YNYSKEAL
PDODEC.274.1.P0   484 QQVVMHKAITR----------SDV---LPPISPLILEYLHPEMRV-------YNYAKEAI
PBIA.V1_4.1.P02   484 QQVVMHKAITR----------TDV---LPPISPLILEYLHPEIRV-------YNYAKEAL
PPENT.87.1.P105   484 QQVVMHKAITR----------TDV---LPPISPLILEYLHPEIRV-------YNYAKEAL
PPRIM.AZ9-3.1.P   484 QQVVMHKAITR----------TDV---LPPISPLILEYLHPEIRV-------YNYAKEAL
PDEC.223.1.P025   484 QQVVMHKAITR----------TDV---IPPISPLILDYLHPEQRV-------YNYAQDAI
PDODEC.274.1.P0   484 QQVVMHKAITR----------TDV---IPPISPLILDYLHPEQRV-------YNYAQDAI
PTET.51.1.P1510   484 QQVVMHKAITR----------TDV---IPPISPLILDYLHPERRV-------YNYAQDAI
POCTA.138.1.P07   484 QQVVMHRAITR----------TDV---IPPISPLILDYLHPERRV-------YNYAQDAI
PSEX.AZ8_4.1.P1   484 QQVIMHKAITR----------TDV---IPPISPLILDYLHPEQRV-------YNYAQDAI
PPENT.87.1.P146   484 QQVVMHKAITR----------TDV---IPPISPLILEYLHPEQRV-------YNYAQDAI
PPRIM.AZ9-3.1.P   484 QQVVMHKAITR----------TDV---IPPISPLILEYLHPEQRV-------YNYAQDAI
PBIA.V1_4.1.P00   484 QQVVMHKAITR----------TDV---IPPISPLILEYLHPEQRV-------YNYAQDAI
PQUADEC.NiA.1.P   484 QQVVMHKAITR----------TDV---IPPISPLILEYLHPEQRV-------YNYAQDAI
POCTA.138.1.P15   484 QQVVMHKAITR----------TDV---IPPISPLILEYLHPEQRV-------YNYAQDAI
PTET.51.1.P1460   484 QQVVMHKAITR----------TDV---IPPISPLILEYLHPEQRV-------YNYAQDAI
PDODEC.274.1.P0   484 QQVVMHKAITR----------TDV---IPPISPLILEYLHPEQRV-------YNYAQDAI
PDEC.223.1.P028   484 QQVVMHKAITR----------TDV---IPPISPLILEYLHPEQRV-------YNYAQDAI
PNOV.TE.1.P0256   484 QQVVMHKAITR----------TDV---IPPISPLILEYLHPEQRV-------YNYAQDAI
PTRED.209.2.P71   484 QQVVMHKAITR----------TDV---IPPISPLILEYLHPEQRV-------YNYAQDAI
PSON.ATCC_30995   484 QQVVMHKAITR----------TDV---IPPISPLILEYLHPESRI-------YNYAQDAI
PSON.ATCC_30995   485 QQVVMHKAITR----------TDV---IPPISPLILEYLHPEQRV-------YNYAQDAI
PJENN.M.1.P0227   484 QQVVMHKAITR----------TDV---IPPISPLILEYLHPEQRV-------YNYAQDAI
PCAU.43c3d.1.P0   484 QQVVMHKAITR----------TDV---IPPISPLILEYLHPEQRV-------YNNAKEAL
PMMNP16702        484 QQVVMHKAITK----------QDV---VPPISPLILEYLHPETKV-------NNYAKDSL
```

```
Tpb1_BAI68043.1   497 SNINEQFNLKIINQQLYHDSFNQKIYWKNLIS---------------QK--D---DKL-F
HsKu86_NP_06696   531 SKIKTLFPL----IEAKKK---DQVTAQEIFQ---------------DN-----------
Tpb6_DAA80465.1   532 SQIKNIFNLEFDNKYKENQ---NKVFWQSLYN---------------KN--SS--SQLEK
TTHERM_00492460   515 KKIKESFQLQEQEIKDEIS---KKIFWQQLFNPNYQVPAANINAPQVQI--KMENGQMES
PPOLY.Hb20-6.1.   523 QRLKAVFKFKKHEIKKPQE---KKVFWKQLLE---------------QE--SQ--TQV--
PTRED.209.2.P71   483 QRIKSVFKFKINELKKQSD---KKVFWKQLFE---------------EQ--ST--EQI-Q
PJENN.M.1.P0146   524 SKIKTAFKFKINELKKQSD---KKVFWKQLFE---------------QQ--ST--TQ--Q
PSON.ATCC_30995   524 SKIKAAFKFKINELKKQTD---KKVFWKQLFD---------------QQ--ST--TQ--Q
PSEX.AZ8_4.1.P0   524 AKVKAAFKFKINEIKKQSD---KKVFWKQLFE---------------QQ--ST--EQI-Q
PQUADEC.NiA.1.P   524 QRIKSAFKFKVNELKKQAD---KKVFWKQLFE---------------EQ--ST--EQV-Q
PBIA.V1_4.1.P00   524 QRVKGAFKFKINEMKKQSD---KKVFWKQLFE---------------EQ--ST--EQV-Q
PTRED.209.2.P71   524 QRIKSAFKFKINELKKQSD---KKVFWKQLFE---------------EQ--ST--EQI-Q
PTET.51.1.P1140   524 QKVKAAFKFKINEIKKQGD---KKVFWKQLFE---------------DQ--ST--EQI-Q
POCTA.138.1.P04   524 QKVKAAFKFKINEIKKQGD---KKVFWKQLFE---------------EQ--ST--EQI-Q
PDEC.223.1.P001   524 QKIKAAFKFKINEIKKQGD---KKVFWKQLFE---------------EQ--ST--EQI-Q
PDODEC.274.1.P0   524 QKIKAAFKFKINEIKKQGD---KKVFWKQLFE---------------EQ--ST--EQI-Q
PBIA.V1_4.1.P02   524 QRIKGVFKFKINEMKKQSD---KKVFWKQLFE---------------EQ--ST--EQV-Q
PPENT.87.1.P105   524 QKIKTAFKFKVNEMKKQSD---KKVFWKQLFE---------------EQ--ST--EQV-Q
PPRIM.AZ9-3.1.P   524 QKIKTAFKFKVNEMKKQSD---KKVFWKQLFE---------------EQ--ST--EQV-Q
PDEC.223.1.P025   524 QRVKNAFKFKVNDIKKPQD---KKVFWKQLFD---------------EQ--TA-QQQV-P
PDODEC.274.1.P0   524 QRVKNAFKFKVNEIKKQQD---KKVFWKQLFD---------------EQ--TA-EQQV-P
PTET.51.1.P1510   524 QRVKNAFKFKVNDIKKPQD---KKVFWKQLFD---------------EQ--TA-QQQV-P
POCTA.138.1.P07   524 QRVKNAFKFKLNEIKKPQD---KKVFWKQLFD---------------EQ--TA-QQQI-P
PSEX.AZ8_4.1.P1   524 QRVKNAFKFKVNEIKKPQE---KKVFWKQLFD---------------EQ--TT--QQV-Q
PPENT.87.1.P146   524 QRVKNAFKFKVNEIKQPQD---KKVFWKQLFD---------------EQ--TT--QQV-Q
PPRIM.AZ9-3.1.P   524 QRVKNAFKFKVNEIKQQQD---KKVFWKQLFD---------------EQ--TT--QQV-Q
PBIA.V1_4.1.P00   524 QRVKNAFKFKVNEIKKPQD---KKVFWKQLFD---------------EQ--TTQQQQV-Q
PQUADEC.NiA.1.P   524 QKVKNAFKFKVNEIKKPQD---KKVFWKQLFD---------------EQ--TT-QQQI-Q
POCTA.138.1.P15   524 QRVKNAFKFKVNEIKKPQD---KKVFWKQLFD---------------EQ--TTQQQQV-Q
PTET.51.1.P1460   524 QRVKNAFKFKVNEIKKPQD---KKVFWKQLFD---------------EQ--TTQQQQA-Q
PDODEC.274.1.P0   524 QRVKNAFKFKVNEIKKPQD---KKVFWKQLFD---------------EQ--TTQQQQI-Q
PDEC.223.1.P028   524 QRVKNAFKFKVNEIKKPQD---KKVFWKQLFD---------------EQ--TTQQQQI-Q
PNOV.TE.1.P0256   524 QRVKNAFKFKVNEIKKSQD---KKVFWKQLFD---------------EQ--TT-QQQV-Q
PTRED.209.2.P71   524 QRVKNAFKFKVNEIKKPQD---KKVFWKQLFD---------------EQ--TT-QQQV-Q
PSON.ATCC_30995   524 KRVKNAFKFKVNEIKKPQD---KKVFWKQLFD---------------EQ--TT--QQV-Q
PSON.ATCC_30995   525 QRVKNAFKFKVNEIKKPQD---KKVFWKQLFD---------------EQ--TT--QQV-Q
PJENN.M.1.P0227   524 QRVKNAFKFKVNEIKKPQD---KKVFWKQLFD---------------EQ--TT--QQV-Q
PCAU.43c3d.1.P0   524 QKVKNAFKFKVNEIKKQQD---KKVFWKQLFD---------------EQ--ST--QQV--
PMMNP16702        524 QKVKNAFKFKVNEIKKQQD---KKVFWKQLFD---------------EQQSTT--QQIQQ
```

```
Tpb1_BAI68043.1   536 NDLKDIFGSIEKQNYKDLTKKAYQQIDFS-MPQSKIISILS--QPLFDIREF--------
HsKu86_NP_06696   558 --HEDGPTAKKLKTEQGGAHFSVSSLA------EGSVTSVGSVNPAENFRVLVKQKKASF
Tpb6_DAA80465.1   570 KNNLISNKQIDKQSQQNDEILIYQNIIIS---QNQQLFPIEIMNVIQEFNLIKQQEDIEN
TTHERM_00492460   570 EDVEQQAQILVDVKEELENIPISKKFAFDDYNKDDVVRKVSSVSPVDDFFKMMTNKREDL
PPOLY.Hb20-6.1.   559 IEQTDEVVEINQEEEEMVQMLAKQKYGFK----DEMVKEIGTIDPVEDFRKMVTEKRADL
PTRED.209.2.P71   520 QQIEDEVIEINQEEEEMVNMFAKQKFGFN----DEIVKEIGTVDPISDFKLMITEKRIDL
PJENN.M.1.P0146   560 IQPDDEVVEINQEEEEMVSMFAKQKLGFN----DEIIKEIGTVDPISDFKKMITEKRIDL
PSON.ATCC_30995   560 IQPDDEVVEINQEEEEMVSMFAKQKLGFN----DEIIKEIGTVDPISDFKKMITEKRIDL
PSEX.AZ8_4.1.P0   561 PQVEDEVVEINQEEEEMVNMFAKQKLGFN----DDIIKEIGTVDPISDFKKMITEKRIDL
PQUADEC.NiA.1.P   561 QQVEDEVVEINQEEEEMVNMFAKQKLGFN----DEIIKEIGTVDPISDFKKMITEKRIDL
PBIA.V1_4.1.P00   561 QQVEDEVVEINQEEEEMVNMFAKQKLGFN----DDIIKEIGTVDPISDFLKMITEKRIDL
PTRED.209.2.P71   561 QQVEDEVVEINQEEEEMVNMFAKQKLGFN----DEIVKEIGTVDPISDFRKMITEKRIDL
PTET.51.1.P1140   561 QQVEDEVVEINQEEEEMVNMFAKQKLGFN----DDIVKEIGTVDPTSDFRKMITEKRVDL
POCTA.138.1.P04   561 QQVEDEVVEINQEEEEMVNMFAKQKLGFN----DDIIKEIGTVDPTSDFRKMITEKRVDL
PDEC.223.1.P001   561 QQVEDEVVEINQEEEEMVNMFAKQKLGFN----DDIVQEIGTVDPTSDFRKMITEKRVDL
PDODEC.274.1.P0   561 QQVEDEVVEINQEEEEMVNMFAKQKLGFN----DDIVQEIGTVDPTSDFRKMITEKRVDL
PBIA.V1_4.1.P02   561 QQVEDEVVEINQEEEEMANMFAKQKLGFN----DDIIKEIGTVDPISDFKKMITEKRIDL
PPENT.87.1.P105   561 QQVEDEVVEINQEEEEMVNMFAKQKLGFN----DDIIKEIGTVDPISDFKKMITEKRIDL
PPRIM.AZ9-3.1.P   561 QQVEDEVVEINQEEEEMVNMFAKQKLGFN----DDIIKEIGTVDPISDFKKMITEKRIDL
PDEC.223.1.P025   562 EQIEEEVVEINREEEEMVNMFAKQKFGFN----DDIIQEIGSVDPISDFKKMITEKRVDQ
PDODEC.274.1.P0   562 QQIEEEVVEINREEEEMVNMFAKQKFGFN----DDIIQEIGSVDPISDFKKMITEKRVDQ
PTET.51.1.P1510   562 EQIEEEIVEINREEEEMVNMFAKQKFGFN----DDIIQEIGSVDPISDFKKMITEKRVDL
POCTA.138.1.P07   562 QQIEEEIVEINREEEEMVNMFAKQKFGFN----DDIIQEIGSVDPISDFKKMITEKRVDL
PSEX.AZ8_4.1.P1   561 QQIEEELVEINREEEEMVNMFAKQKLGFN----DDIIQEIGSVDPISDFKKMITEKRVDQ
PPENT.87.1.P146   561 QQIQEEVVEINREEEEMVNMFAKQKLGFN----DDIIQEIGSVDPISDFKKMITEKRVDQ
PPRIM.AZ9-3.1.P   561 QQIQEEVVEINREEEEMVNMFAKQKLGFN----DDIIQEIGSVDPISDFKKMITEKRVDQ
PBIA.V1_4.1.P00   563 QQIEEEVVEINREEEEMVNMFAKQKLGFN----DDIIQEIGSVDPISDFKKMITEKRVDQ
PQUADEC.NiA.1.P   562 QQIEEEVVEINREEEEMVNMFSKQKLGFN----DDIIQEIGSVDPISDFKKMITEKRVDQ
POCTA.138.1.P15   563 QQIEEEVVEINREEEEMVNMFAKQKLGFN----DDIIQEIGSVDPISDFKKMITEKRVDL
PTET.51.1.P1460   563 QQIEEEVVEINREEEEMVNMFAKQKLGFN----DDIIQEIGSVDPISDFKKMITEKRVDL
PDODEC.274.1.P0   563 QQIEEEVVEINREEEEMVNMFAKQKMGFN----DDIIQEIGSVDPISDFKKMITEKRVDL
PDEC.223.1.P028   563 QQIEEEVVEINREEEEMVNMFAKQKLGFN----DDIIQEIGSVDPISDFKKMITEKRVDL
PNOV.TE.1.P0256   562 QQIEEEVVEINREEEEMVNMFAKQKLGFN----DDIIQEIGSVDPISDFKKMITEKRVDQ
PTRED.209.2.P71   562 QQLEEEVVEINREEEEMVNMFAKQKLGFN----DDIIQEIGSVDPISDFKKMITEKRVDQ
PSON.ATCC_30995   561 QQIEEEVVEINREEEEMVNMFAKQKLGFK----DDIIQEIGSVDPISDFKKMITEKRVDQ
PSON.ATCC_30995   562 QQIEEEVVEINREEEEMVNMFAKQKLGFK----DDIIEEIGSVDPISDFKKMITEKRVDQ
PJENN.M.1.P0227   561 QQIEEEVVEINREEEEMVNMFAKQKLGFK----DDIIQEIGSVDPISDFKKMITEKRVDQ
PCAU.43c3d.1.P0   560 QQVEEEVVEINREEEEMVNMFAKQKLGFN----DDIIQEIGSVDPISDFKKMITEKRVDQ
PMMNP16702        564 QQVEEEVIEINREEEEMVTMFQKQKFGFN----DDIVQEIGSVDPISDFKKMITEKRVDQ
```

```
Tpb1_BAI68043.1   585 -QEYIQIVQNLIYKTMDTFIKGYTSNIIIELILLIRNKSIQFE---QYEAFNQFLLKFYE
HsKu86_NP_06696   610 -EEASNQLINHIEQFLDTNETP-YFMKSIDCIRAFREEAIKFS---EEQRFNNFLKALQE
Tpb6_DAA80465.1   627 -----QDYDSFVINLVQLKTENDF------------------------------------
TTHERM_00492460   630 VSDAISQIQGMISQLIETSIRGSYYEKALECIKAFRKGCTSEYEQLEAPKFNNFLNTFKD
PPOLY.Hb20-6.1.   615 VDSALTQMQRMIMRLVDESVRGNFFDKALECLREMRKACISED---EPDVFNKFLHHLKE
PTRED.209.2.P71   576 VDNALQQIQKVIIQFVDQSLKGSYYPKALECLKEMRIACITED---EAPVFNKYLHVLKE
PJENN.M.1.P0146   616 VDTALQQIQKIIISFVDQSLKGSFYPKALECLKEMRRACISED---ETNVFNKYLHALKD
PSON.ATCC_30995   616 VDTALQQIQKIIIQFVDQSLKGSFYPKALECLKEMRRACISED---ETNVFNKYLHALKD
PSEX.AZ8_4.1.P0   617 VDTALQQIQKVIIQFVDQSLKGSFYPKALECLKEMRRACISED---EAPIFNKYLHALKD
PQUADEC.NiA.1.P   617 VDNALQQIQKVIIQFVDQSLKGSFYPKALDCLKEMRKACITED---EAPVFNKYLHVLKE
PBIA.V1_4.1.P00   617 VDTALQQIQKVIIQLVDQSLKGSFYPKALECLKEMRKACITED---ETPVFNKYLNVLKD
PTRED.209.2.P71   617 VDTALQQIQKVIIQFVDQSLKGSYYPKALECLKEMRKACITED---EAPVFNKYLHVLKE
PTET.51.1.P1140   617 VDTALQQIQKVIIQFVDQSLKGSFYPKALECLKEMRKACITED---EAPVFNKYLHVLKE
POCTA.138.1.P04   617 VDTALQQIQKVIIQFVDQSLKGSFYPKALECLKEMRKACITED---EAPVFNKYLHVLKE
PDEC.223.1.P001   617 VDTALQQIQKVIIQFVDQSLKGSFYPKALECLKEMRKACITED---EAPVFNKYLHVLKE
PDODEC.274.1.P0   617 VDTALQQIQKVIIQFVDQSLKGSFYPKALECLKEMRKACITED---EAPVFNKYLHVLKE
PBIA.V1_4.1.P02   617 VDTALQQIQKVVIQLVDQSLKGSFYPKALECLKEMRRACITED---EAPIFNKYLHVLKE
PPENT.87.1.P105   617 VDTALQQIQKVVIQLVDQSLKGSFYPKALECLKEMRKACITED---EAPVFNKYLHVLKE
PPRIM.AZ9-3.1.P   617 VDTALQQIQKVVIQLVDQSLKGSFYPKALECLKEMRKACITED---EAPVFNKYLHVLKE
PDEC.223.1.P025   618 VDSALQQIQKVINALVDQSVKGSFFPKALECLKEMRKACISED---EAPVFNKFLFVLKD
PDODEC.274.1.P0   618 VDSALQQIQKVINALVDQSVKGSFFPKALECLKEMRKACISED---EAPVFNKFLFVLKD
PTET.51.1.P1510   618 VDSALQQIQKVINALVDQSVKGSFFPKALECLKEMRKACISED---EAPVFNKFLFVLKD
POCTA.138.1.P07   618 VDSALQQIQKVINSLVDQSVKGSFFPKALECLKEMRKACISED---EAPVFNKFLFVLKD
PSEX.AZ8_4.1.P1   617 VDSALQQMQKVIIGLVDQSVKGSFFPKALECLKEMRKACISED---EAPVFNKFLFVLKD
PPENT.87.1.P146   617 VDSALQQIQKVIIGLVDQSIKGSFFPKALECLKEMRKACISED---ETPVFNKFLFVLKD
PPRIM.AZ9-3.1.P   617 VDSALQQIQKVIISLVDQSIKGSFFPKALECLKEMRKACISED---ETPVFNKFLFVLKD
PBIA.V1_4.1.P00   619 VDSALQQIQKVIYGLVDQSVKGSFFPKALECLKEMRKACISED---ETPVFNKFLFVLKD
PQUADEC.NiA.1.P   618 VDSALQQIQKVIIGLVDQSVKGSFFPKALECLKEMRKACISED---EAPVFNKFLFVLKD
POCTA.138.1.P15   619 VDSALQQIQKVIIGLVDQSVKGSFFPKALECLKEMRKACISED---EAPVFNKFLFVLKD
PTET.51.1.P1460   619 VDSALQQIQKVIIGLVDQSVKGSFFPKALECLKEMRRACISED---EAPVFNKFLFVLKD
PDODEC.274.1.P0   619 VDSALQQIQKVIIGLVDQSVKGSFFPKALECLKEKRKACISED---EAPVFNKFLFVLKD
PDEC.223.1.P028   619 VDSALQQIQKVIIGLVDQSVKGSFFPKALECLKEMRKACISED---EAPVFNKFLFVLKD
PNOV.TE.1.P0256   618 VDSALQQIQKVISGLVDQSIKGSFFPKALECLKEMRKACISED---EAPVFNKFLFVLKD
PTRED.209.2.P71   618 VDSALQQIQKVIIGLVDQSVKGSFFPKAMECLKEMRKACISED---EAPVFNKFLFVLKD
PSON.ATCC_30995   617 VDSALQQMQKVIIGLVDQSVKGSFFPKALECLKEMRKACISED---EVPVFNKFLFVLKD
PSON.ATCC_30995   618 VDSALQQMQKVIIGLVDQSVKGSFFPKALECLKEMRKACISED---EAPIFNKFLFILKD
PJENN.M.1.P0227   617 VDSALQQMQKVIIGLVDQSIKGSFFPKALECLKEMRKACISED---EAPVFNKFLFVLKD
PCAU.43c3d.1.P0   616 VDSALQQMQKVIVGLVDSSVKGSFYPKALDCLKEMRKACISED---EAPIFNKYLYALKD
PMMNP16702        620 VDSALQQIQKVIIGLVDQSVKGSFYPKALECLKEMRKACISED---EAPIFNKYLNILKD
```

```
Tpb1_BAI68043.1   641 KF---QNDQFISEIVSSEINLISTENHQNSVILTQIEEEFL-RQNLLQKEMSSLRVSSKN
HsKu86_NP_06696   665 KVEIKQLNHFWEIVVQDGITLITKEEASGSSVTAEEAKKFLAPKDKPSGDTAAVFEEGGD
Tpb6_DAA80465.1   646 KY---RQLLQYAQNIQQTKEENINLDLSVKNLFTENCND-IQIKKISMSQPIRDFKYLTQ
TTHERM_00492460   690 KLLKSDQKGFWKMLIQQGITLITDKESTKSNFTTKEALQFLHQEEDVIQNIPEVRKQIED
PPOLY.Hb20-6.1.   672 KY---NQTLFWAKVVQDGTTLISRIDNGTSKVSVEEAQEFLNKEDDKNQIMVDQLQVADE
PTRED.209.2.P71   633 KY---SQLVFWAQIVQQGITLISNIENQKSNISVDDAQDFLNKEDIHHKQLVDQLQNEEE
PJENN.M.1.P0146   673 KY---SQLVFWAQIVQQGITLISNVENQKSHVTAEEAQEFLNKEDISHKQLVDQLQHEEE
PSON.ATCC_30995   673 KY---SQLVFWAQIVQQGITLISNVENQKSNVTAEEAQEFLNKEDISHKQLVDQLQHEEE
PSEX.AZ8_4.1.P0   674 KY---NQFVFWAQIVQQGITLISNVENQKSNVSAEEAQDFLNKEDINHKQLVDQLQHEEE
PQUADEC.NiA.1.P   674 KY---SQLVFWAQIVQQGITLISNIENQKSNVSADDAQEFLNKEDISHKQLVDQLQHEEE
PBIA.V1_4.1.P00   674 KY---SQLVFWAQIVQQGITLISNIENQKSNVNVDEAQDFLNKEDISHKQLVDQLQYEEE
PTRED.209.2.P71   674 KY---SQLVFWAQIVQQGITLISNIENQKSNISVDEAQEFLNKEDISHKQLVDQLQHEEE
PTET.51.1.P1140   674 KY---SQLVFWAQIVQQGITLISNIENQKSHVSVDEAQEFLNKEDISHKQLVDQLQHEEE
POCTA.138.1.P04   674 KY---SQLVFWAQIVQQGITLISNIENQKSHVSADEAQEFLNKEDISHKQLVDQLQHEEE
PDEC.223.1.P001   674 KY---SQLVFWAQIVQQGITLISNIENQKSHVTADEAQEFLNKEDISHKQLVDQLQHEEE
PDODEC.274.1.P0   674 KY---SQLVFWAQIVQQGITLISNIENQKSHVTADEAQEFLNKEDISHKQLVDQLQHEEE
PBIA.V1_4.1.P02   674 KY---SQLVFWAQIVQQGITLISNIENQKSNVSVDEAQEFLNKEDISHKQLVDQLQHEEE
PPENT.87.1.P105   674 KY---SQLVFWAQIVQQGITLISNIENQKSNISVDEAQEFLNKEDISHKQLVDQLQHEEE
PPRIM.AZ9-3.1.P   674 KY---SQLVFWAQIVQQGITLISNIENQKSNISVDEAQEFLNKEDISHKQLVDQLQHEEE
PDEC.223.1.P025   675 KY---NQQLFWAQIVQQGITLISNIENQKSSVTAEEAQDFLNKEDNKHQKMVDQLQNEEE
PDODEC.274.1.P0   675 KY---NQQLFWAQIVQQGITLISNIENQKSTVTAEEAQDFLNKEDNKHQQMVDQLQNEEE
PTET.51.1.P1510   675 KY---NQQLFWAQIVQQGITLISDIENHKSSVTAEEAQDFLNKEDNKHQQMVDQLQNEEE
POCTA.138.1.P07   675 KY---NQQLFWAQIVQQGITLISNIENQKSSVTAEEAQDFLNKEDNKHQQMVDQLQNEEE
PSEX.AZ8_4.1.P1   674 KY---NQQVFWAQVVQQGITLISNIENQKSTVTADEAQDFLNKEDNKHQQMVDQLQVEEE
PPENT.87.1.P146   674 KY---NQSMFWAQIVQQGITLISNIENQKSTVTAEEAQDFLNKEDNKHQQMVDQLQHEEE
PPRIM.AZ9-3.1.P   674 KY---NQSMFWAQIVQQGITLISNIENQKSTVTAEEAQDFLNKEDNKHQQMVDQLQHEEE
PBIA.V1_4.1.P00   676 KY---NQSMFWAQIVQQGITLISNIENQKSTVTAEEAQDFLNKEDNKHQQMVDQLQHEEE
PQUADEC.NiA.1.P   675 KY---NQSLFWAQIVQQGITLISKIENQKSTVSAEEAQDFLNKEDNKHQQMVDQLQHEDE
POCTA.138.1.P15   676 KY---NQSIFWAQIVQQGITLISNIENQKSTVTAEEAQDFLNKEDNKHQQMVDQLQHEEE
PTET.51.1.P1460   676 KY---NQSIFWAQIVQQGITLISNIENQKSGVTAEEAQDFLNKEDNKHQQMVDQLQHEEE
PDODEC.274.1.P0   676 KY---NQQMFWAQIVQQGITLISNIENQKSTVTAEEAQDFLNKEDNKHQQMVDQLQHEEE
PDEC.223.1.P028   676 KF---NQSMFWAQIVQQGITLISNIENQKSTVTAEEAQDFLNKEDNKHQQMVDQLQHEEE
PNOV.TE.1.P0256   675 KY---NQSIFWAQIVQQGITLISKIENLKSTVTAEEAQDFLNKEDNKHQQMVDQLQHEEE
PTRED.209.2.P71   675 KY---NQSMFWAQIVQQGITLISKIENQKSTVSAEEAQDFLNKEDNKHQKMVDQLQHEEE
PSON.ATCC_30995   674 KY---NQQVFWAQIVQQGITLISNIENQKSSVTADEAQDFLNKEDNKHQQMVDQLQNEEE
PSON.ATCC_30995   675 KY---NQQIFWAQIVQQGITLISNIENQKSSVTADEAQDFLNKEDNKHQQMVDTLQHEEE
PJENN.M.1.P0227   674 KY---NQQIFWASIVQQGITLISNIENQKSSVTAEEAQEFLNKEDNKHQQMVDTLQHEEE
PCAU.43c3d.1.P0   673 KY---NQLVFWAQIVQQGITLISNIENTKSTVSADDAQEFLNSEDKNHKSLVDQLQNEEE
PMMNP16702        677 KY---NQSVFWAQIVQQGITLISNIENSKSTVTAEDAQDF--------------QIRRQQS
```

```
Tpb1_BAI68043.1   697 QENICQNQ
HsKu86_NP_06696   725 VDDLLDMI
Tpb6_DAA80465.1   702 NVTIQKLQ
TTHERM_00492460   750 YDIMDELE
PPOLY.Hb20-6.1.   729 -DLLAEID
PTRED.209.2.P71   690 -DLLAEID
PJENN.M.1.P0146   730 -DLLAEID
PSON.ATCC_30995   730 -DLLAEID
PSEX.AZ8_4.1.P0   731 -DLLAEID
PQUADEC.NiA.1.P   731 -DLLAEID
PBIA.V1_4.1.P00   731 -DLLAEID
PTRED.209.2.P71   731 -DLLAEID
PTET.51.1.P1140   731 -DLLAEID
POCTA.138.1.P04   731 -DLLAEID
PDEC.223.1.P001   731 -DLLAEID
PDODEC.274.1.P0   731 -DLLAEID
PBIA.V1_4.1.P02   731 -DLLAEID
PPENT.87.1.P105   731 -DLLAEID
PPRIM.AZ9-3.1.P   731 -DLLAEID
PDEC.223.1.P025   732 -DLLADIE
PDODEC.274.1.P0   732 -DLLADIE
PTET.51.1.P1510   732 -DLLADIE
POCTA.138.1.P07   732 -DLLADIE
PSEX.AZ8_4.1.P1   731 -DLLADIE
PPENT.87.1.P146   731 -DLLADIE
PPRIM.AZ9-3.1.P   731 -DLLADIE
PBIA.V1_4.1.P00   733 -DLLADIE
PQUADEC.NiA.1.P   732 -DLLADIE
POCTA.138.1.P15   733 -DLLADIE
PTET.51.1.P1460   733 -DLLADIE
PDODEC.274.1.P0   733 -DLLADIE
PDEC.223.1.P028   733 -DLLADIE
PNOV.TE.1.P0256   732 -DLLADIE
PTRED.209.2.P71   732 -DLLADIE
PSON.ATCC_30995   731 -DLLADIE
PSON.ATCC_30995   732 -DLLADIE
PJENN.M.1.P0227   731 -DLLADIE
PCAU.43c3d.1.P0   730 -DLLADIE
PMMNP16702        721 -QIIS---
```
