# Supplementary material for: Functional diversification of Paramecium Ku80 paralogs safeguards genome integrity during precise programmed DNA elimination
Source: PLoS Genet. 2020 Apr 16;16(4):e1008723. doi: 10.1371/journal.pgen.1008723 (PMC7161955; doi:10.1371/journal.pgen.1008723)
Supplement: S1 File — KU80c regulatory sequences are in blue italics, coding sequences are in capital letters, with the 3X Flag- or GFP-coding sequence in green, KU80 sequences in capital italics (original ATG initiation codons are underlined) and the modified RNAi-resistant insert in red. Transgene initiation and stop codons are in bold. (DOCX) [file pgen.1008723.s017.docx]

**Supporting File 1. Sequences of *FLAG-KU80a, FLAG-KU80c*, *FLAG-KU80ca, FLAG-KU80ac* and *GFP-KU80a* plasmids used in micro-injection experiments**

*KU80c* regulatory sequences are in blue italics, coding sequences are in capital letters, with the 3X Flag- or GFP-coding sequence in green, *KU80* sequences in capital italics (original ATG initiating codons are underlined) and the modified RNAi-resistant insert in red. Transgene initiation and stop codons are in bold.

>pUC18-FLAG-Ku80c (RNAiResistant)

tcgcgcgtttcggtgatgacggtgaaaacctctgacacatgcagctcccggagacggtcacagcttgtctgtaagcggatgccgggagcagacaagcccgtcagggcgcgtcagcgggtgttggcgggtgtcggggctggcttaactatgcggcatcagagcagattgtactgagagtgcaccatatgcggtgtgaaataccgcacagatgcgtaaggagaaaataccgcatcaggcgccattcgccattcaggctgcgcaactgttgggaagggcgatcggtgcgggcctcttcgctattacgccagctggcgaaagggggatgtgctgcaaggcgattaagttgggtaacgccagggttttcccagtcacgacgttgtaaaacgacggccagtgccaagcttgcatgc***ttaaatagataaataatacaggcaattttttaatatacaaatttcaataatgaatttgtagatatatataaaaaataagtgcaaag*ATG**aattctaga**gactacaaagaccatgacggtgattataaagatcatgacatcgactacaaggatgacgatgataag**ggatcc*atgtctggaaaagaagctactctaatattattggatgttggagcctcaatgtatggtcaatattaatagggaggaagtaaaaagttgagcagattggaattggcagtagattgtttaggattgatgattcaataaaagatttttaattataagaatcacgaagtcggattgattttatttggaacagaagaagctcctgatggcaagacattgtacatccaagatttgtcaatcccagatttagatttctttaggaatattagtgatttacctaaccatgatgttggataataagtgggtggagatatatttgatgcattggacaaggcagtacatgctttagatgatcatgcaaagacaaaaaaaatggaaaagaagatctttatattaacagctggttgtggacagactgattacagtgaaaagtagataaccaaattaatcaagatgattgagaaagtggatgttaagataaatttcatagctcttgattttatgaatgattacaatggagatatggatgatcctgaaaaacctgaagaattcgaagctcttaacaataggatgctaacagcctcatatcagtgttaggaacaatccattaattcacgttacgttttcttgatggttcaagagttgaggaataacatgaggatatttccagctaatgttgcttttgagttatattcccagtttcatactagatcactataggctagagc****tt****catttaggggggacttccagattaacgatgaaatctctgtgcaagtgctaatttataagcgatgtttcgaggaaagattgccaactctaagaaagcattcaacccttggcgaattccaaacagatactaataaaaaccatgtgagaaatgatcttatctactataatccagaggatcctaatatgacaccaatcgaaaaagacaacatcataaggggatattaatatggcaggaatctcgtaccagttgattaaataatggaggataaaatgaagtactagtgtcctcgttaattttaattattaggctttgtagatcgatcccatatacctagatattattatacctcaactgttgacatggtcattgctgtagaaaatcagaagtaatagaaagctctggctgctctagtcattgcactcattgcaactaggaaagtagctcttgccagatttgtaggcagagagaaaacagcacctaaattgattatgttattaccccataaatccaagaactcttagtgcttctggatgatatccttgcctacaactgaagacatcaggcattttcaatttgctgctttgaagagatctactcctccataatagatggcagtctctgctatgattgactgtatggatctagaaaagatgcccacggaagatggacagtttgaagaacttcttaaaatgaagtatgttgccaatcctacgagataatacttccaataagtggtcatgcacaaagccatcactagatctgatgtattgcctcctatctcccctctcatcttggagtatttacatcctgaaaagagggtttacgattatgcaaaagaggcacttcaaaaagtcaaggctgctttcaaattcaaaatcaacgaaatcaaaaaacaaggggataagaaagtgttttggaaataactatttgaagatcaatctacagaataaatttaatagtaagtggaggatgaagtagttgaaatcaattaagaagaagaagagatggttaatatgtttgccaaataaaaattaggattcaatgacgatatagttaaggaaatcggcactgttgatcccacttcagatttcaggaagatgattactgaaaagagagtcgacttagttgacactgccttacaataaattcaaaaagtcattatttaatttgtggatcaatctctcaaaggcagtttctatccaaaagcacttgagtgtctcaaagaaatgaggaaagcctgtatcacagaagatgaagccccagtattcaataaatacttgcatgttcttaaagagaagtacagtcaacttgtcttctgggctcagattgtccaataaggaatcactttaatcagcaacattgagaattagaaatctcatgtttctgttgatgaagcacaagaagtaattcattattataatattagttcctaaataaagaggatattagtcataaataattagttgatcaattataacacgaagaagaggatctattggctgaaattgat****TGAtttattattagtctatatctaacaattttattccaatttttatattaataa***gagctcgcatgctctaattaaaccaagaacacgctgaattccatgctgcctaggttgttctttttcttgaatatttgcattcaaaccgtatattatatcggagtttgaattcatagcatttaatgattggatctgatgattacctaagataaattgtttttattttacagtattacacgaaagaaaaagaacagcttcatttcaccgattatgtcaaatcccaactcaagcttgagctcgaattcgtaatcatggtcatagctgtttcctgtgtgaaattgttatccgctcacaattccacacaacatacgagccggaagcataaagtgtaaagcctggggtgcctaatgagtgagctaactcacattaattgcgttgcgctcactgcccgctttccagtcgggaaacctgtcgtgccagctgcattaatgaatcggccaacgcgcggggagaggcggtttgcgtattgggcgctcttccgcttcctcgctcactgactcgctgcgctcggtcgttcggctgcggcgagcggtatcagctcactcaaaggcggtaatacggttatccacagaatcaggggataacgcaggaaagaacatgtgagcaaaaggccagcaaaaggccaggaaccgtaaaaaggccgcgttgctggcgtttttccataggctccgcccccctgacgagcatcacaaaaatcgacgctcaagtcagaggtggcgaaacccgacaggactataaagataccaggcgtttccccctggaagctccctcgtgcgctctcctgttccgaccctgccgcttaccggatacctgtccgcctttctcccttcgggaagcgtggcgctttctcatagctcacgctgtaggtatctcagttcggtgtaggtcgttcgctccaagctgggctgtgtgcacgaaccccccgttcagcccgaccgctgcgccttatccggtaactatcgtcttgagtccaacccggtaagacacgacttatcgccactggcagcagccactggtaacaggattagcagagcgaggtatgtaggcggtgctacagagttcttgaagtggtggcctaactacggctacactagaaggacagtatttggtatctgcgctctgctgaagccagttaccttcggaaaaagagttggtagctcttgatccggcaaacaaaccaccgctggtagcggtggtttttttgtttgcaagcagcagattacgcgcagaaaaaaaggatctcaagaagatcctttgatcttttctacggggtctgacgctcagtggaacgaaaactcacgttaagggattttggtcatgagattatcaaaaaggatcttcacctagatccttttaaattaaaaatgaagttttaaatcaatctaaagtatatatgagtaaacttggtctgacagttaccaatgcttaatcagtgaggcacctatctcagcgatctgtctatttcgttcatccatagttgcctgactccccgtcgtgtagataactacgatacgggagggcttaccatctggccccagtgctgcaatgataccgcgagacccacgctcaccggctccagatttatcagcaataaaccagccagccggaagggccgagcgcagaagtggtcctgcaactttatccgcctccatccagtctattaattgttgccgggaagctagagtaagtagttcgccagttaatagtttgcgcaacgttgttgccattgctacaggcatcgtggtgtcacgctcgtcgtttggtatggcttcattcagctccggttcccaacgatcaaggcgagttacatgatcccccatgttgtgcaaaaaagcggttagctccttcggtcctccgatcgttgtcagaagtaagttggccgcagtgttatcactcatggttatggcagcactgcataattctcttactgtcatgccatccgtaagatgcttttctgtgactggtgagtactcaaccaagtcattctgagaatagtgtatgcggcgaccgagttgctcttgcccggcgtcaatacgggataataccgcgccacatagcagaactttaaaagtgctcatcattggaaaacgttcttcggggcgaaaactctcaaggatcttaccgctgttgagatccagttcgatgtaacccactcgtgcacccaactgatcttcagcatcttttactttcaccagcgtttctgggtgagcaaaaacaggaaggcaaaatgccgcaaaaaagggaataagggcgacacggaaatgttgaatactcatactcttcctttttcaatattattgaagcatttatcagggttattgtctcatgagcggatacatatttgaatgtatttagaaaaataaacaaataggggttccgcgcacatttccccgaaaagtgccacctgacgtctaagaaaccattattatcatgacattaacctataaaaataggcgtatcacgaggccctttcgtc

>pUC18-promo*KU80c*-FLAG-*KU80a*-3’UTR*KU80c*

tcgcgcgtttcggtgatgacggtgaaaacctctgacacatgcagctcccggagacggtcacagcttgtctgtaagcggatgccgggagcagacaagcccgtcagggcgcgtcagcgggtgttggcgggtgtcggggctggcttaactatgcggcatcagagcagattgtactgagagtgcaccatatgcggtgtgaaataccgcacagatgcgtaaggagaaaataccgcatcaggcgccattcgccattcaggctgcgcaactgttgggaagggcgatcggtgcgggcctcttcgctattacgccagctggcgaaagggggatgtgctgcaaggcgattaagttgggtaacgccagggttttcccagtcacgacgttgtaaaacgacggccagtgccaagcttgcatgc***ttaaatagataaataatacaggcaattttttaatatacaaatttcaataatgaatttgtagatatatataaaaaataagtgcaaag*ATG**aattctagagactacaaagaccatgacggtgattataaagatcatgacatcgactacaaggatgacgatgataagggatcc*atggcaggaaaagaagcaactttagttttattagatgtaggtgcctcaatgtatgaaccatacaagtaggctcaaggaaagaaaatcactagattagaattagctgttgattgcatagggatgatgatttaataaaagatctttaattacaagaatcatgaagttggattagttttgtttggaacagaggacgctgaggatgggaatactttttacatctaaacattgtcgtctccagatctagagttttatagaaatctgacagaattacctaatcatgatatacctaaaataattggtggagatatttttgatgctttggacaaatcagttagcactttagatgagtatgtgaagaccaagaagatggataagaagatatttgtgctcacagcaggatttggttagacagaatataatgagaagaagattgcaaagcttattaaaatgattgaaaaagtagatgtgaagatcaatttcatagcattggatttcatgaatgaatatgatgctgaattggatgatccttccaaacctgagaatcaagaaactctaaatgatcgaatgcttaatgctgtttacgaaagttaagagcaatcaatcaattcacgtttagtttattacatggttcaagaactaaggagtcatatgagaatattcccagctaatatcgcatttgaattgtattcacaatttcacaccaaacaaatgcaagcaagagcctcatttagaggagattttcaaataaatgatgagacatccatctctgtattggtttacaaaagatgcacagaggagaaattaccaagtttaaaaaagcattctgctactggagaattcagcagtgaacccactagaaatgtagttagaaatgacaccattcattacaatccagaagacccaaacatgactccaattgaaagagagaatatcattaagggttacttatatggcagaagcctaattcctgtggatagtataatggaagataaaatgaagtattaatgtgttaggtcattttaattattgggatttgttgacaagtcataaattcctagacattattttatgtctagtgtggacatggttgtagctattgattgtgaaaaagcaaagaaatctttatcttctttgatcatagccttaattgccacaaagaaaattgctattgcaagatttgttggcagagaaaagagttctccaaaaatggtggtgttattgcctcataaatcaaagagctattagtgtttttggatgattgccttacccacttctgaagacatcaggcatttccaatttgctgctttaagaaagtccactcctcatcaataaatagctgtagcctccttgattgataaaatggatctagaagcacttccaaacgaatctggagaaccagaagaattacttaaaatgaaatacattgcaaatccaactagacaatatttctaataagtagtcatgcataaagctatcacaagaactgatgtcattcctccaatctctcctttgattctagaatatttgcatcccgaataacgagtatacaattatgcttaggatgctatatagagagtgaagaatgcttttaaattcaaagttaatgaaatcaagaaaccctaagataaaaaagtattttggaaacaattattcgatgaacaaactacataataacaacaagcttaacaataaatagaggaagaagtggttgaaatcaatagagaggaggaagagatggtcaatatgtttgccaaataaaaattgggattcaatgatgatatcatttaagaaattggttctgtggatccaatctcagatttcaagaaaatgatcactgaaaagagagtcgatttggttgattctgcccttcaacaaattcagaaggttataattggattagtcgattaatcagtaaaaggcagtttcttccccaaggccttggaatgcttaaaagaaatgagaagagcttgtatttcagaagatgaagctccagttttcaacaagttcctctttgtacttaaggataagtacaatcaatctatattttgggcccaaattgtacaataaggcatcactttgataagcaacatagagaattaaaaatcaggtgtcactgctgaggaagcataagatgtatatatatttaattaattattagttcctgaacaaagaagataacaaacattagtaaatggttgatcaattataacatgaggaagaagatttattagctgatattgaa****TGAtttattattagtctatatctaacaattttattccaatttttatattaataa***gagctcgcatgctctaattaaaccaagaacacgctgaattccatgctgcctaggttgttctttttcttgaatatttgcattcaaaccgtatattatatcggagtttgaattcatagcatttaatgattggatctgatgattacctaagataaattgtttttattttacagtattacacgaaagaaaaagaacagcttcatttcaccgattatgtcaaatcccaactcaagcttgagctcgaattcgtaatcatggtcatagctgtttcctgtgtgaaattgttatccgctcacaattccacacaacatacgagccggaagcataaagtgtaaagcctggggtgcctaatgagtgagctaactcacattaattgcgttgcgctcactgcccgctttccagtcgggaaacctgtcgtgccagctgcattaatgaatcggccaacgcgcggggagaggcggtttgcgtattgggcgctcttccgcttcctcgctcactgactcgctgcgctcggtcgttcggctgcggcgagcggtatcagctcactcaaaggcggtaatacggttatccacagaatcaggggataacgcaggaaagaacatgtgagcaaaaggccagcaaaaggccaggaaccgtaaaaaggccgcgttgctggcgtttttccataggctccgcccccctgacgagcatcacaaaaatcgacgctcaagtcagaggtggcgaaacccgacaggactataaagataccaggcgtttccccctggaagctccctcgtgcgctctcctgttccgaccctgccgcttaccggatacctgtccgcctttctcccttcgggaagcgtggcgctttctcatagctcacgctgtaggtatctcagttcggtgtaggtcgttcgctccaagctgggctgtgtgcacgaaccccccgttcagcccgaccgctgcgccttatccggtaactatcgtcttgagtccaacccggtaagacacgacttatcgccactggcagcagccactggtaacaggattagcagagcgaggtatgtaggcggtgctacagagttcttgaagtggtggcctaactacggctacactagaaggacagtatttggtatctgcgctctgctgaagccagttaccttcggaaaaagagttggtagctcttgatccggcaaacaaaccaccgctggtagcggtggtttttttgtttgcaagcagcagattacgcgcagaaaaaaaggatctcaagaagatcctttgatcttttctacggggtctgacgctcagtggaacgaaaactcacgttaagggattttggtcatgagattatcaaaaaggatcttcacctagatccttttaaattaaaaatgaagttttaaatcaatctaaagtatatatgagtaaacttggtctgacagttaccaatgcttaatcagtgaggcacctatctcagcgatctgtctatttcgttcatccatagttgcctgactccccgtcgtgtagataactacgatacgggagggcttaccatctggccccagtgctgcaatgataccgcgagacccacgctcaccggctccagatttatcagcaataaaccagccagccggaagggccgagcgcagaagtggtcctgcaactttatccgcctccatccagtctattaattgttgccgggaagctagagtaagtagttcgccagttaatagtttgcgcaacgttgttgccattgctacaggcatcgtggtgtcacgctcgtcgtttggtatggcttcattcagctccggttcccaacgatcaaggcgagttacatgatcccccatgttgtgcaaaaaagcggttagctccttcggtcctccgatcgttgtcagaagtaagttggccgcagtgttatcactcatggttatggcagcactgcataattctcttactgtcatgccatccgtaagatgcttttctgtgactggtgagtactcaaccaagtcattctgagaatagtgtatgcggcgaccgagttgctcttgcccggcgtcaatacgggataataccgcgccacatagcagaactttaaaagtgctcatcattggaaaacgttcttcggggcgaaaactctcaaggatcttaccgctgttgagatccagttcgatgtaacccactcgtgcacccaactgatcttcagcatcttttactttcaccagcgtttctgggtgagcaaaaacaggaaggcaaaatgccgcaaaaaagggaataagggcgacacggaaatgttgaatactcatactcttcctttttcaatattattgaagcatttatcagggttattgtctcatgagcggatacatatttgaatgtatttagaaaaataaacaaataggggttccgcgcacatttccccgaaaagtgccacctgacgtctaagaaaccattattatcatgacattaacctataaaaataggcgtatcacgaggccctttcgtc

> pUC18-promo*KU80c*-GFP-*KU80a*-3’UTR*KU80c*

tcgcgcgtttcggtgatgacggtgaaaacctctgacacatgcagctcccggagacggtcacagcttgtctgtaagcggatgccgggagcagacaagcccgtcagggcgcgtcagcgggtgttggcgggtgtcggggctggcttaactatgcggcatcagagcagattgtactgagagtgcaccatatgcggtgtgaaataccgcacagatgcgtaaggagaaaataccgcatcaggcgccattcgccattcaggctgcgcaactgttgggaagggcgatcggtgcgggcctcttcgctattacgccagctggcgaaagggggatgtgctgcaaggcgattaagttgggtaacgccagggttttcccagtcacgacgttgtaaaacgacggccagtgccaagcttgcatgc***ttaaatagataaataatacaggcaattttttaatatacaaatttcaataatgaatttgtagatatatataaaaaataagtgcaaag*ATG**aattctagaggagaagaacttttcactggtgttgttccaattcttgttgaacttgatggtgatgttaatggacataaattttctgtctctggtgagggtgaaggtgatgcaacttatggaaaattaaccttaaaatttatttgcactactggaaaattacctgttccatggccaacacttgtcactactttaacttatggagtccaatgtttttcaaggtaccctgaccacatgaaacaacatgactttttcaaatctgccatgccagaaggatatgtccaagaaagaactatattcttcaaagatgatggaaactacaagacaagagctgaagtcaaatttgaaggagatacccttgtcaatagaattgagcttaaaggaattgattttaaagaagatggaaacattttaggccataaattggaatacaactataactcacataatgtatacatcatggcagacaaacaaaaaaatggaatcaaagtcaacttcaaaattagacacaacattgaagatggatcagttcaattagcagaccattatcaacaaaatactcctattggagatggaccagtacttttaccagacaaccattacttatcaacacaatctaccttatcaaaagatccaaatgaaaagagagatcacatggtgttattagagtttgtaactgctgctggaattacacatggcatggatgaattatacaaagctagctcaggaggaggatcaggaggatcagggggaggatcc*ATGgcaggaaaagaagcaactttagttttattagatgtaggtgcctcaatgtatgaaccatacaagtaggctcaaggaaagaaaatcactagattagaattagctgttgattgcatagggatgatgatttaataaaagatctttaattacaagaatcatgaagttggattagttttgtttggaacagaggacgctgaggatgggaatactttttacatctaaacattgtcgtctccagatctagagttttatagaaatctgacagaattacctaatcatgatatacctaaaataattggtggagatatttttgatgctttggacaaatcagttagcactttagatgagtatgtgaagaccaagaagatggataagaagatatttgtgctcacagcaggatttggttagacagaatataatgagaagaagattgcaaagcttattaaaatgattgaaaaagtagatgtgaagatcaatttcatagcattggatttcatgaatgaatatgatgctgaattggatgatccttccaaacctgagaatcaagaaactctaaatgatcgaatgcttaatgctgtttacgaaagttaagagcaatcaatcaattcacgtttagtttattacatggttcaagaactaaggagtcatatgagaatattcccagctaatatcgcatttgaattgtattcacaatttcacaccaaacaaatgcaagcaagagc****ctc****atttagaggagattttcaaataaatgatgagacatccatctctgtattggtttacaaaagatgcacagaggagaaattaccaagtttaaaaaagcattctgctactggagaattcagcagtgaacccactagaaatgtagttagaaatgacaccattcattacaatccagaagacccaaacatgactccaattgaaagagagaatatcattaagggttacttatatggcagaagcctaattcctgtggatagtataatggaagataaaatgaagtattaatgtgttaggtcattttaattattgggatttgttgacaagtcataaattcctagacattattttatgtctagtgtggacatggttgtagctattgattgtgaaaaagcaaagaaatctttatcttctttgatcatagccttaattgccacaaagaaaattgctattgcaagatttgttggcagagaaaagagttctccaaaaatggtggtgttattgcctcataaatcaaagagctattagtgtttttggatgattgccttacccacttctgaagacatcaggcatttccaatttgctgctttaagaaagtccactcctcatcaataaatagctgtagcctccttgattgataaaatggatctagaagcacttccaaacgaatctggagaaccagaagaattacttaaaatgaaatacattgcaaatccaactagacaatatttctaataagtagtcatgcataaagctatcacaagaactgatgtcattcctccaatctctcctttgattctagaatatttgcatcccgaataacgagtatacaattatgcttaggatgctatatagagagtgaagaatgcttttaaattcaaagttaatgaaatcaagaaaccctaagataaaaaagtattttggaaacaattattcgatgaacaaactacataataacaacaagcttaacaataaatagaggaagaagtggttgaaatcaatagagaggaggaagagatggtcaatatgtttgccaaataaaaattgggattcaatgatgatatcatttaagaaattggttctgtggatccaatctcagatttcaagaaaatgatcactgaaaagagagtcgatttggttgattctgcccttcaacaaattcagaaggttataattggattagtcgattaatcagtaaaaggcagtttcttccccaaggccttggaatgcttaaaagaaatgagaagagcttgtatttcagaagatgaagctccagttttcaacaagttcctctttgtacttaaggataagtacaatcaatctatattttgggcccaaattgtacaataaggcatcactttgataagcaacatagagaattaaaaatcaggtgtcactgctgaggaagcataagatgtatatatatttaattaattattagttcctgaacaaagaagataacaaacattagtaaatggttgatcaattataacatgaggaagaagatttattagctgatattgaa****TGAtttattattagtctatatctaacaattttattccaatttttatattaataa***gagctcgaattcgtaatcatggtcatagctgtttcctgtgtgaaattgttatccgctcacaattccacacaacatacgagccggaagcataaagtgtaaagcctggggtgcctaatgagtgagctaactcacattaattgcgttgcgctcactgcccgctttccagtcgggaaacctgtcgtgccagctgcattaatgaatcggccaacgcgcggggagaggcggtttgcgtattgggcgctcttccgcttcctcgctcactgactcgctgcgctcggtcgttcggctgcggcgagcggtatcagctcactcaaaggcggtaatacggttatccacagaatcaggggataacgcaggaaagaacatgtgagcaaaaggccagcaaaaggccaggaaccgtaaaaaggccgcgttgctggcgtttttccataggctccgcccccctgacgagcatcacaaaaatcgacgctcaagtcagaggtggcgaaacccgacaggactataaagataccaggcgtttccccctggaagctccctcgtgcgctctcctgttccgaccctgccgcttaccggatacctgtccgcctttctcccttcgggaagcgtggcgctttctcatagctcacgctgtaggtatctcagttcggtgtaggtcgttcgctccaagctgggctgtgtgcacgaaccccccgttcagcccgaccgctgcgccttatccggtaactatcgtcttgagtccaacccggtaagacacgacttatcgccactggcagcagccactggtaacaggattagcagagcgaggtatgtaggcggtgctacagagttcttgaagtggtggcctaactacggctacactagaaggacagtatttggtatctgcgctctgctgaagccagttaccttcggaaaaagagttggtagctcttgatccggcaaacaaaccaccgctggtagcggtggtttttttgtttgcaagcagcagattacgcgcagaaaaaaaggatctcaagaagatcctttgatcttttctacggggtctgacgctcagtggaacgaaaactcacgttaagggattttggtcatgagattatcaaaaaggatcttcacctagatccttttaaattaaaaatgaagttttaaatcaatctaaagtatatatgagtaaacttggtctgacagttaccaatgcttaatcagtgaggcacctatctcagcgatctgtctatttcgttcatccatagttgcctgactccccgtcgtgtagataactacgatacgggagggcttaccatctggccccagtgctgcaatgataccgcgagacccacgctcaccggctccagatttatcagcaataaaccagccagccggaagggccgagcgcagaagtggtcctgcaactttatccgcctccatccagtctattaattgttgccgggaagctagagtaagtagttcgccagttaatagtttgcgcaacgttgttgccattgctacaggcatcgtggtgtcacgctcgtcgtttggtatggcttcattcagctccggttcccaacgatcaaggcgagttacatgatcccccatgttgtgcaaaaaagcggttagctccttcggtcctccgatcgttgtcagaagtaagttggccgcagtgttatcactcatggttatggcagcactgcataattctcttactgtcatgccatccgtaagatgcttttctgtgactggtgagtactcaaccaagtcattctgagaatagtgtatgcggcgaccgagttgctcttgcccggcgtcaatacgggataataccgcgccacatagcagaactttaaaagtgctcatcattggaaaacgttcttcggggcgaaaactctcaaggatcttaccgctgttgagatccagttcgatgtaacccactcgtgcacccaactgatcttcagcatcttttactttcaccagcgtttctgggtgagcaaaaacaggaaggcaaaatgccgcaaaaaagggaataagggcgacacggaaatgttgaatactcatactcttcctttttcaatattattgaagcatttatcagggttattgtctcatgagcggatacatatttgaatgtatttagaaaaataaacaaataggggttccgcgcacatttccccgaaaagtgccacctgacgtctaagaaaccattattatcatgacattaacctataaaaataggcgtatcacgaggccctttcgtc

>pUC18-FLAG-Ku80ca chimera (RNAiResistant)

tcgcgcgtttcggtgatgacggtgaaaacctctgacacatgcagctcccggagacggtcacagcttgtctgtaagcggatgccgggagcagacaagcccgtcagggcgcgtcagcgggtgttggcgggtgtcggggctggcttaactatgcggcatcagagcagattgtactgagagtgcaccatatgcggtgtgaaataccgcacagatgcgtaaggagaaaataccgcatcaggcgccattcgccattcaggctgcgcaactgttgggaagggcgatcggtgcgggcctcttcgctattacgccagctggcgaaagggggatgtgctgcaaggcgattaagttgggtaacgccagggttttcccagtcacgacgttgtaaaacgacggccagtgccaagcttgcatgc***ttaaatagataaataatacaggcaattttttaatatacaaatttcaataatgaatttgtagatatatataaaaaataagtgcaaag*ATG**aattctaga**gactacaaagaccatgacggtgattataaagatcatgacatcgactacaaggatgacgatgataag**ggatcc*atgtctggaaaagaagctactctaatattattggatgttggagcctcaatgtatggtcaatattaatagggaggaagtaaaaagttgagcagattggaattggcagtagattgtttaggattgatgattcaataaaagatttttaattataagaatcacgaagtcggattgattttatttggaacagaagaagctcctgatggcaagacattgtacatccaagatttgtcaatcccagatttagatttctttaggaatattagtgatttacctaaccatgatgttggataataagtgggtggagatatatttgatgcattggacaaggcagtacatgctttagatgatcatgcaaagacaaaaaaaatggaaaagaagatctttatattaacagctggttgtggacagactgattacagtgaaaagtagataaccaaattaatcaagatgattgagaaagtggatgttaagataaatttcatagctcttgattttatgaatgattacaatggagatatggatgatcctgaaaaacctgaagaattcgaagctcttaacaataggatgctaacagcctcatatcagtgttaggaacaatccattaattcacgttacgttttcttgatggttcaagagttgaggaataacatgaggatatttccagctaatgttgcttttgagttatattcccagtttcatactagatcactataggctagagcctcatttagaggagattttcaaataaatgatgagacatccatctctgtattggtttacaaaagatgcacagaggagaaattaccaagtttaaaaaagcattctgctactggagaattcagcagtgaacccactagaaatgtagttagaaatgacaccattcattacaatccagaagacccaaacatgactccaattgaaagagagaatatcattaagggttacttatatggcagaagcctaattcctgtggatagtataatggaagataaaatgaagtattaatgtgttaggtcattttaattattgggatttgttgacaagtcataaattcctagacattattttatgtctagtgtggacatggttgtagctattgattgtgaaaaagcaaagaaatctttatcttctttgatcatagccttaattgccacaaagaaaattgctattgcaagatttgttggcagagaaaagagttctccaaaaatggtggtgttattgcctcataaatcaaagagctattagtgtttttggatgattgccttacccacttctgaagacatcaggcatttccaatttgctgctttaagaaagtccactcctcatcaataaatagctgtagcctccttgattgataaaatggatctagaagcacttccaaacgaatctggagaaccagaagaattacttaaaatgaaatacattgcaaatccaactagacaatatttctaataagtagtcatgcataaagctatcacaagaactgatgtcattcctccaatctctcctttgattctagaatatttgcatcccgaataacgagtatacaattatgcttaggatgctatatagagagtgaagaatgcttttaaattcaaagttaatgaaatcaagaaaccctaagataaaaaagtattttggaaacaattattcgatgaacaaactacataataacaacaagcttaacaataaatagaggaagaagtggttgaaatcaatagagaggaggaagagatggtcaatatgtttgccaaataaaaattgggattcaatgatgatatcatttaagaaattggttctgtggatccaatctcagatttcaagaaaatgatcactgaaaagagagtcgatttggttgattctgcccttcaacaaattcagaaggttataattggattagtcgattaatcagtaaaaggcagtttcttccccaaggccttggaatgcttaaaagaaatgagaagagcttgtatttcagaagatgaagctccagttttcaacaagttcctctttgtacttaaggataagtacaatcaatctatattttgggcccaaattgtacaataaggcatcactttgataagcaacatagagaattaaaaatcaggtgtcactgctgaggaagcataagatgtatatatatttaattaattattagttcctgaacaaagaagataacaaacattagtaaatggttgatcaattataacatgaggaagaagatttattagctgatattgaa****TGAtttattattagtctatatctaacaattttattccaatttttatattaataa***gagctcgaattcgtaatcatggtcatagctgtttcctgtgtgaaattgttatccgctcacaattccacacaacatacgagccggaagcataaagtgtaaagcctggggtgcctaatgagtgagctaactcacattaattgcgttgcgctcactgcccgctttccagtcgggaaacctgtcgtgccagctgcattaatgaatcggccaacgcgcggggagaggcggtttgcgtattgggcgctcttccgcttcctcgctcactgactcgctgcgctcggtcgttcggctgcggcgagcggtatcagctcactcaaaggcggtaatacggttatccacagaatcaggggataacgcaggaaagaacatgtgagcaaaaggccagcaaaaggccaggaaccgtaaaaaggccgcgttgctggcgtttttccataggctccgcccccctgacgagcatcacaaaaatcgacgctcaagtcagaggtggcgaaacccgacaggactataaagataccaggcgtttccccctggaagctccctcgtgcgctctcctgttccgaccctgccgcttaccggatacctgtccgcctttctcccttcgggaagcgtggcgctttctcatagctcacgctgtaggtatctcagttcggtgtaggtcgttcgctccaagctgggctgtgtgcacgaaccccccgttcagcccgaccgctgcgccttatccggtaactatcgtcttgagtccaacccggtaagacacgacttatcgccactggcagcagccactggtaacaggattagcagagcgaggtatgtaggcggtgctacagagttcttgaagtggtggcctaactacggctacactagaaggacagtatttggtatctgcgctctgctgaagccagttaccttcggaaaaagagttggtagctcttgatccggcaaacaaaccaccgctggtagcggtggtttttttgtttgcaagcagcagattacgcgcagaaaaaaaggatctcaagaagatcctttgatcttttctacggggtctgacgctcagtggaacgaaaactcacgttaagggattttggtcatgagattatcaaaaaggatcttcacctagatccttttaaattaaaaatgaagttttaaatcaatctaaagtatatatgagtaaacttggtctgacagttaccaatgcttaatcagtgaggcacctatctcagcgatctgtctatttcgttcatccatagttgcctgactccccgtcgtgtagataactacgatacgggagggcttaccatctggccccagtgctgcaatgataccgcgagacccacgctcaccggctccagatttatcagcaataaaccagccagccggaagggccgagcgcagaagtggtcctgcaactttatccgcctccatccagtctattaattgttgccgggaagctagagtaagtagttcgccagttaatagtttgcgcaacgttgttgccattgctacaggcatcgtggtgtcacgctcgtcgtttggtatggcttcattcagctccggttcccaacgatcaaggcgagttacatgatcccccatgttgtgcaaaaaagcggttagctccttcggtcctccgatcgttgtcagaagtaagttggccgcagtgttatcactcatggttatggcagcactgcataattctcttactgtcatgccatccgtaagatgcttttctgtgactggtgagtactcaaccaagtcattctgagaatagtgtatgcggcgaccgagttgctcttgcccggcgtcaatacgggataataccgcgccacatagcagaactttaaaagtgctcatcattggaaaacgttcttcggggcgaaaactctcaaggatcttaccgctgttgagatccagttcgatgtaacccactcgtgcacccaactgatcttcagcatcttttactttcaccagcgtttctgggtgagcaaaaacaggaaggcaaaatgccgcaaaaaagggaataagggcgacacggaaatgttgaatactcatactcttcctttttcaatattattgaagcatttatcagggttattgtctcatgagcggatacatatttgaatgtatttagaaaaataaacaaataggggttccgcgcacatttccccgaaaagtgccacctgacgtctaagaaaccattattatcatgacattaacctataaaaataggcgtatcacgaggccctttcgtc

>pUC18-FLAG-Ku80ac chimera (RNAiResistant)

tcgcgcgtttcggtgatgacggtgaaaacctctgacacatgcagctcccggagacggtcacagcttgtctgtaagcggatgccgggagcagacaagcccgtcagggcgcgtcagcgggtgttggcgggtgtcggggctggcttaactatgcggcatcagagcagattgtactgagagtgcaccatatgcggtgtgaaataccgcacagatgcgtaaggagaaaataccgcatcaggcgccattcgccattcaggctgcgcaactgttgggaagggcgatcggtgcgggcctcttcgctattacgccagctggcgaaagggggatgtgctgcaaggcgattaagttgggtaacgccagggttttcccagtcacgacgttgtaaaacgacggccagtgccaagcttgcatgc***ttaaatagataaataatacaggcaattttttaatatacaaatttcaataatgaatttgtagatatatataaaaaataagtgcaaag*ATG**aattctagagactacaaagaccatgacggtgattataaagatcatgacatcgactacaaggatgacgatgataagggatcc*atggcaggaaaagaagcaactttagttttattagatgtaggtgcctcaatgtatgaaccatacaagtaggctcaaggaaagaaaatcactagattagaattagctgttgattgcatagggatgatgatttaataaaagatctttaattacaagaatcatgaagttggattagttttgtttggaacagaggacgctgaggatgggaatactttttacatctaaacattgtcgtctccagatctagagttttatagaaatctgacagaattacctaatcatgatatacctaaaataattggtggagatatttttgatgctttggacaaatcagttagcactttagatgagtatgtgaagaccaagaagatggataagaagatatttgtgctcacagcaggatttggttagacagaatataatgagaagaagattgcaaagcttattaaaatgattgaaaaagtagatgtgaagatcaatttcatagcattggatttcatgaatgaatatgatgctgaattggatgatccttccaaacctgagaatcaagaaactctaaatgatcgaatgcttaatgctgtttacgaaagttaagagcaatcaatcaattcacgtttagtttattacatggttcaagaactaaggagtcatatgagaatattcccagctaatatcgcatttgaattgtattcacaatttcacaccaaacaaatgcaagcaagagcctcatttaggggggacttccagattaacgatgaaatctctgtgcaagtgctaatttataagcgatgtttcgaggaaagattgccaactctaagaaagcattcaacccttggcgaattccaaacagatactaataaaaaccatgtgagaaatgatcttatctactataatccagaggatcctaatatgacaccaatcgaaaaagacaacatcataaggggatattaatatggcaggaatctcgtaccagttgattaaataatggaggataaaatgaagtactagtgtcctcgttaattttaattattaggctttgtagatcgatcccatatacctagatattattatacctcaactgttgacatggtcattgctgtagaaaatcagaagtaatagaaagctctggctgctctagtcattgcactcattgcaactaggaaagtagctcttgccagatttgtaggcagagagaaaacagcacctaaattgattatgttattaccccataaatccaagaactcttagtgcttctggatgatatccttgcctacaactgaagacatcaggcattttcaatttgctgctttgaagagatctactcctccataatagatggcagtctctgctatgattgactgtatggatctagaaaagatgcccacggaagatggacagtttgaagaacttcttaaaatgaagtatgttgccaatcctacgagataatacttccaataagtggtcatgcacaaagccatcactagatctgatgtattgcctcctatctcccctctcatcttggagtatttacatcctgaaaagagggtttacgattatgcaaaagaggcacttcaaaaagtcaaggctgctttcaaattcaaaatcaacgaaatcaaaaaacaaggggataagaaagtgttttggaaataactatttgaagatcaatctacagaataaatttaatagtaagtggaggatgaagtagttgaaatcaattaagaagaagaagagatggttaatatgtttgccaaataaaaattaggattcaatgacgatatagttaaggaaatcggcactgttgatcccacttcagatttcaggaagatgattactgaaaagagagtcgacttagttgacactgccttacaataaattcaaaaagtcattatttaatttgtggatcaatctctcaaaggcagtttctatccaaaagcacttgagtgtctcaaagaaatgaggaaagcctgtatcacagaagatgaagccccagtattcaataaatacttgcatgttcttaaagagaagtacagtcaacttgtcttctgggctcagattgtccaataaggaatcactttaatcagcaacattgagaattagaaatctcatgtttctgttgatgaagcacaagaagtaattcattattataatattagttcctaaataaagaggatattagtcataaataattagttgatcaattataacacgaagaagaggatctattggctgaaattgat****TGAtttattattagtctatatctaacaattttattccaatttttatattaataa***gagctcgcatgctctaattaaaccaagaacacgctgaattccatgctgcctaggttgttctttttcttgaatatttgcattcaaaccgtatattatatcggagtttgaattcatagcatttaatgattggatctgatgattacctaagataaattgtttttattttacagtattacacgaaagaaaaagaacagcttcatttcaccgattatgtcaaatcccaactcaagcttgagctcgaattcgtaatcatggtcatagctgtttcctgtgtgaaattgttatccgctcacaattccacacaacatacgagccggaagcataaagtgtaaagcctggggtgcctaatgagtgagctaactcacattaattgcgttgcgctcactgcccgctttccagtcgggaaacctgtcgtgccagctgcattaatgaatcggccaacgcgcggggagaggcggtttgcgtattgggcgctcttccgcttcctcgctcactgactcgctgcgctcggtcgttcggctgcggcgagcggtatcagctcactcaaaggcggtaatacggttatccacagaatcaggggataacgcaggaaagaacatgtgagcaaaaggccagcaaaaggccaggaaccgtaaaaaggccgcgttgctggcgtttttccataggctccgcccccctgacgagcatcacaaaaatcgacgctcaagtcagaggtggcgaaacccgacaggactataaagataccaggcgtttccccctggaagctccctcgtgcgctctcctgttccgaccctgccgcttaccggatacctgtccgcctttctcccttcgggaagcgtggcgctttctcatagctcacgctgtaggtatctcagttcggtgtaggtcgttcgctccaagctgggctgtgtgcacgaaccccccgttcagcccgaccgctgcgccttatccggtaactatcgtcttgagtccaacccggtaagacacgacttatcgccactggcagcagccactggtaacaggattagcagagcgaggtatgtaggcggtgctacagagttcttgaagtggtggcctaactacggctacactagaaggacagtatttggtatctgcgctctgctgaagccagttaccttcggaaaaagagttggtagctcttgatccggcaaacaaaccaccgctggtagcggtggtttttttgtttgcaagcagcagattacgcgcagaaaaaaaggatctcaagaagatcctttgatcttttctacggggtctgacgctcagtggaacgaaaactcacgttaagggattttggtcatgagattatcaaaaaggatcttcacctagatccttttaaattaaaaatgaagttttaaatcaatctaaagtatatatgagtaaacttggtctgacagttaccaatgcttaatcagtgaggcacctatctcagcgatctgtctatttcgttcatccatagttgcctgactccccgtcgtgtagataactacgatacgggagggcttaccatctggccccagtgctgcaatgataccgcgagacccacgctcaccggctccagatttatcagcaataaaccagccagccggaagggccgagcgcagaagtggtcctgcaactttatccgcctccatccagtctattaattgttgccgggaagctagagtaagtagttcgccagttaatagtttgcgcaacgttgttgccattgctacaggcatcgtggtgtcacgctcgtcgtttggtatggcttcattcagctccggttcccaacgatcaaggcgagttacatgatcccccatgttgtgcaaaaaagcggttagctccttcggtcctccgatcgttgtcagaagtaagttggccgcagtgttatcactcatggttatggcagcactgcataattctcttactgtcatgccatccgtaagatgcttttctgtgactggtgagtactcaaccaagtcattctgagaatagtgtatgcggcgaccgagttgctcttgcccggcgtcaatacgggataataccgcgccacatagcagaactttaaaagtgctcatcattggaaaacgttcttcggggcgaaaactctcaaggatcttaccgctgttgagatccagttcgatgtaacccactcgtgcacccaactgatcttcagcatcttttactttcaccagcgtttctgggtgagcaaaaacaggaaggcaaaatgccgcaaaaaagggaataagggcgacacggaaatgttgaatactcatactcttcctttttcaatattattgaagcatttatcagggttattgtctcatgagcggatacatatttgaatgtatttagaaaaataaacaaataggggttccgcgcacatttccccgaaaagtgccacctgacgtctaagaaaccattattatcatgacattaacctataaaaataggcgtatcacgaggccctttcgtc
