# Supplementary material for: Functional diversification of Paramecium Ku80 paralogs safeguards genome integrity during precise programmed DNA elimination
Source: PLoS Genet. 2020 Apr 16;16(4):e1008723. doi: 10.1371/journal.pgen.1008723 (PMC7161955; doi:10.1371/journal.pgen.1008723)
Supplement: S2 File — (DOCX) [file pgen.1008723.s018.docx]

**Supporting File 2. Sequence of the pFastBAC derivative harboring the *HA-KU80a* transgene used for protein expression in insect cells**

The *HA-KU80a* gene is in capital letters, with the sequence encoding the HA tag in red.

>pFast-HA-KU80a

gacgcgccctgtagcggcgcattaagcgcggcgggtgtggtggttacgcgcagcgtgaccgctacacttgccagcgccctagcgcccgctcctttcgctttcttcccttcctttctcgccacgttcgccggctttccccgtcaagctctaaatcgggggctccctttagggttccgatttagtgctttacggcacctcgaccccaaaaaacttgattagggtgatggttcacgtagtgggccatcgccctgatagacggtttttcgccctttgacgttggagtccacgttctttaatagtggactcttgttccaaactggaacaacactcaaccctatctcggtctattcttttgatttataagggattttgccgatttcggcctattggttaaaaaatgagctgatttaacaaaaatttaacgcgaattttaacaaaatattaacgtttacaatttcaggtggcacttttcggggaaatgtgcgcggaacccctatttgtttatttttctaaatacattcaaatatgtatccgctcatgagacaataaccctgataaatgcttcaataatattgaaaaaggaagagtatgagtattcaacatttccgtgtcgcccttattcccttttttgcggcattttgccttcctgtttttgctcacccagaaacgctggtgaaagtaaaagatgctgaagatcagttgggtgcacgagtgggttacatcgaactggatctcaacagcggtaagatccttgagagttttcgccccgaagaacgttttccaatgatgagcacttttaaagttctgctatgtggcgcggtattatcccgtattgacgccgggcaagagcaactcggtcgccgcatacactattctcagaatgacttggttgagtactcaccagtcacagaaaagcatcttacggatggcatgacagtaagagaattatgcagtgctgccataaccatgagtgataacactgcggccaacttacttctgacaacgatcggaggaccgaaggagctaaccgcttttttgcacaacatgggggatcatgtaactcgccttgatcgttgggaaccggagctgaatgaagccataccaaacgacgagcgtgacaccacgatgcctgtagcaatggcaacaacgttgcgcaaactattaactggcgaactacttactctagcttcccggcaacaattaatagactggatggaggcggataaagttgcaggaccacttctgcgctcggcccttccggctggctggtttattgctgataaatctggagccggtgagcgtgggtctcgcggtatcattgcagcactggggccagatggtaagccctcccgtatcgtagttatctacacgacggggagtcaggcaactatggatgaacgaaatagacagatcgctgagataggtgcctcactgattaagcattggtaactgtcagaccaagtttactcatatatactttagattgatttaaaacttcatttttaatttaaaaggatctaggtgaagatcctttttgataatctcatgaccaaaatcccttaacgtgagttttcgttccactgagcgtcagaccccgtagaaaagatcaaaggatcttcttgagatcctttttttctgcgcgtaatctgctgcttgcaaacaaaaaaaccaccgctaccagcggtggtttgtttgccggatcaagagctaccaactctttttccgaaggtaactggcttcagcagagcgcagataccaaatactgtccttctagtgtagccgtagttaggccaccacttcaagaactctgtagcaccgcctacatacctcgctctgctaatcctgttaccagtggctgctgccagtggcgataagtcgtgtcttaccgggttggactcaagacgatagttaccggataaggcgcagcggtcgggctgaacggggggttcgtgcacacagcccagcttggagcgaacgacctacaccgaactgagatacctacagcgtgagcattgagaaagcgccacgcttcccgaagggagaaaggcggacaggtatccggtaagcggcagggtcggaacaggagagcgcacgagggagcttccagggggaaacgcctggtatctttatagtcctgtcgggtttcgccacctctgacttgagcgtcgatttttgtgatgctcgtcaggggggcggagcctatggaaaaacgccagcaacgcggcctttttacggttcctggccttttgctggccttttgctcacatgttctttcctgcgttatcccctgattctgtggataaccgtattaccgcctttgagtgagctgataccgctcgccgcagccgaacgaccgagcgcagcgagtcagtgagcgaggaagcggaagagcgcctgatgcggtattttctccttacgcatctgtgcggtatttcacaccgcagaccagccgcgtaacctggcaaaatcggttacggttgagtaataaatggatgccctgcgtaagcgggtgtgggcggacaataaagtcttaaactgaacaaaatagatctaaactatgacaataaagtcttaaactagacagaatagttgtaaactgaaatcagtccagttatgctgtgaaaaagcatactggacttttgttatggctaaagcaaactcttcattttctgaagtgcaaattgcccgtcgtattaaagaggggcgtggccaagggcatggtaaagactatattcgcggcgttgtgacaatttaccgaacaactccgcggccgggaagccgatctcggcttgaacgaattgttaggtggcggtacttgggtcgatatcaaagtgcatcacttcttcccgtatgcccaactttgtatagagagccactgcgggatcgtcaccgtaatctgcttgcacgtagatcacataagcaccaagcgcgttggcctcatgcttgaggagattgatgagcgcggtggcaatgccctgcctccggtgctcgccggagactgcgagatcatagatatagatctcactacgcggctgctcaaacctgggcagaacgtaagccgcgagagcgccaacaaccgcttcttggtcgaaggcagcaagcgcgatgaatgtcttactacggagcaagttcccgaggtaatcggagtccggctgatgttgggagtaggtggctacgtctccgaactcacgaccgaaaagatcaagagcagcccgcatggatttgacttggtcagggccgagcctacatgtgcgaatgatgcccatacttgagccacctaactttgttttagggcgactgccctgctgcgtaacatcgttgctgctgcgtaacatcgttgctgctccataacatcaaacatcgacccacggcgtaacgcgcttgctgcttggatgcccgaggcatagactgtacaaaaaaacagtcataacaagccatgaaaaccgccactgcgccgttaccaccgctgcgttcggtcaaggttctggaccagttgcgtgagcgcatacgctacttgcattacagtttacgaaccgaacaggcttatgtcaactgggttcgtgccttcatccgtttccacggtgtgcgtcacccggcaaccttgggcagcagcgaagtcgaggcatttctgtcctggctggcgaacgagcgcaaggtttcggtctccacgcatcgtcaggcattggcggccttgctgttcttctacggcaaggtgctgtgcacggatctgccctggcttcaggagatcggaagacctcggccgtcgcggcgcttgccggtggtgctgaccccggatgaagtggttcgcatcctcggttttctggaaggcgagcatcgtttgttcgcccaggactctagctatagttctagtggttggctacgtatactccggaatattaatagatcatggagataattaaaatgataaccatctcgcaaataaataagtattttactgttttcgtaacagttttgtaataaaaaaacctataaatattccggattattcataccgtcccaccatcgggcgcggatct**atg**tacccgtatgatgttccggattatgcgggatccatggcgggcaaagaagctaccctggttctgctggatgttggagcgtcaatgtatgaaccgtataaacaggctcagggcaagaaaatcacccgcctggaactggcggtggactgcattggcatgatgatccagcagaaaatctttaactacaaaaatcacgaggttggtctggtactcttcggcaccgaagatgcggaagatgggaatactttctacattcaaaccttgagcagtccggatctcgagttctatcggaacctcaccgagttacccaatcacgatattccgaaaatcattggcggcgatatctttgatgccctggacaaaagcgtgtcgaccttagacgaatacgtgaaaaccaagaaaatggacaagaagatctttgtcctgactgccggttttggtcaaacggaatataatgaaaaaaaaatcgccaaactgattaaaatgatcgagaaagtggacgtgaaaattaactttatcgcgttagactttatgaacgaatatgacgcagaattagatgatccgagtaaacccgaaaatcaggagacactcaacgaccgtatgctgaacgcggtctatgaatctcaggaacagtcgattaattctcggttagtatactacatggtacaagagttgcgctcccatatgcgtattttcccggccaacattgcgtttgaactgtatagccagttccacacgaaacagatgcaagcacgtgcaagctttcgcggtgattttcagatcaacgatgaaacgagcatctccgtcttggtctataaacgttgcacggaagagaagttaccttcgctgaagaaacacagtgcaacaggcgaattctcaagcgaacctacacgcaacgttgtccgcaacgacacgatccactataacccagaagatccaaacatgacgccgattgaacgcgaaaacatcattaaagggtacctttatggccgcagcctgattccggtggattcgattatggaggacaaaatgaaataccagtgtgttcgttccttccaactgctgggatttgtagacaaatctcagattccgcgtcattactttatgtcgtctgttgatatggttgtggccattgattgtgagaaagccaaaaaatccctttcaagcctgattattgcgctgattgcaactaagaaaatcgcgattgcgcgtttcgtggggcgtgagaaatcgagtccgaaaatggtggtactgcttccgcataagagcaaaagctaccaatgcttctggatgattgcccttcccaccagtgaggatattcgccattttcaatttgcagcactgcgcaaaagcaccccacatcagcagattgccgttgcatctctcatcgacaagatggatctggaggcacttccgaatgaaagcggtgaacccgaagaactgctgaaaatgaagtatattgcgaatcccacgcgccaatacttccagcaggtagtgatgcacaaagcgatcacacgcacggatgtgattcctcctattagtccgttgatcctggaatatctccatcccgaacaacgcgtgtataattatgctcaggacgccattcagcgcgttaagaatgcctttaaattcaaggttaacgaaatcaagaaaccacaagataagaaagtgttttggaaacagttatttgatgaacaaaccactcagcaacagcaagcccagcaacagatcgaggaggaggttgtggaaatcaatcgggaggaagaagagatggtcaacatgtttgcgaaacagaaattgggctttaatgacgatattatccaggaaattggtagtgtggacccgatttccgattttaagaaaatgattaccgagaaacgtgttgatctggttgactcggccctgcaacagatccagaaagtgatcatcggcctggtcgatcagtctgtgaaaggttctttctttccaaaagctctggaatgtctgaaagaaatgcgtcgtgcatgcatttctgaagatgaagctccagtgttcaataaattcttattcgtcctcaaagataagtacaatcagagcatcttttgggctcagatcgtacagcagggtatcacactgatttcaaacattgaaaaccagaagtcaggagtcaccgccgaggaggctcaggatttcttgaataaagaagataataaacaccagcagatggtggaccaattacagcacgaagaagaagatttactggcggacattgaa**tga**gtctagagcctgcagtctcgaggcatgcggtaccaagcttgtcgagaagtactagaggatcataatcagccataccacatttgtagaggttttacttgctttaaaaaacctcccacacctccccctgaacctgaaacataaaatgaatgcaattgttgttgttaacttgtttattgcagcttataatggttacaaataaagcaatagcatcacaaatttcacaaataaagcatttttttcactgcattctagttgtggtttgtccaaactcatcaatgtatcttatcatgtctggatctgatcactgcttgagcctaggagatccgaaccagataagtgaaatctagttccaaactattttgtcatttttaattttcgtattagcttacgacgctacacccagttcccatctattttgtcactcttccctaaataatccttaaaaactccatttccacccctcccagttcccaactattttgtccgcccacagcggggcatttttcttcctgttatgtttttaatcaaacatcctgccaactccatgtgacaaaccgtcatcttcggctactttttctctgtcacagaatgaaaatttttctgtcatctcttcgttattaatgtttgtaattgactgaatatcaacgcttatttgcagcctgaatggcgaatgg
